# Global trade responses to shark finning regulations

**DOI:** 10.1038/s41467-026-75625-1

**Published:** 2026-07-31

**Authors:** Echelle S. Burns, Sara Orofino, Kaiwen Wang, Darcy Bradley, Nidhi G. D’Costa, Leonardo Manir Feitosa, Laurenne Schiller, Boris Worm, Jessica A. Gephart, Gavin G. McDonald

**Affiliations:** 1 Marine Science Institute, University of California, Santa Barbara, Santa Barbara, CA USA; 2 Bren School of Environmental Science & Management, University of California, Santa Barbara, Santa Barbara, CA USA; 3 Environmental Markets Lab, University of California, Santa Barbara, Santa Barbara, CA USA; 4 California Oceans Program, The Nature Conservancy in California, Sacramento, CA USA; 5 School of Agricultural Economics and Rural Development, Renmin University of China, Beijing, China; 6 The Manta Trust, Catemwood House, Norwood Lane, Corscombe, Dorset, UK; 7 Biology Department, Dalhousie University, Halifax, NS Canada; 8 School of Aquatic and Fishery Science, University of Washington, Seattle, WA USA

**Keywords:** Environmental economics, Ecology, Social sciences

## Abstract

Global shark mortality continues to rise despite increasing regulations aimed at limiting shark fishing and trade. Regulatory measures may influence shark product markets, but this relationship has been difficult to quantify because of fragmented and poorly resolved data. We present a global analysis evaluating the effects of domestic shark finning regulations on trade in shark products across 188 countries. Using causal inference methods and the Aquatic Resource Trade in Species database (2007–2020), we find weak and statistically insignificant effects of finning regulations on overall shark trade volumes. However, long-term trends suggest that countries adopting finning regulations tend to reduce exports, modestly increase imports, and maintain relatively stable domestic consumption. Our framework provides a broader approach for investigating the economic pathways and drivers of mortality associated with sharks and other traded wildlife species.

## Abstract (web summary)

The study finds limited evidence that shark finning regulations alter global shark trade volumes. While overall effects are not significant, long-term trends suggest gradual trade shifts, including declining exports and small changes in imports from regulated countries.

## Introduction

The rapid decline of sharks and their relatives (Class Chondrichthyes) is one of the world’s most pressing biodiversity issues and can result in severe, negative impacts on ocean ecosystems^1–3^. Shark species have inherent life history characteristics that make them susceptible to abrupt population declines, including late maturation, longevity, and low fecundity. Recent studies indicate that more than one-third of chondrichthyan species face possible extinction^4^, driven by their over-exploitation in global fisheries^5,6^, where they are caught as unintended ‘bycatch’, or directly targeted to supply a growing demand for shark products such as fins, meat, and oil.

Global efforts to curb shark mortality include national, regional, and international regulations aimed at protecting sharks from fishing (e.g., through species-specific retention bans or spatial shark sanctuaries), curbing unethical and wasteful fishing practices (e.g., prohibiting shark finning where the valuable fins are cut off and the body discarded), and/or regulating international trade in shark products (e.g., restrictions under the Convention on International Trade in Endangered Species of Wild Fauna and Flora [CITES] or national trade bans). Despite a rapid increase in these efforts over past decades, shark mortality remains high in most regions and globally^7^. Addressing the deeper economic drivers of observed shark mortality trends is challenging because regulations on fishing practices can influence trade dynamics and demand for traded shark products can, in turn, affect fishing practices (e.g.^8,9^). Here, quantifying the relationship between finning regulations and trade responses may offer new insight into how management decisions shape global trade and consumption patterns and, ultimately, conservation outcomes.

Country-specific shark finning regulations provide a useful case study to illustrate such linkages between management and trade flows, as they apply broadly to all shark species, are widely adopted, and have a direct connection to traded shark products associated with each country. Shark fins have historically been the most valuable shark product due to high demand from East and Southeast Asian markets^10^. Fishers who did not traditionally target sharks for meat often engaged in shark finning, the practice of removing and retaining shark fins and discarding the carcasses (or live animals) at sea. This method allowed fishers to maintain vessel storage space for primary target species, such as tuna, while also participating in the lucrative shark fin trade^11–13^. Shark finning regulations were widely implemented to eliminate this wasteful fishing practice, requiring fins to be naturally attached (FNA) or artificially attached (FAA, e.g., with wires or ropes) to carcasses onboard; or landed in a prescribed fin-to-carcass ratio (FCR)^7^. Since these regulations require fishers to either discard or land whole sharks, they may incur one of three possible trade-related outcomes: (1) international trade of shark products increases (whole sharks are now retained, leading to development or expansion of emerging global markets for shark meat^14^); (2) domestic consumption of shark products increases (whole sharks are retained and meat is consumed locally as a cheaper protein source than other commercial fish products^15^), or (3) there is no effect on the global volume of shark meat traded (either because fisheries originally finning sharks now discard them whole at-sea, because regulations are not properly enforced, or because their effects are masked by other market trends). Indeed, the relationship between shark finning regulations and shark trade is not entirely straightforward. The direct results of finning regulations may depend on other regional or national fishing regulations, economic incentives, or changes in market demand, making it difficult to isolate the specific role that shark finning plays^10,16^. For example, countries that participate little in global trade may not experience any changes after regulations are implemented, whereas countries with traditional markets for shark meat, such as Brazil, may experience shifts in trade patterns^16^. Given the variety of potential outcomes, understanding the impact of finning regulations on shark trade has important implications for domestic and international policy. Higher discard rates would necessitate additional management measures that target fishing behavior in order to effectively reduce catch and mortality of discarded sharks, while growth or expansion of meat markets would require additional regulations related to the trade of whole sharks, with particular importance for vulnerable (i.e., species listed as endangered and critically endangered in the International Union for Conservation of Nature Red List) shark species.

The ability to evaluate the impact of regulatory interventions on the trade of shark products has historically been difficult due to complex global supply chains and shortcomings in global trade data. Trade data are aggregated to generic product codes that often lack meaningful taxonomic information (e.g., Harmonized System code 030420 corresponded to frozen fish fillets up to 2002). Trade data are also reported as product-level transactions between importing and exporting countries, without distinguishing trade through intermediate countries, while production data (i.e., fisheries landings) are reported at the species level by the producing country, generally representing the fishing vessel flag. In addition, several species can often be used to produce the same product, and one species could be used to create multiple different products. These issues have prevented accurate tracing of species-specific shark products through the supply chain. Previous efforts to characterize global shark markets have emphasized that these reporting standards make it challenging to trace species-specific shark products through the supply chain^10,14,17^.

To overcome the paucity of taxonomically resolved global trade data, prior research on shark markets has focused on local scales, working with government agencies to characterize the import and export of particular products to individual countries^16,18^, or by using DNA barcoding to properly identify species labeled as sharks^19,20^. However, this approach is time-consuming and infeasible to reproduce at global scales, thereby preventing effective hypothesis testing of the effects of shark regulations on global shark trade. The newly available Aquatic Resource Trade in Species (ARTIS) database offers a promising solution to overcome critical data gaps by using a mass balance approach to reconcile production and trade data at the country-level to estimate the first species/species group disaggregated global database of aquatic product trade from 1996 to 2020^21^. By estimating the live-weight equivalent of relevant primary products (i.e., the total weight of the species that needed to be caught to produce the reported weight of the traded product), ARTIS bridges the gap between production and trade data, enabling estimates of domestic consumption and providing a direct link between shark landings and trade flows^21^.

Here, we leverage the ARTIS database, alongside causal inference techniques, to evaluate the effect of shark finning regulations on the (1) global trade of shark products by volume, and (2) global shark trade network relationships from 2007 to 2020. We first provide a brief overview of the global trade of shark products during the study period. We then analyze whether finning regulations in participating countries have a causal effect on reported volumes of: (1) traded shark products (exports and imports); and (2) apparent domestic consumption of shark products (all in metric tons, live weight equivalent; see Supplementary Table 1 for full definitions). To complement our analysis on trade outcomes, we use network analysis to understand the connectivity of countries participating in the global shark trade. We first characterize the shark trade network during the study period and then apply the same causal inference techniques to analyze whether finning regulations in participating countries have a causal impact on the number of trade partnerships. Our results may serve as a foundation to better understand the impact of regulatory interventions on global shark trade. Furthermore, the methodological approach developed in this paper and the underlying ARTIS database have broad relevance to analyses of global wildlife trade, a major threat to biodiversity worldwide.

## Results

### Global trade of shark products by volume

From 2007 to 2020, the ARTIS database includes 123 unique shark taxonomic identifiers, reported at either the species (∼83% of all taxonomic identifiers), genus (∼9%), family (∼6%), or order (∼2%) level (Supplementary Data 1), traded or consumed under 17 unique product codes (Supplementary Table 2) by 188 countries.

**Fig. 1:**
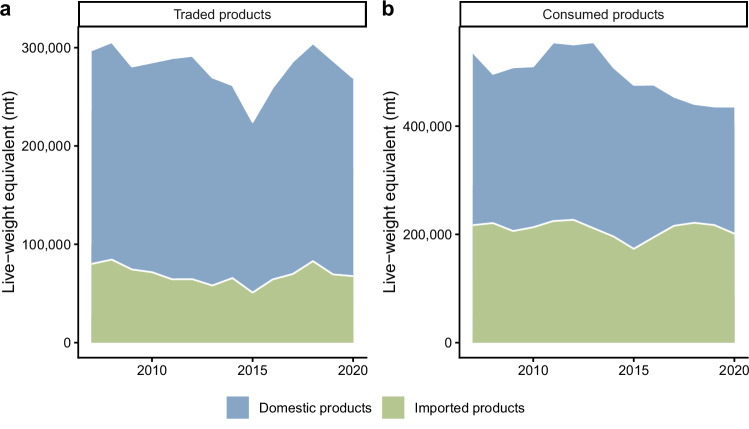
Global trade in shark products. Shown is the total volume of shark products (metric tons, mt) that is (**a**) traded or (**b)** consumed each year. Domestically-sourced products are presented in blue, while imported products are shown in green.

The total volume of traded shark products was relatively stable over time ranging from a minimum of 224,382 metric tons (mt) in 2008 to a maximum of 305,496 mt in 2015 (mean ± standard deviation: 279,323 ± 21,370 mt). Most products were traded directly from the source country to the end market (75% on average) with intermediate processing (i.e., source country to processing country to end market) accounting for a smaller percentage of all traded products (Fig. 1). The majority of shark products consumed over time were sourced domestically (i.e., consumed in the same country the shark was landed; 57% on average). Overall, annual domestic consumption of shark products was approximately double the traded volume of shark products (495,785 ± 43,666 mt; Fig. 1).

**Fig. 2:**
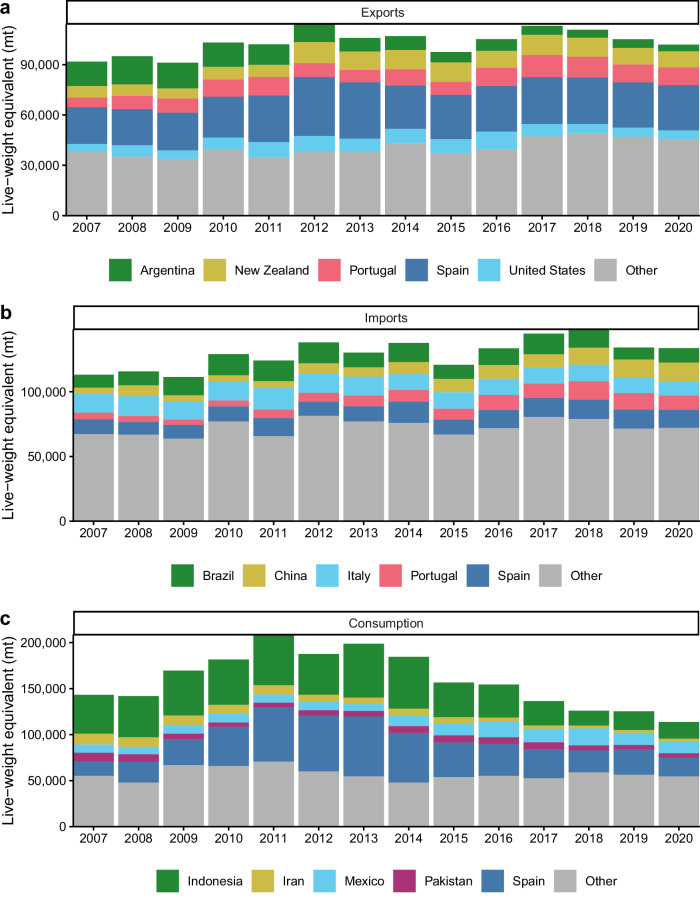
Trade volume by country. Shown is the estimated tonnage of (**a**) export, (**b)** import, and (**c)** domestic consumption of shark products by country. The top contributing countries (*n* = 20) are labeled and all others are grouped as “Other” (*n* = 74 for exports, *n* = 166 for imports, *n* = 80 for domestic consumption).

From 2007 to 2020, 94 countries landed and exported shark products. Exports were highly concentrated, with seven countries supplying 75% of all exported shark products (Table 1), although the top contributors varied annually (Fig. 2). Nearly twice as many countries (185) imported shark products between 2007 and 2020 and imports were more broadly distributed, with 15 countries accounting for 75% of all imported shark products (Table 1). Top importing countries varied annually (Fig. 2). Top importing and exporting countries were mostly distinct: only three top shark product exporters were also key importers (Spain, Portugal, and the United States; Table 1). Three of the top export countries, including Spain, Indonesia, and Portugal, also had high domestic consumption of shark products. Spain and Indonesia alone accounted for nearly half of the world’s domestic consumption of shark products (Table 1). Annual exports, imports, and domestic consumption of shark products under different finning regulations are shown in Supplementary Figs. 1–3 and annual statistics by country can be found in Supplementary Data 2.

**Table 1:** Top contributing countries in the global trade network

ARTIS data source	Country	% of grand total 2007–2020	Regulations adopted
Exports (*n* = 94)	Spain**	26.1%	FNA (2013)
	New Zealand	9.3%	FRU (1999), FNA (2014)
	Portugal**	9.3%	FNA (2013)
	Argentina	9.2%	FRU (2009)
	United States*	7.1%	FRU (2000), FNA (2011)
	Indonesia*	6.3%	FCR (2013)
	Ecuador	5.4%	FNA (2007)
Consumption (*n* = 100)	Indonesia*	24.9%	FCR (2013)
	Spain**	23.5%	FNA (2013)
	Mexico	7.3%	FRU (2007)
	Iran	4.4%	
	Pakistan	4.0%	
	France*	4.0%	FNA (2013)
	Portugal**	3.3%	FNA (2013)
	Taiwan*	3.2%	FNA (2012)
Imports (*n* = 185)	Italy	10.5%	FNA (2013)
	Brazil	10.1%	FCR (1999), FNA (2012)
	Spain**	10.0%	FNA (2013)
	China	6.9%	
	Portugal**	6.5%	FNA (2013)
	France*	5.2%	FNA (2013)
	United States*	4.6%	FRU (2000), FNA (2011)
	Japan	3.6%	FNA (2009)
	Uruguay	3.2%	
	Australia	2.8%	FRU (2000), FNA (2011)
	Taiwan*	2.5%	FNA (2012)
	Germany	2.4%	FNA (2013)
	South Korea	2.3%	
	Greece	1.9%	FNA (2013)
	United Kingdom	1.8%	FNA (2013)

Shown are the top contributing countries to the export, consumption, and import of shark products. Asterisks indicate whether the country is also a top contributor in one (*) or two (**) other categories. Regulations include fins naturally attached (FNA), fin-to-carcass ratio (FCR), and finning regulation unspecified (FRU).

### Impact of finning regulations on the volume of traded shark products

**Fig. 3:**
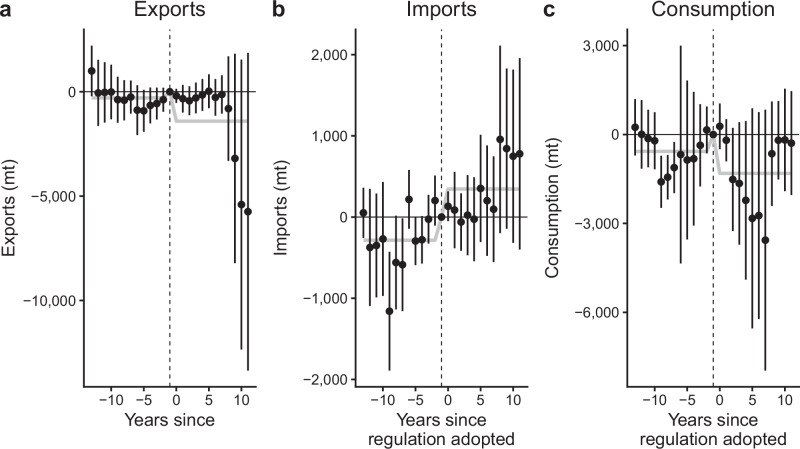
Trends in trade flows before and after regulation. Shown are difference in-difference event study plots by year for each trade outcome: (**a)** exports (*n* = 743 country-years), (**b)** imports (*n* = 785 country-years), and (**c)** domestic consumption (*n* = 827 country-years). The event study plot shows the estimated trends before any shark finning regulation is adopted (left of the dashed line at the reference year, year = −1) and dynamic treatment effects in each year following regulation adoption relative to the reference period (right of the dashed line). The study unit is a country-year where the treatment group includes country-years where a shark finning regulation is in place and the control group (*n* = 279) are country-years that have no shark finning regulations. Data are presented as the event-time coefficient (black dots) with 95% confidence intervals. The gray line is the aggregated treatment effect line, which is the mean of all difference estimates before (left of the dashed line) and after (right of the dashed line) regulation adoption.

We used a Difference-in-Difference (DiD) approach to evaluate whether shark-finning regulations implemented over time have caused concomitant changes in three trade outcomes for shark products: (1) exports, (2) imports, and (3) apparent domestic consumption (Supplementary Tables 3-4). DiD models compare trends before and after the adoption of finning regulations between regulated (treated) and unregulated (control) countries to determine the causal impact of regulation on each outcome. By including global and regional fixed effects, which absorb time-varying global or regional market forces such as demand growth or demographic change, the DiD models are able to isolate the change in trade and domestic consumption attributable to finning regulations from broader market forces. These models rely on the parallel trends assumption that without a regulation, treated and control countries would have followed the same trends in exporting, importing, and consuming shark products. As such, a DiD model does not directly evaluate trends over time (e.g., countries had higher exports before imposing a regulation), but rather evaluates the relative difference between control and treated countries at each time step (e.g., countries with regulations had lower exports compared to control countries in year 10). In our context, “treated” countries are those that adopted any finning regulation and “control” countries are those that have not yet adopted such regulation in a given year or never adopt such regulations (Supplementary Fig. 4). For each of the trade outcomes we evaluated, we found the parallel trends assumption appears plausible; prior to the adoption of a shark finning regulation, the estimated differences in trajectories between countries with regulations and those without are statistically indistinguishable from zero (i.e., all countries have similar trends in exports, imports, and domestic consumption of shark products before a regulation is implemented; Fig. 3).

Exports of shark products in countries with regulations appear relatively stable for the first seven years after adoption. After year seven, exports tend to decline in countries with regulations compared to those without, with steeper declines observed the longer regulations have been in place (Fig. 3). On average, countries with regulations export 351 mt per year less than they would have in the absence of regulation, with a maximum difference of 5746 mt at 11 years post-regulation. However, we find no strong statistical evidence that this trend in the volume of exported shark products by regulated countries is significant (ATT = –351 mt per year; 95% CI: −1093 to 392, *p* = 0.350). After the adoption of finning regulations, imports of shark products continue to fluctuate around pre-adoption levels, with a slight positive trend in years 8–11 (Fig. 3). On average, countries with regulations import 142 mt more per year than they would have if they did not have regulations in place. Similarly, we find no strong evidence that this change in imports to regulated countries is statistically significant (ATT = 142 mt per year; 95 % CI: –314 to 190; *p* = 0.538). Unlike exports and imports which remained relatively unchanged immediately following regulation, domestic consumption of shark products appears to decline over the first seven years after the adoption of a finning regulation, though this reduction is not sustained over longer time scales (Fig. 3). On average, countries with regulations consumed 1672 mt less shark product per year than they would have without a regulation in place but, again, we find no evidence that this change is statistically significant (ATT = 1672 mt per year; 95 % CI: -3623 to 280; *p* = 0.092).

We assessed the credibility of our findings through six sets of robustness checks, by (1) limiting the treatment group to only countries that adopted the strongest finning regulation (fins naturally attached), (2) limiting the control group to countries not yet regulated at the time, but which eventually adopt similar legislation, (3) limiting the treatment group to only countries that consistently land shark products, (4) stratifying the countries by baseline trade volumes of shark products, (5) differentiating species groups occupying different habitats (i.e., coastal, pelagic, deep-sea sharks), and (6) including additional controls for a countries’ population size and per-capita income. Consistency between the results of the primary model and robustness checks ensure that the reported outcomes are not driven by a specific finning regulation, countries that never implement a regulation, countries that do not consistently trade shark products, countries that contribute the most to the shark trade, species groups from a particular habitat, or by a country’s population and income. Detailed regression results are presented in the Supplementary Information (Supplementary Tables 5−10) and are consistent with our main results. Finally, we conducted a sensitivity analysis on each of the trade outcomes using a “leave-one-out” approach to determine the effect that individual countries had on the model results. This approach uses a diagnostic statistic called DFBETAS (see Supplementary Table 1) which is used to determine how much the treatment effect changes when each country is iteratively removed from the analysis. For each trade outcome we find the most influential countries exert moderate influence (0.5 < DFBETAS < 1) over the model results with Guinea (DFBETAS = 0.74), Uruguay (DFBETAS = 0.48), and Indonesia (DFBETAS = 0.98) being the most influential for exports, imports, and domestic consumption, respectively. Reported exports in Guinea declined to zero after the implementation of finning regulations, making it a high influence country for the DiD trends even though the volume of exports overall is not a statistical outlier. Uruguay and Indonesia are both top contributing countries to imports and domestic consumption, respectively. All leave-one-out estimates had the same sign and significance as the full-sample results indicating that influential countries affect the size of the coefficients but do not change overall trends, reinforcing confidence in the robustness of our findings (Supplementary Table 11).

### Key actors and relationships in the global shark trade network

**Fig. 4:**
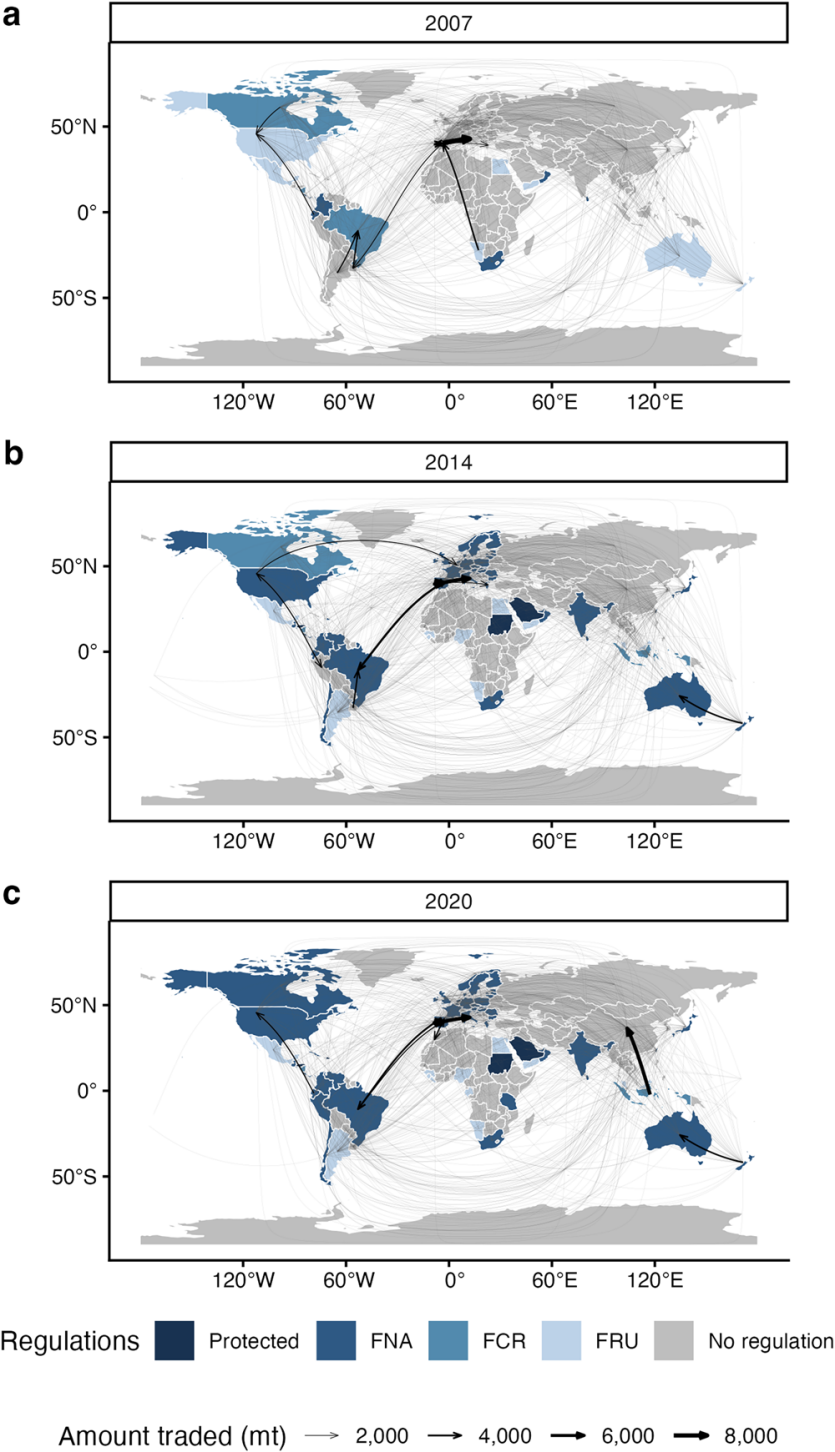
Global shark trade network. Shown are trade flows of shark products between all trading countries for selected years (**a**) before (2007), (**b)** during (2014), and (**c)** after (2020) most shark finning regulation came into force. Light, thin gray lines represent all trade flows and the darker, thicker lines represent the top 10 trade flows each year. Arrows designate the direction of flow, starting in the exporting country and pointing towards the importing country and are weighted by total amount traded (mt). Shark fishing and finning regulations that were already adopted in each year are colored according to regulation type, with darker colors representing stronger regulations (Protected - sharks are fully protected from fishing; FNA - fins naturally attached; FCR - fin to carcass ratio; FRU - unspecified finning regulations). The world basemap was pulled from the ‘rnaturalearth‘ R package^46^. Trade flows for all years can be found in Supplementary Fig. 11.

We used network analysis to explore patterns of connectivity between shark-trading nations. In each year, we evaluated the in- and out-degree strength and normalized in- and out-degree centrality of each country^22^ (see Supplementary Table 1 for full definitions). Annual network analysis (Supplementary Figs. 5–8) and annual statistics by country can be found in Supplementary Data 2. In-degree strength measures total imports to a country and reflects how dependent a country is on foreign goods. Normalized in-degree centrality is a relative measure of how well connected and central a country is within the network. It is measured as the number of trade partners a country imports from relative to all possible countries it could import from. Conversely, out-degree strength measures total exports from a country and reflects how much a country contributes to global trade. The normalized out-degree centrality is the relative importance of a country as an exporter within the network. It is measured as the number of trading partners a country exports to relative to all possible countries it could export to. In each year, we also calculated density and modularity for the global network as a whole to assess the density of trade connections and community structure (Supplementary Table 1). Finally, we assess a visual representation of the largest annual trade flows at three check-points within the dataset, before, during, and after most finning regulations were implemented: 2007, 2014, and 2020 (Fig. 4).

Italy and Spain were consistently among the countries with highest in-degree strength from 2007 to 2020, indicating sustained reliance on imported shark products over time. Brazil, China, and Portugal were also among the countries with highest in-degree strength in most years. Spain, France, Italy, Japan, and China were among the countries with highest in-degree centrality each year, indicating that they are the most well-connected importing countries. For example, Spain’s average in-degree centrality value was 0.22, meaning that, on average, they imported from 22% of all countries exporting shark products. Countries with both high in-degree strength and centrality, like Spain and China, are critical hubs in the trade network, importing high volumes of shark products from a large proportion of exporting countries (22% on average in both countries).

Spain, New Zealand, and Portugal consistently showed among the highest out-degree strength from 2007 to 2020, indicating they contribute the largest volumes of shark products to the global market. Other important exporting countries included the United States, Indonesia, and Argentina. Argentina, Spain, and New Zealand were among the countries with highest out-degree centrality in each year, indicating that they are the most well-connected exporting countries. Spain had the highest out-degree strength every year and among the highest out-degree centrality, showing high importance within the global network both in terms of volume and connectivity. On average, Spain exported to nearly half of all countries importing shark products.

As a whole, between 2007 and 2020, the density of the global shark trade network decreased slightly from 0.05 to 0.04 (Supplementary Fig. 9) indicating persistently sparse connectivity with fewer active country-to-country trade partnerships over time. At the same time, network modularity increased slightly from 0.38 in 2007 to 0.46 in 2020 (Supplementary Fig. 10) signifying a shift toward more regionally distinct trading clusters. A visual inspection of specific trade flows shows annual variation in both the total amounts traded among countries and the specific sources and destinations of major trade transactions consistent with the patterns in the network analysis (Fig. 4, Supplementary Fig. 11). For example, in 2007 most shark products flowed from Spain to Italy (9383 mt), followed by Portugal to Spain, and Spain to Portugal. These three trade flows remain the most important in 2014 and 2020, though we also see larger imports to Brazil in 2014 (from Spain and Uruguay) and China in 2020 (from Indonesia).

### Impact of finning regulations on shark trade network relationships

We applied the same DiD approach to the annual country-level network metrics to assess whether shark finning regulations implemented over time have caused concomitant changes in a country’s: (1) importing trade partnerships (in-degree centrality); and (2) exporting trade partnerships (out-degree centrality; Supplementary Tables 12−13). In-degree and out-degree strength are alternative ways to represent total imports and exports, respectively, and temporal trends and treatment effects mirror the trade outcome analysis of imports and exports (Supplementary Fig. 12, Supplementary Table 14). We performed the same set of six robustness checks for in-degree and out-degree centrality and found the results were consistent with those in the primary models (Supplementary Tables 15–20).

**Fig. 5:**
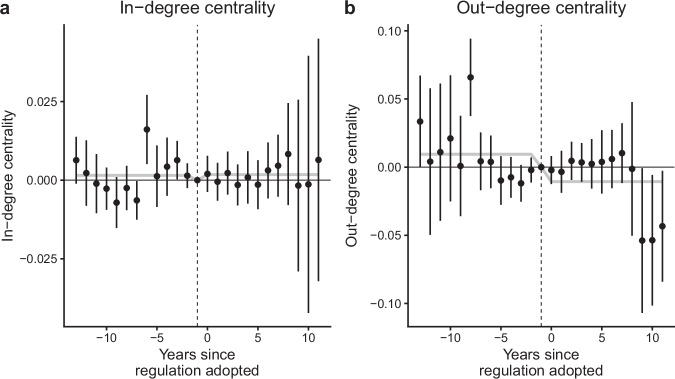
Trends in trade networks before and after regulation. Shown are difference-in-difference event study plots by year for (**a**) in-degree (*n* = 785 country-years) and (**b)** out-degree centrality (*n* = 743 country-years) network outcomes. The event study plot shows the estimated trends before any shark finning regulation is adopted (left of the dashed line at the reference year, year = −1) and dynamic treatment effects in each year following regulation adoption relative to the reference period (right of the dashed line). The study unit is a country-year where the treatment group includes country-years where a shark finning regulation is in place and the control group (*n* = 279) are country-years that have no shark finning regulations. Data are presented as the event-time coefficient (black dots) with 95% confidence intervals. The gray line is the aggregated treatment effect line, which is the mean of all difference estimates before (left of the dashed line) and after (right of the dashed line) regulation adoption.

Initially following regulation, in-degree centrality fluctuated around pre-adoption levels, indicating that importing trade partnerships remained relatively unchanged for some time (Fig. 5). In later years (8−11 years post-adoption), in-degree centrality increased, showing that countries with regulations traded with a higher proportion of exporting countries relative to countries without regulations. However, we find no statistical evidence that this change in import partnerships is significant (ATT = 0.001; 95% CI: 0.005–0.007, *p* = 0.663). Similarly, out-degree centrality showed minimal changes in the first seven years after regulations were adopted (Fig. 5), indicating that exporting trade partnerships were relatively unchanged for countries with regulations. However, 9-11 years post-adoption, we observed stronger declines in out-degree centrality, indicating countries with regulations exported to a smaller proportion of countries relative to countries without regulations. We also find no statistical evidence that this change in export partnerships is significant (ATT = 0.001; 95% CI: –0.013 to 0.015, *p* = 0.837). The “leave-one-out” approach identified Guinea and Taiwan as the most influential countries for in-degree centrality (DFBETAS = 0.56) and out-degree centrality (DFBETAS = 0.53), respectively, both with moderate influence over the results. In all cases, removing these influential countries does not change the sign or significance of the treatment effect ensuring that the overall result is robust (Supplementary Table 21).

## Discussion

This study isolates the effects of shark finning regulations on prevailing trade patterns for shark products at a global scale. From a visual assessment of the data, we note that in the years immediately following the adoption of finning regulations, there were declines in the volume of shark products consumed domestically but relatively minimal changes in exporting and importing trends in countries with regulations. Nine years or more after regulations were adopted, no sustained impacts were observed for domestic consumption, but countries with regulations exhibited greater declines in exports and increases in imports compared to countries without regulations. Similarly, we found that shark finning regulations had no immediate impact on trade network relations but, over the long-term (nine years or more post-adoption), countries with regulations exported to a smaller proportion of countries (decreasing out-degree centrality) and imported from a larger proportion of countries (increasing in-degree centrality). Taken together, in the long-term, we find countries with regulations are exporting a lower volume of shark products to a smaller proportion of countries and importing a higher volume from a larger proportion of countries, while domestic consumption remains relatively unchanged. These general trends are not consistent with the hypothesis that finning regulations could have a strong positive effect on trade or consumption.

The observed lack of statistical significance may also point towards some data limitations in our analysis. Importantly, while the ARTIS database represents progress toward higher resolution trade data, we could not isolate the effects of finning regulations on the trade in shark fins, which shows some signs of decline^8^. The ARTIS database tracks only primary products in order to avoid double counting within the mass balance approach^23^. Shark fins are considered a co-product and are therefore assigned a conversion factor of zero, essentially removing them from the model during the conversion from product weights to live-weights. More generally, poor taxonomic reporting of shark landings also remains a critical challenge. Since finning regulations apply to only true sharks (Selachii), saw- and wedgefishes (Rhinopristiformes), our analysis excluded any data reported as Elasmobranchii, where the volume of sharks versus skates and rays could not be distinguished. This precluded some countries that have been reported as key shark exporters (Japan and India) or consumers (India and Nigeria)^10^ from being analyzed. Additionally, for some countries that were included in our assessment, such as Taiwan and Brazil, only a portion of their trade data is included in the analyses, as large proportions of exports and domestic consumption, respectively, were reported as Elasmobranchii. Finally, domestic consumption is not formally reported and ARTIS relies on production data reported to the FAO to estimate apparent domestic consumption, assuming that whatever is domestically-sourced but not exported must be consumed locally^21^. Misreporting and under-reporting of sharks often occurs in national datasets and in local markets^19,20^, which could result in biased estimates of apparent domestic consumption.

Our results could also be influenced by the inherent qualities of countries that choose to implement shark finning regulations. For example, countries that choose to implement strong shark finning regulations may already manage their fisheries with sustainability or conservation objectives in mind (e.g., the Maldives have adopted several regulations to protect sharks and support dive tourism^24^) or may already play a small role in shark fishing globally. The implementation of a shark finning regulation is unlikely to change fishing behavior or impact trade if, for example, a country already lands whole sharks (e.g., retained targeted shark fisheries in Indonesia^25^) or does not regularly participate in shark fisheries (e.g., Lithuania). Further, even after regulations are adopted, most take several years to be implemented and enforced, which may result in a temporal lag between when finning regulations are adopted domestically, when fishing behavior actually changes, and, subsequently when shifts in trade patterns are detectable. We found stronger changes in imports and exports for countries that adopted shark finning regulations early in the time series, which allowed for more years of data to be evaluated after regulations were adopted.

Finally, we assessed the influence of shark finning regulations given their prevalence globally and broad applicability to all shark species; however, other regulations adopted during the assessed time period (2007–2020) may have stronger impacts on trade flows or domestic consumption than shark finning regulations alone. Regulations that control global trade of endangered species and their byproducts (i.e., Convention on International Trade in Endangered Species, CITES), or domestic trade regulations that prohibit imports or exports of shark products, may serve as primary driving factors in temporal trends of shark trade and domestic consumption. Throughout the time series, 42 shark species in the ARTIS database were listed under CITES and 29 countries that adopted shark finning regulations also adopted export bans on all shark products. While recent work finds that CITES species are still regularly traded several years after being listed for global protection^8,14,19^, the impacts of these regulations on global trade flows has not been assessed. The DiD approach used in this study requires the staggered adoption of a policy across countries over time. Since CITES listings operate at the global scale and all countries are affected at the same time when a species is listed, the DiD approach used here is not suitable. Evaluating the impact of CITES listings would require a different methodological approach and constitutes a promising direction for future research.

The trend towards long-term declines in exports observed in our model, paired with relatively unchanged domestic consumption, indicates that fewer whole sharks are being landed and supplied to markets from countries with finning regulations. This could be due to fewer shark interactions resulting in lower overall shark catch, though this is less consistent with evidence pointing to the continued rise in global shark mortality^7^. Alternatively, shark finning regulations may have disincentivized some countries from retaining sharks altogether, and catch that was previously finned is now being discarded. This trend may be influenced further by retention bans for threatened shark species that have been increasingly implemented in pelagic fisheries regulated by Regional Fisheries Management Organizations (RFMOs) and an observed reduction in pelagic shark mortality in these fisheries^7^. Interestingly, pelagic sharks were the only group in our habitat-specific analyses that showed a statistically significant decline in exports (Supplementary Table 9), which is consistent with decreased retention and increased discarding of incidental catch of these species. Discarded catch is not always reported or included in national statistics but can be associated with high at-vessel and post-release mortality for many species (e.g.^26–28^). Importantly, data on discarded shark catch are not included in the ARTIS database and we did not quantify discard rates or directly test whether finning regulations have caused higher discard rates for incidentally captured sharks. Future research could further investigate the landscape of discarded shark catch and potential regulatory actions to reduce associated mortality.

The other long-term trends observed in this study, where regulating countries export smaller volumes and import greater volumes of shark products, mimics historical trade trends observed for other aquatic species in which high-income countries reduce their exports and instead, increase imports of high-valued seafood from low-income countries^21,29–31^. In addition, fisheries management can unintentionally displace fishing effort to less regulated foreign waters or the high seas^32^. Together, these types of trade dynamics can lead to healthier stocks where regulations are implemented at the expense of stocks that are increasingly targeted in areas with little to no regulation, though this connection has been under-researched for marine fisheries given challenges relating trade data to catch location. Whether these observed trade patterns have, or could, lead to the overexploitation of shark populations in poorly managed countries is an important avenue for future research.

This study serves as a stepping stone towards a better understanding of how regulatory measures, like finning regulations, interact with and influence global trade flows and networks. Our analysis supports previous work showing that shark trade is dominated by a few key nations such as Spain, Italy, China, Indonesia, Brazil, and Argentina^10,14,17^. We also show that both global trade flows of shark meat (i.e., exports, imports, and domestic consumption) and trade networks (i.e., the proportion of trade partnerships) showed no statistically significant change with the imposition of shark finning regulations, on average. The methodologies used here might also be more broadly applicable. ARTIS is a publicly accessible database that tracks the global flow of species/species group capture and aquaculture products, starting from the point of production, through global trade networks, and ending in consumption. These data enable an understanding of how management has affected trade and yield insights that are critical to uncovering underlying economic drivers of mortality, both for sharks and possibly other vulnerable marine species that are internationally traded. By uncovering the linkages in capture and aquaculture supply chains, the ARTIS database provides an opportunity to understand what incentivizes the targeting of vulnerable marine species in the first place. The analytical framework presented here can be applied to understand the influence of other regulations on shark trade as well as the trade of any wildlife or plant-based commodity that is subject to changing regulatory regimes, providing potentially new insights into a major driver of global biodiversity loss^33^.

## Methods

### Shark finning regulation data

We leveraged the national shark finning and fishing regulations dataset developed in Worm et al.^7^ as a starting point for our analyses. This global dataset provided an annual record of shark finning and fishing regulations adopted by a country. For each of the 73 countries with a finning or fishing regulation, we constructed a regulatory time series from 1980 to 2022, where we denote the regulation present in each year. We assumed that shark finning regulations apply to all vessels from the regulated country regardless of where fishing activity occurred. We also re-evaluated each management measure to denote whether the participating country has any export bans for shark products. Regulations are categorized by strongest to weakest regulation as: (1) shark sanctuaries where all shark fishing is prohibited; (2) fins naturally attached (FNA), where all landed sharks must be whole with their fins attached; (3) fins artificially attached (FAA), where caught sharks may have their fins removed for storage, but re-attached via ropes or wires prior to landing; (4) fin-to-carcass ratio (FCR), where shark fins must be landed alongside shark carcasses in a defined ratio; or (5) unspecified finning regulations where there is some form of prohibition but its definition is not clearly documented^7^. For eight countries where multiple regulations were adopted in the same year, we retained only the strongest regulation. While we noted FAA regulations in our database, the two countries that actually adopted FAA (Taiwan and New Zealand) simultaneously adopted FNA. Therefore, no countries in our final database were defined as solely implementing FAA in any given year. Countries that have never adopted a finning regulation were excluded from this database.

### Aquatic resource trade in species database

We used the Aquatic Resource Trade in Species (ARTIS) database as our primary source of trade flows of shark products^21,23^. The ARTIS trade data included over 2400 species/species groups, 193 countries, and over 35 million bilateral trade records from 1996-2020. For each country, ARTIS modeled (1) the conversion of wild and farmed production into traded commodities, (2) proportion of imported commodities that were processed and exported, and (3) the apparent domestic consumption of each commodity. These estimates were then used to estimate each country’s exports sourced from domestic production versus imports and link re-exported products to their original product form and source country based on the mass-balance solutions and bilateral trade data. Once producing countries were estimated for all trade flows, the producing country’s species mix within each originally exported code was applied to disaggregate trade into species/species groups. Additional details of the methodology and mass balance calculations are provided in Gephart et al. (2024)^21^.

To avoid double counting in the mass-balance problem, only primary products were tracked (e.g., shark meat) while co-products (e.g., shark fins) were assigned a conversion weight of zero. The effect of this is that within the ARTIS trade data, only primary products appear and they are assigned the full live weight equivalent. Product information is reported under 6-digit harmonized system (HS) codes, which are administered by the World Customs Organization and updated every five years. ARTIS included five HS code versions (HS96, HS02, HS07, HS12, and HS17) and re-estimated the trade network for each HS version from the year of implementation through 2020 (e.g., 1996-2020, 2002-2020, etc.). Notably, new HS codes for specific shark products (i.e., shark fins, shark meat) were implemented in 2017. However, our analysis required a time series of trade data before and after regulations were adopted. Given many regulations were adopted in 2012, we used the 2007 HS codes and truncated our analysis to 2007–2020. Due to these data limitations, we were not able to separate the impact of finning regulations on the trade of shark meat versus shark fins specifically.

ARTIS trade records include the source country, importing country, exporting country, species, HS code (i.e., product code), production method (capture or aquaculture), export source (domestically-sourced exports or foreign-sourced re-exports), and the live-weight equivalent. We associated all production of shark products with the regulation adopted by the source country. Since production data are generally reported by the flag of the vessel irrespective of where fishing occurs and ARTIS is disaggregated to the producing nation, it lacks spatial resolution on where shark catch occurred making it impossible to determine if vessels were subject to additional stronger regulations than those of their flag country (e.g., a vessel from an unregulated country fishing within an area managed by a Regional Fisheries Management Organization with a finning regulation in place). We filtered trade records to include only true sharks. That is, excluding rays and any products reported simply as “elasmobranchii”. For each country and year, we calculated the total live-weight equivalents of: (1) exports; (2) apparent domestic consumption; and (3) imports. Imports included shark products that were directly exported from a producing country and products that were re-exported via intermediary processing countries (see Supplementary Table 1 for full definitions). Shark products originally sourced in the importing country that were exported for processing and then re-imported were excluded.

### Difference-in-difference analysis

We evaluated the causal effects of shark finning regulations on three trade outcomes (exports, imports, and domestic consumption) using a staggered difference-in-differences (DiD) design^34^. This design exploits the fact that countries adopted finning regulations (treatment) at different points in time, thereby creating “natural experiments”. The change in shark product trade and domestic consumption in each regulating country (treated countries) before and after implementation is compared to the change in a control group of countries (controls) that had not yet or never adopted the regulation.

DiD is common in policy evaluation because it helps to isolate the effect of a specific regulation from other global or country-level changes. By including global and regional fixed effects, the DiD models isolate the change in trade and domestic consumption of shark products attributable to finning regulations from broader market forces. Under the assumptions that (1) treated and control countries would have followed parallel trends absent regulation and (2) regulation adoption only impacts the adopting country, any systematic deviation in the outcome after adoption can be interpreted as the causal effect of the policy. The parallel trend assumption was visually assessed using an event study plot which shows how the trends in the treatment and control groups evolve over time. Small absolute values indicate small differences between the two groups satisfying the parallel trend assumption, while larger absolute values indicate large differences that do not satisfy the assumption.

Our analysis covered all countries landing shark products that were active in global trade between 2007 and 2020. We defined treatment timing *T*_*i*_ as the first year in which country *i* adopted any finning regulation (fins naturally attached, fin-to-carcass ratio, or unspecified finning regulations). At least two years of data before the adoption of a regulation is required to ensure credible estimate of the pre-treatment trend^35^. Therefore, we restricted the sample of the treated group to countries that adopted their first regulation in 2009 or later. Countries that implemented shark sanctuaries (complete fishing bans) at any point before or during the study period were excluded because these represent qualitatively different interventions.

To capture dynamic treatment effects over time, we estimated the event-study regression for each outcome *y*_*i*_ following the heterogeneity-robust staggered DiD framework outlined by Sun and Abraham (2021)^34^:

1 $${y}_{{it}}=\,\sum _{r\,\ne 1}{\beta }_{r}1\left\{t-{T}_{i}=r\right\}+\,{\alpha }_{i}+\,{\lambda }_{t}+\,{\gamma }_{1}\,{{wb}{\rm{\_}}{index}}_{{it}}+\,{\gamma }_{2}\left(\,{w{b}_{{region}}}_{{it}}\,\times \,t\right)+\,{\varepsilon }_{{it}}$$

Here, 1{*t* − *T*_*i*_ = *r*} is an indicator for being *r* years away from regulation (omitting *r* =* −1* as the reference year) and *α*_*i*_ and *λ*_*t*_ are country and year fixed effects that absorb time-invariant differences and common shocks, respectively. The co-variate, wb_index_*i,t*_, is the mean World Bank governance index on Voice and Accountability for each country from 2017 to 2019, and wb_region_*i,t*_ ×*t* allows each geographic region its own linear trend. *ε*_*i,t*_ is the idiosyncratic error term. Countries with greater Voice and Accountability values are perceived to have greater degrees of citizen participation in governance, which may make implementing and enforcing regulations more effective. Including the World Bank governance index in the DiD allows us to ensure our results were not only driven by inherent differences in governance. The coefficient of interest, *β*_*r*_, traces the average treatment effect at *r* years before or after adoption, which is a weighted average across cohorts that is robust to heterogeneity in adoption time and cohort-specific effects. We report standard errors clustered at the country level.

The primary result of this analysis is the average-treatment-on-the-treated (ATT) estimate. The ATT summarizes the overall average change in the outcome for treated countries relative to control countries. In total, the analyses included 743 treated country-years for exports, 827 treated country-years for domestic consumption, and 785 treated country-years for imports. All analyses were compared against the control group, which consisted of 279 country-years.

We assessed the influence of individual countries on the estimated treatment effect using DFBETAS, which measures how much the ATT changes when one country is removed, expressed in standard deviation units:

2 $${{DFBETAS}}_{i}=\,\frac{{{DFBETA}}_{i}}{{SE}(A\hat{T}{T}_{-i})}=\frac{A\hat{T}T-A\hat{T}{T}_{-i}}{{SE}(A\hat{T}{T}_{-i})}$$

A higher absolute DFBETAS indicates greater influence; generally values above 0.2 have the potential to meaningfully alter the results if excluded^36^. DFBETAS was used to identify countries that had the greatest influence over the model results. To evaluate sensitivity, we performed a “leave-one-out” analysis, which iteratively removed one country and recalculated the ATT. We then compare the sign and significance of these estimates to the full model to determine whether influential countries affected the overall conclusions.

### Network analysis

To understand whether differences in trade flows occurred after shark finning regulations were adopted, we ran a directed, weighted network analysis for each year of data. Network analyses have frequently been used in previous research analyzing global trade flows^37–39^. In our network analysis, each country was modeled as a node, and the trade transaction (the live-weight equivalent of shark products traded from one country to another) was modeled as an edge. Since the trade between countries is not always reciprocal, the network was modeled as a directed network, meaning the direction of trade flows (e.g., from A to B) is accounted for. Edges were weighted by the volume of shark products traded from one country to the other. For each node, in each year, we calculated in-degree and out-degree strength and in-degree and out-degree centrality (see Supplementary Table 1).

In-degree strength quantifies the total weight of all incoming edges to a node and out-degree strength quantifies the total weight of all outgoing edges from a node^22^. Formally, the in-degree ${s}_{i}^{{in}}$ and out-degree ${s}_{i}^{{out}}$ strength of country *i* are defined by Newman (2004)^40^:

3 $${s}_{i}^{{in}}=\,\sum _{j\,\in N}{w}_{{ji}}$$

4 $${s}_{i}^{{out}}=\,\sum _{j\,\in N}{w}_{{ij}}$$

where *w*_*ji*_ denotes the weight of the incoming edge representing the trade flow from country *j* to country *i*, *w*_*ij*_ denotes the weight of the outgoing edge representing the trade flow from country *i* to country *j*, and *N* is the set of all countries in the network.

In-degree and out-degree centrality captures the number of trade partners a country imports from or exports to, respectively. The in-degree and out-degree centrality estimates are normalized to enable comparison between the measures. The normalized values reflect the proportion of trading partners a country has relative to the total amount of partners they could have^22^. Formally, the in-degree ${C}_{i}^{{in}}$ and out-degree ${C}_{i}^{{out}}$ centrality of country *i* are defined by Opsahl et al. (2010)^41^:

5 $${C}_{i}^{{in}}=\,\frac{{k}_{i}^{{in}}}{n-1}$$

6 $${C}_{i}^{{out}}=\,\frac{{k}_{i}^{{out}}}{n-1}$$

where *k*_*i*_^in^ and *k*_*i*_^out^ are the number of incoming edges to country *i* and outgoing edges from country *i*, respectively, and *n* is the total number of countries in the network. ${k}_{i}^{{in}}$ and ${k}_{i}^{{out}}$ are further defined by Opsahl et al. (2010)^41^:

7 $${k}_{i}^{{in}}=\,\sum _{j\,\in N}{A}_{{ji}}$$

8 $${k}_{i}^{{out}}=\,\sum _{j\,\in N}{A}_{{ij}}$$

where A is an adjacency indicator, *A*_*ji*_ is one if an incoming edge from country *j* to country *i* exists and zero otherwise, while *A*_*ij*_ is one if an outgoing edge from country *i* to country *j* exists and is zero otherwise, and *N* is the set of all countries in the network. Normalized values of ${C}_{i}^{{in}}$ and ${C}_{i}^{{out}}$ range from 0 to 1, where zero means country *i* does not import from or export to any other countries, respectively, and one means country *i* imports from or exports to all possible countries, respectively.

Density and modularity were calculated each year for the network as a whole. Density measures the overall connectivity of a network by calculating how many trade partnerships between countries exist compared to how many could possibly exist. Density was calculated by Wasserman and Faust (1994)^42^:

9 $$D=\,\frac{m}{n(n-1)}$$

where *m* is the total number of edges in the graph and *n* is the number of countries, *n* − *1* represents the maximum number of countries that can be connected to any single country.

Modularity measures how well a network can be divided into clusters by quantifying the density of connections within a cluster and between clusters, relative to a random network^22^. Modularity values range from –1 to 1, with higher scores indicating modular networks with regionally driven trade patterns and lower scores representing less modular networks that are highly globally dispersed. Since modularity is concerned with understanding connections for all trade (both imported and exported), an undirected version of the trade network was used. The modularity score *Q* was calculated by Newman (2004)^40^:

10 $$Q=\,\frac{1}{2m}\,\sum _{i,\,j}\left[{w}_{{ij}}-\,\frac{{s}_{i}{s}_{j}}{2m}\,\right]\delta ({c}_{i}\,,\,{c}_{j})$$

where *w*_*i,j*_ is the weight of the edge between countries *i* and *j*, *s*_*i*_ and *s*_*j*_ is the sum of all weights of the edges connected to countries *i* and *j*, *c*_*i*_ and *c*_*j*_ is the community to which countries *i* and *j* are assigned, the *δ*(*u,u*) function is one when countries *i* and *j* are in the same community (*c*_*i*_=*c*_*j*_) and zero otherwise and *m* is the total number of edges in the network. Community structure was optimized using the Louvain algorithm^43^ and all network metrics were calculated using the igraph package^44,45^.

The same staggered DiD design described above was applied to evaluate the impact of shark finning regulations on four network outcomes: in-degree strength, out-degree strength, in-degree centrality, and out-degree centrality. In total, the analyses included 785 treated country-years for in-degree strength and in-degree centrality and 743 treated country-years for out-degree strength and out-degree centrality. All analyses were compared against the control group, which consisted of 279 country-years.

### Reporting summary

Further information on research design is available in the Nature Portfolio Reporting Summary linked to this article.

## Supplementary information

Supplementary Information

Description of Additional Supplementary Files

Supplementary Data 1

Supplemental Data 2

Reporting Summary

Transparent Peer Review file

## Data Availability

The trade data used in this study are from the Aquatic Resource Trade in Species database, available at 10.5063/F1ZS2V05. The World Bank Governance Indicators used in this study are the 2024 update, available from www.govindicators.org. The population and income data used in the study are from the 2024 update of the World Development Indicators, available from https://data.worldbank.org/indicator/SP.POP.TOTL and https://data.worldbank.org/indicator/NY.GDP.PCAP.PP.KD, respectively. Secondary income data used in this study are from the Penn World Table Version 11, available from http://www.ggdc.net/pwt. Additional source datasets developed for this analysis are included in the Zenodo code repository available at 10.5281/zenodo.15801243. Additional data that were generated in this study and used to create the figures are provided in the Supplementary Data files.

## Code availability

All code developed and used in this study is available for download and archived on Zenodo under 10.5281/zenodo.15801243.
