# Supplementary material for: Global trade responses to shark finning regulations
Source: Nat Commun. 2026 Jul 31;17:6821. doi: 10.1038/s41467-026-75625-1 (PMC13427823; doi:10.1038/s41467-026-75625-1)
Supplement: Supplementary file 1 — Supplementary Information [file 41467_2026_75625_MOESM1_ESM.pdf]

Supplementary Information

Global trade responses to shark finning regulations

Echelle S. Burns, Sara Orofino, Kaiwen Wang, Darcy Bradley, Nidhi G. D'Costa, Leonardo Manir Feitosa, Laurenne Schiller, Boris Worm, Jessica A. Gephart, Gavin G. McDonald

This PDF includes the following:

Supplementary Figures 1-12

Supplementary Tables 1-21

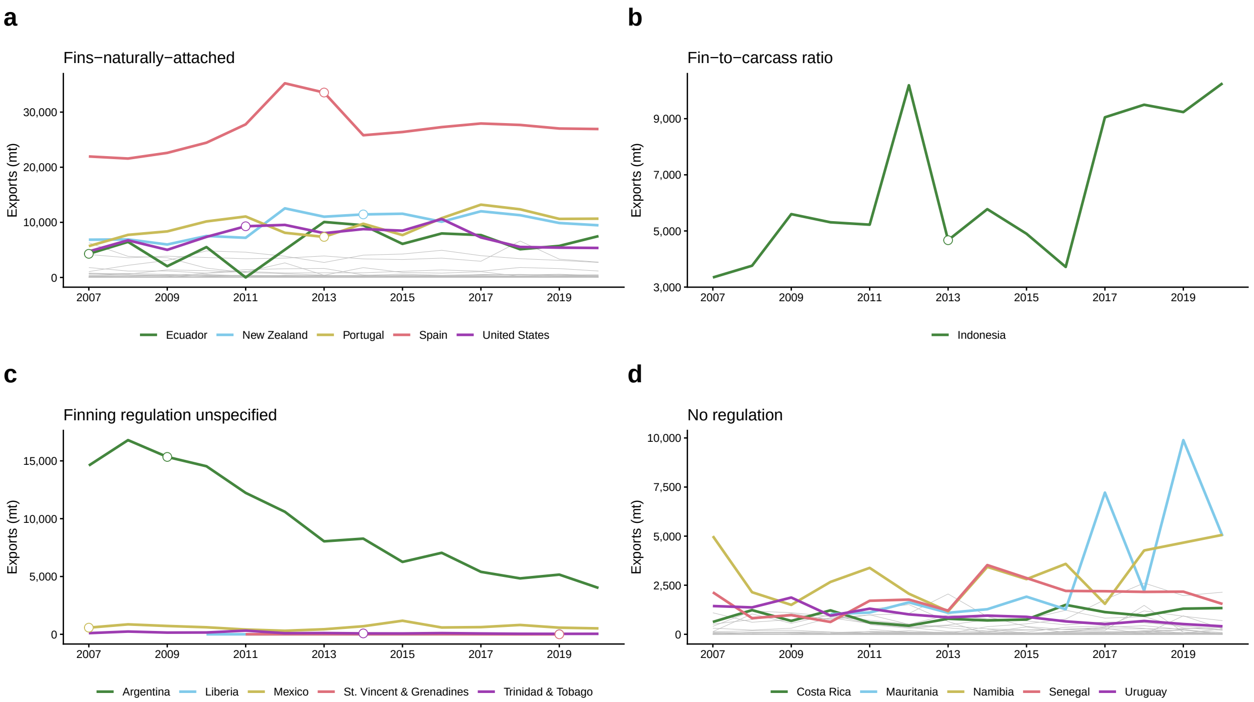

Supplementary Fig. 1: Country-level exports by regulation. Time series of country-level exports of shark products (metric tons, live-weight equivalent) under fins-naturally attached (a), fin-to-carcass ratio (b), unspecified finning regulations (c) and no regulations (d). The top 5 contributing countries for each type of regulation are colored, and other countries are depicted as gray lines. For subplots in which regulations are implemented, open circles for colored lines indicate the year in which the regulation was implemented.

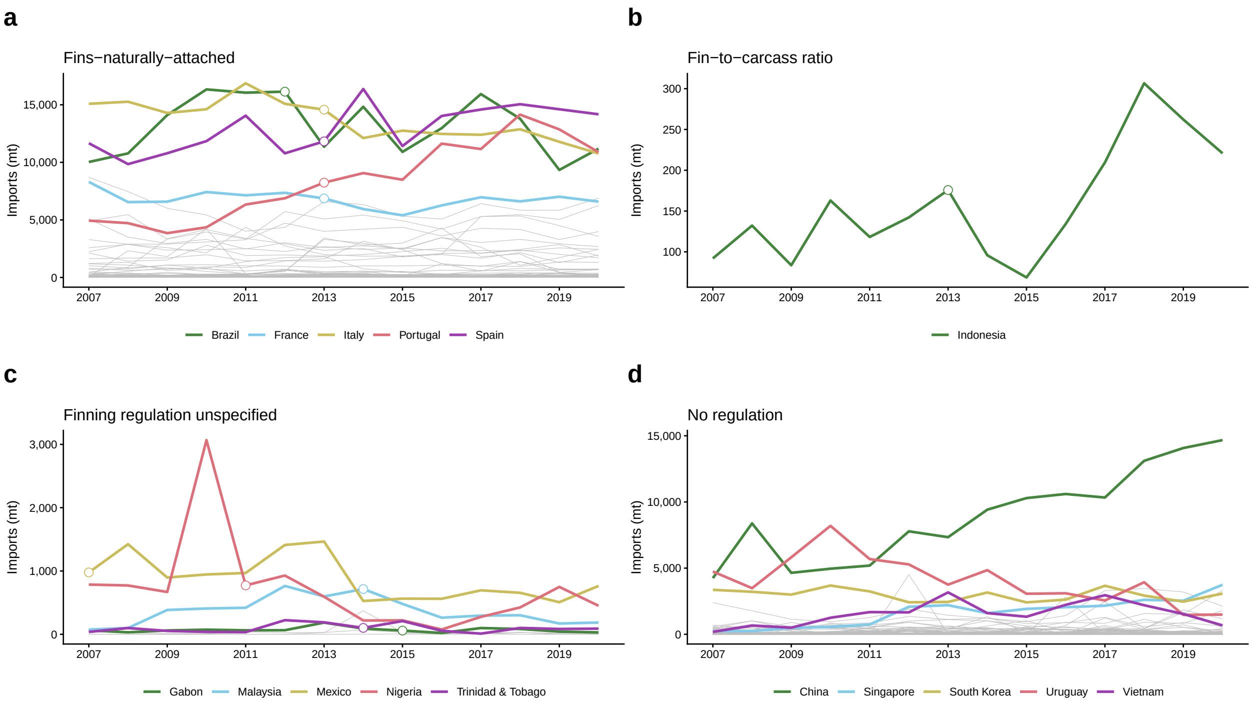

Supplementary Fig. 2: Country-level imports by regulation. Time series of country-level imports of shark products (metric tons, live-weight equivalent) under fins-naturally attached (a), fin-to-carcass ratio (b), unspecified finning regulations (c) and no regulations (d). The top 5 contributing countries for each type of regulation are colored, and other countries are depicted as gray lines. For subplots in which regulations are implemented, open circles for colored lines indicate the year in which the regulation was implemented.

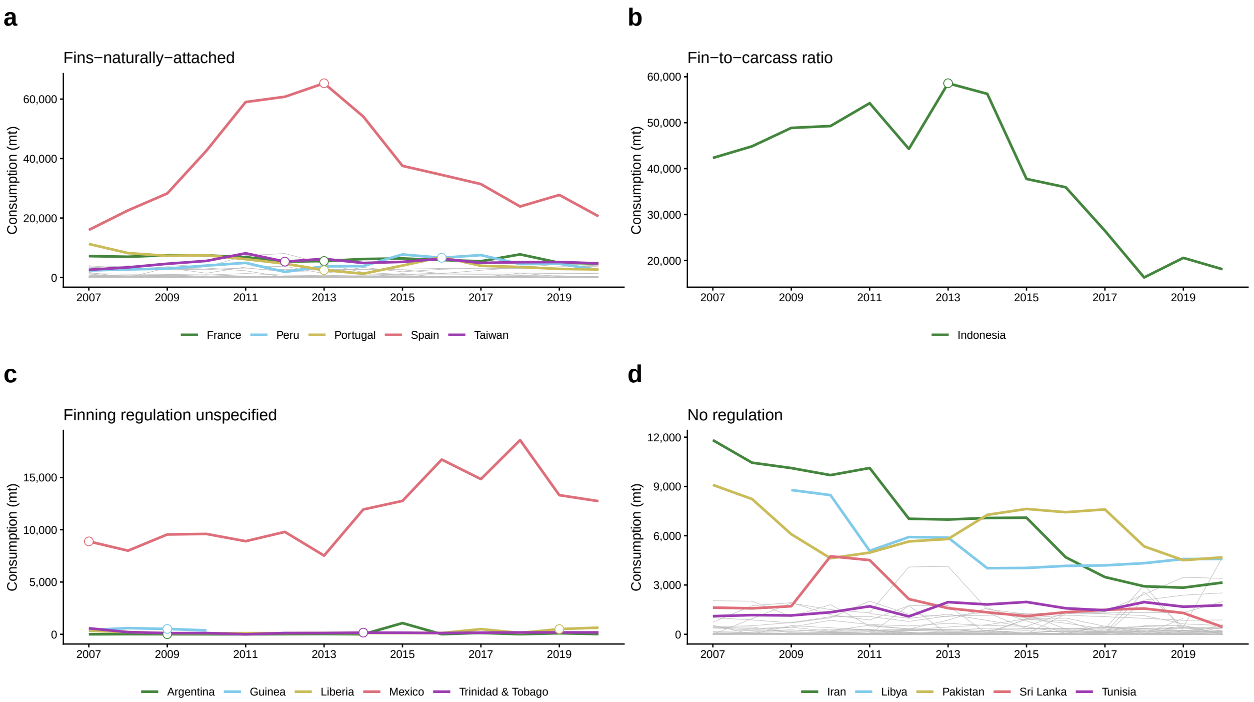

Supplementary Fig. 3: Country-level domestic consumption by regulation. Time series of country-level domestic consumption of shark products (metric tons, live-weight equivalent) under fins-naturally attached (a), fin-to-carcass ratio (b), unspecified finning regulations (c) and no regulations (d). The top 5 contributing countries for each type of regulation are colored, and other countries are depicted as gray lines. For subplots in which regulations are implemented, open circles for colored lines indicate the year in which the regulation was implemented.

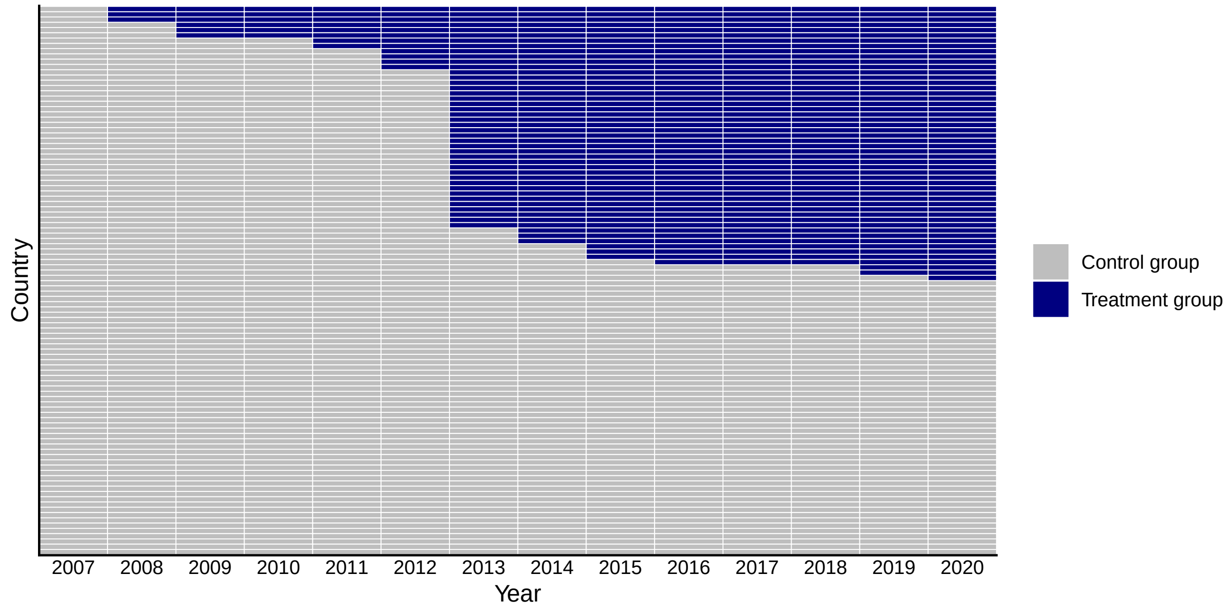

Supplementary Fig. 4: Treatment and control groups. The annual distribution of control and treatment groups for each difference-in-difference analysis. The control group (gray) consists of countries that are "never treated" (i.e., never implement a finning regulation) or are "not yet treated" (i.e., have not yet adopted a finning regulation). The treatment group (dark blue) consists of countries that have already adopted a shark finning regulation in that year.

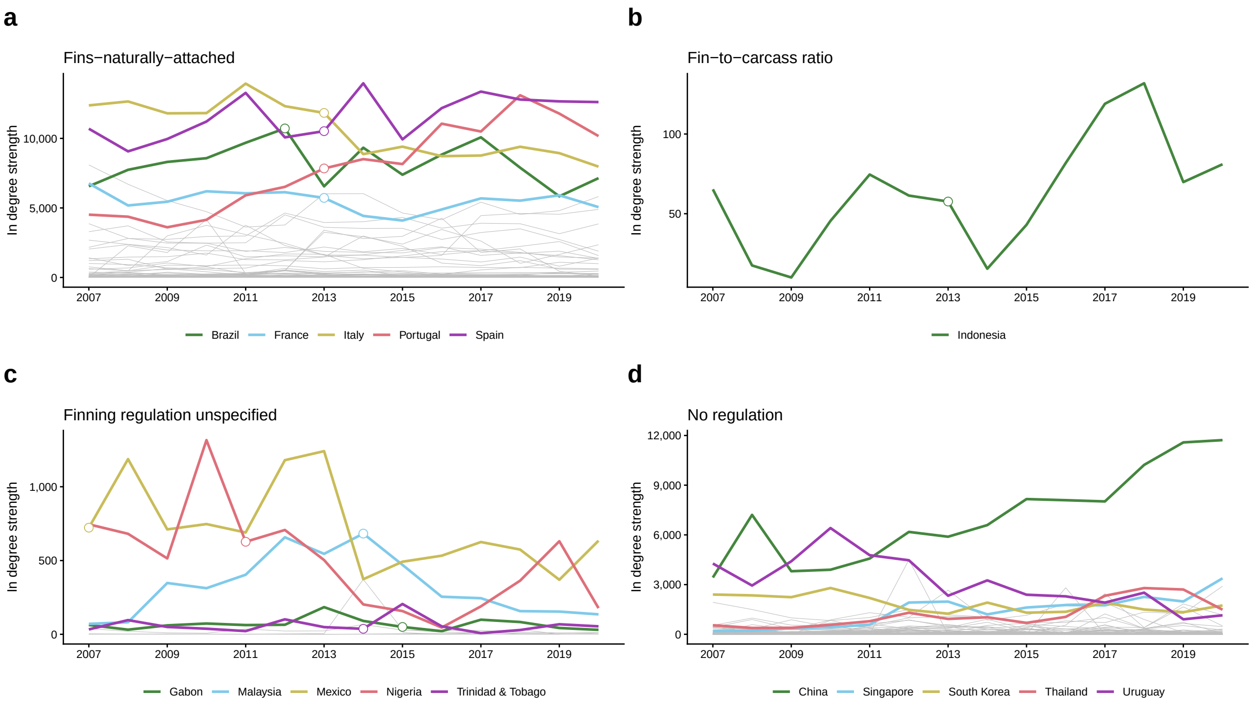

Supplementary Fig. 5: Country-level in-degree strength by regulation. Time series of country-level in-degree strength under fins-naturally attached (a), fin-to-carcass ratio (b), unspecified finning regulations (c) and no regulations (d). The top 5 contributing countries for each type of regulation are colored, and other countries are depicted as gray lines. For subplots in which regulations are implemented, open circles for colored lines indicate the year in which the regulation was implemented.

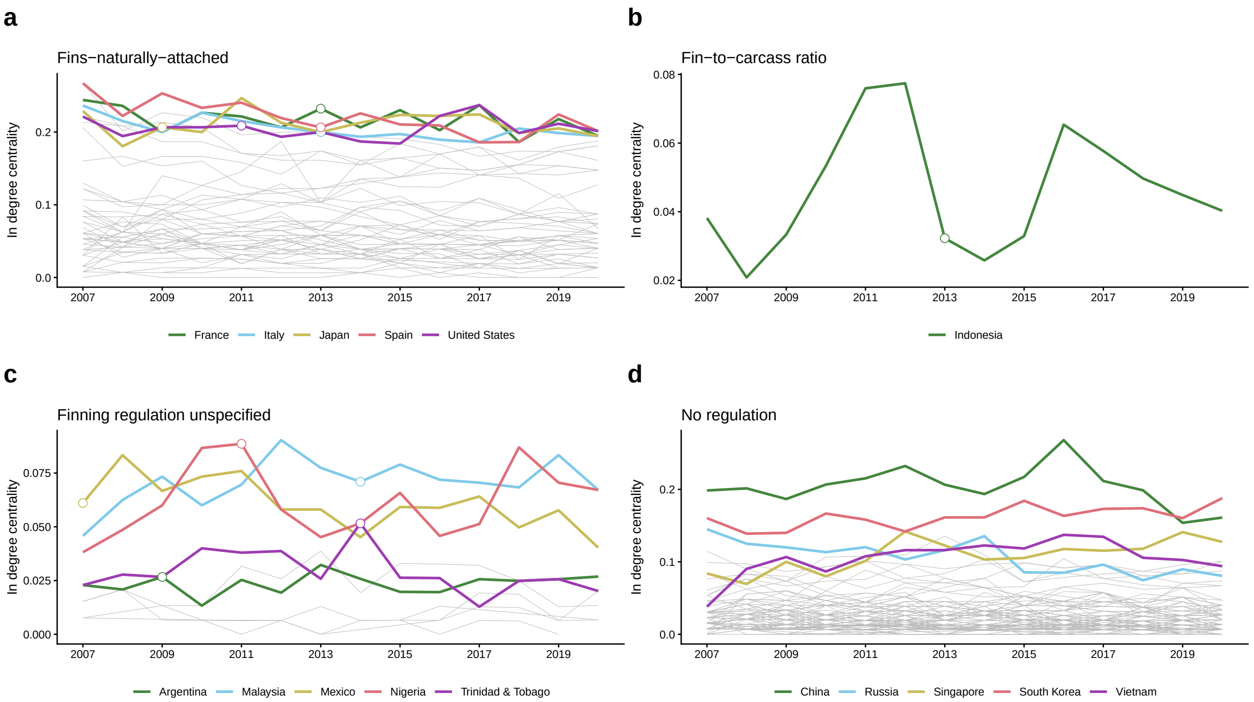

Supplementary Fig. 6: Country-level in-degree centrality by regulation. Time series of country-level in-degree centrality under fins-naturally attached (a), fin-to-carcass ratio (b), unspecified finning regulations (c) and no regulations (d). The top 5 contributing countries for each type of regulation are colored, and other countries are depicted as gray lines. For subplots in which regulations are implemented, open circles for colored lines indicate the year in which the regulation was implemented.

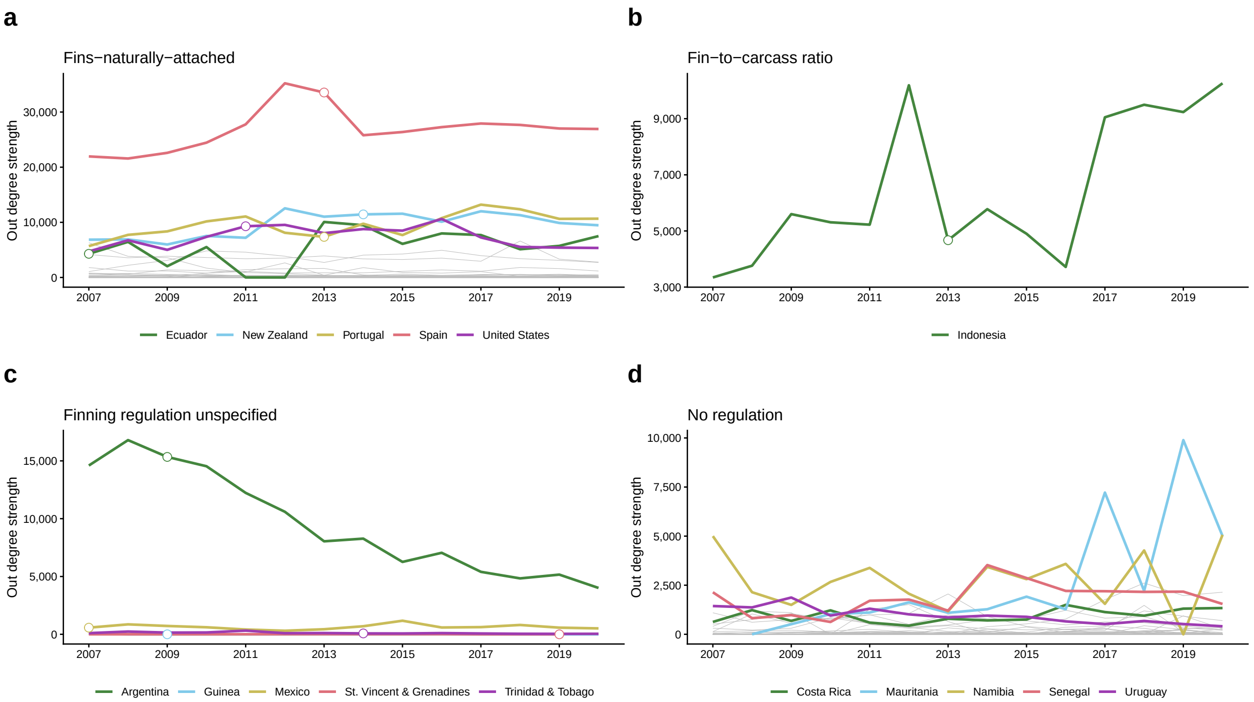

Supplementary Fig. 7: Country-level out-degree strength by regulation. Time series of country-level out-degree strength under fins-naturally attached (a), fin-to-carcass ratio (b), unspecified finning regulations (c) and no regulations (d). The top 5 contributing countries for each type of regulation are colored, and other countries are depicted as gray lines. For subplots in which regulations are implemented, open circles for colored lines indicate the year in which the regulation was implemented.

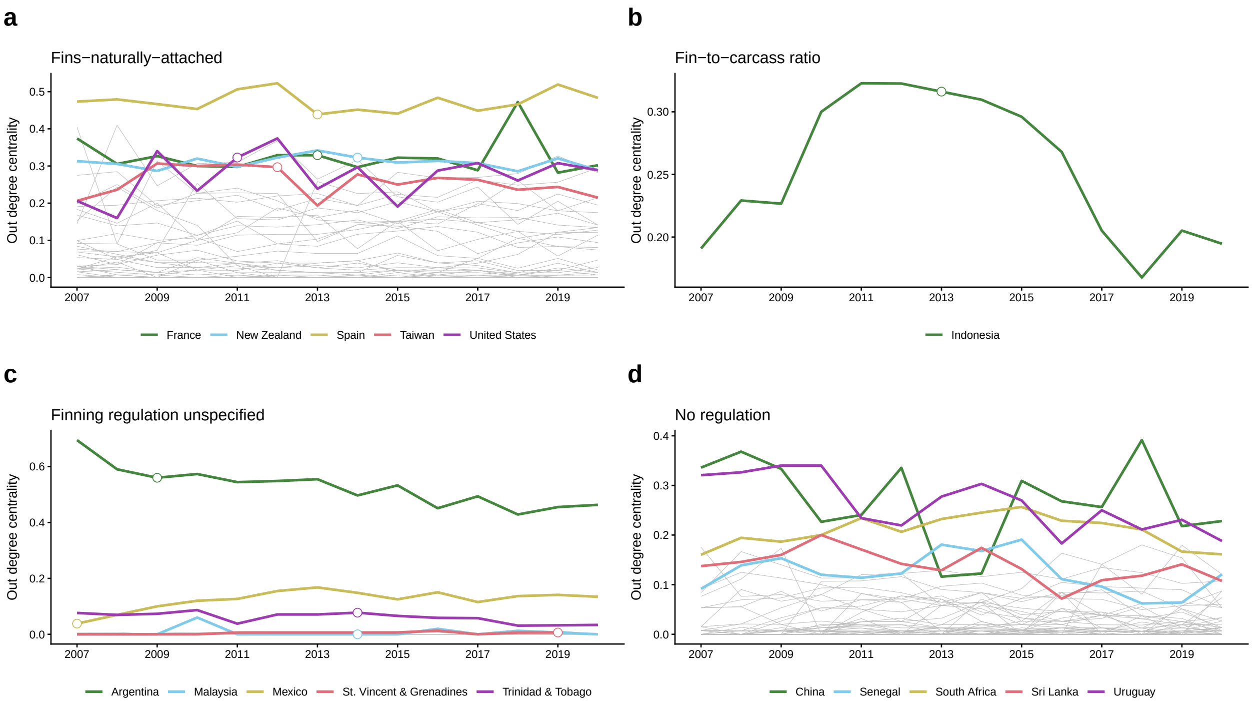

Supplementary Fig. 8: Country-level out-degree centrality by regulation. Time series of country-level out-degree centrality under fins-naturally attached (a), fin-to-carcass ratio (b), unspecified finning regulations (c) and no regulations (d). The top 5 contributing countries for each type of regulation are colored, and other countries are depicted as gray lines. For subplots in which regulations are implemented, open circles for colored lines indicate the year in which the regulation was implemented.

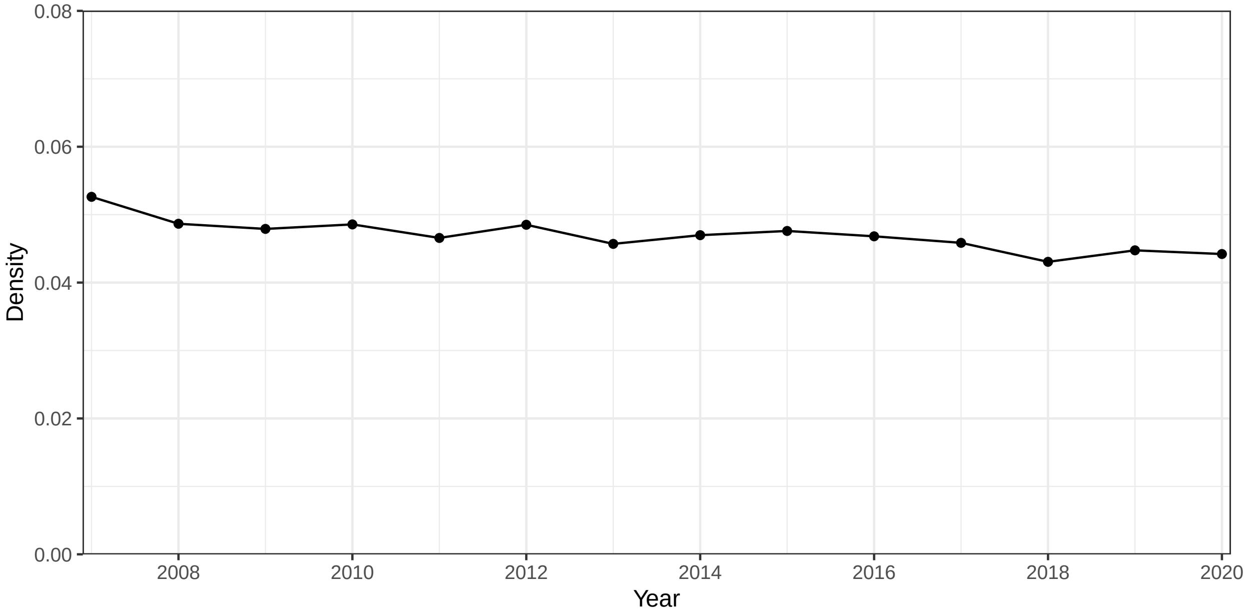

Supplementary Fig. 9: Network density. Time series of network density over the study period. Density is calculated for the network as a whole and reflects the number of active country-to-country trade partnerships relative to all possible trade partnerships. See Supplementary Table 1 for full definitions.

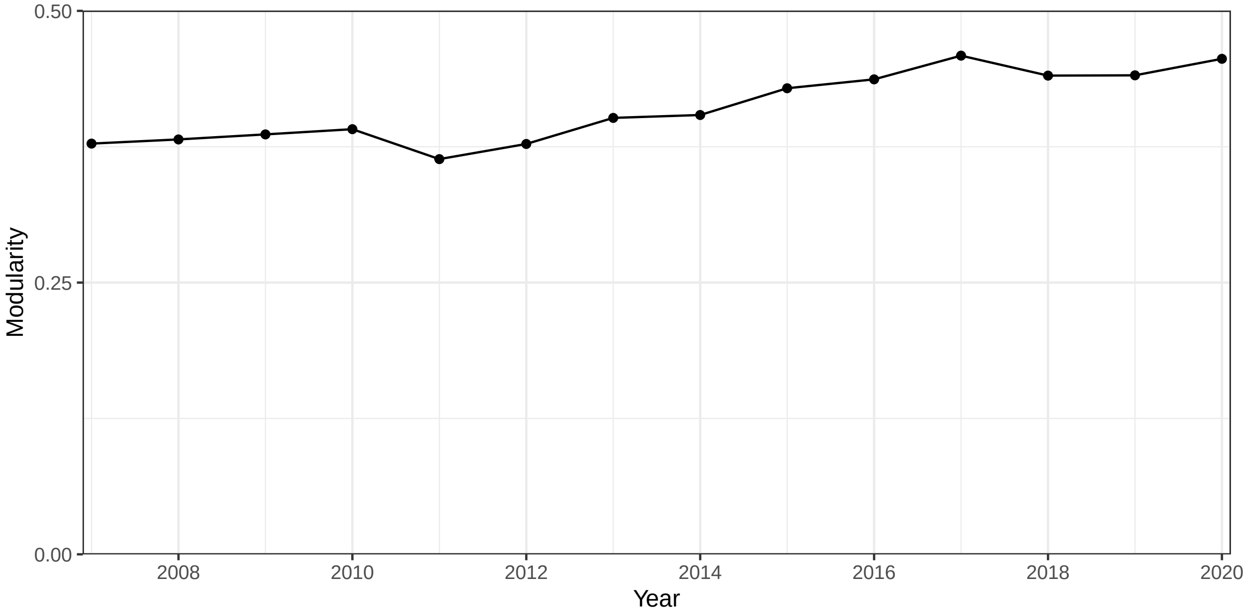

Supplementary Fig. 10: Network modularity. Time series of network modularity over the study period. Modularity is calculated for an undirected version of the network that considers total volume of shark products traded between countries but does not distinguish between imports and exports. Modularity values can range from -1 to 1, with high modularity signifying regionally clustered trade partnerships and low modularity representing a diverse global network with few distinct groups. See Supplementary Table 1 for full definitions.

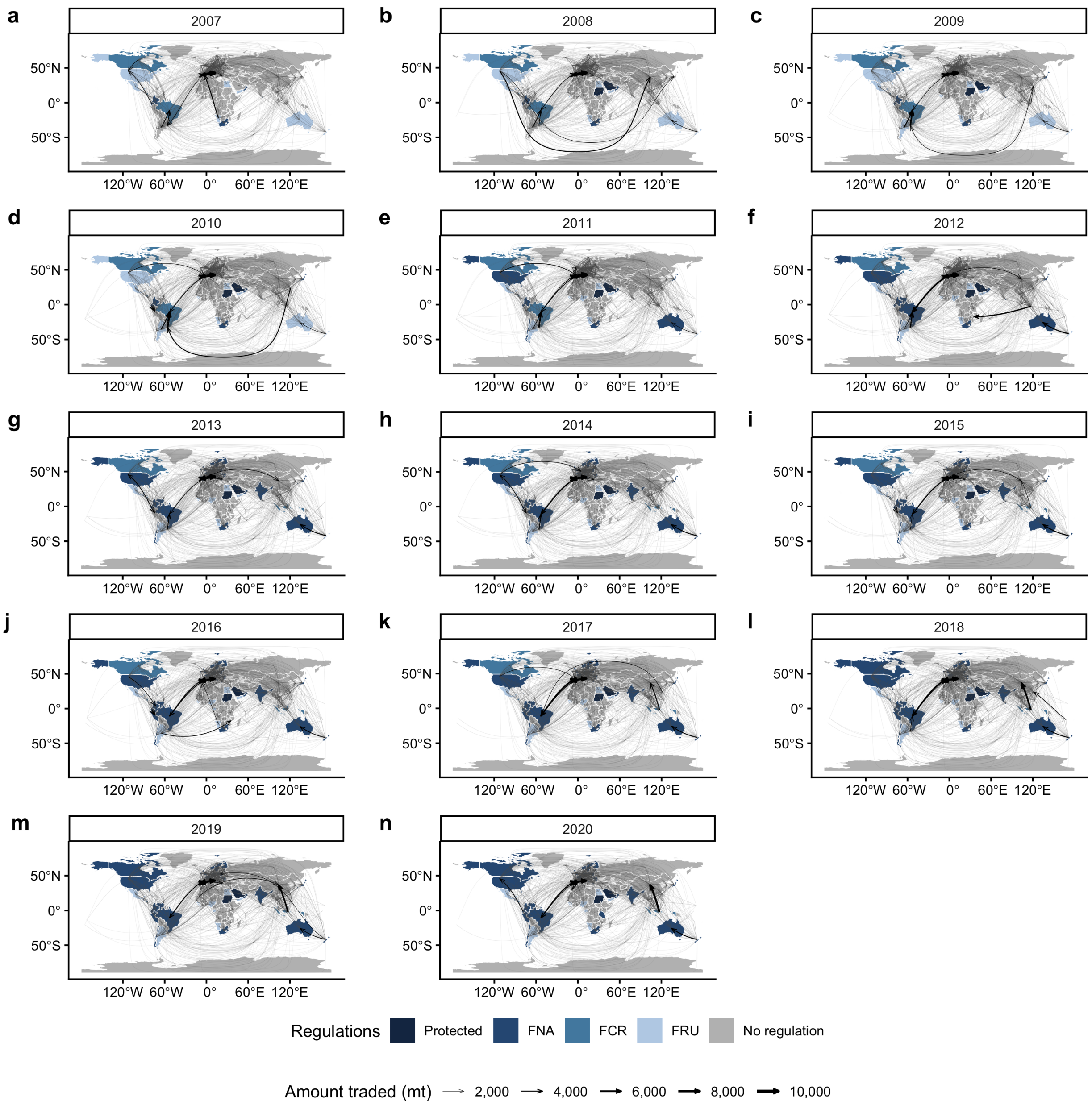

Supplementary Fig. 11: Annual shark product trade flows. Trade flows of shark products each year from 2007 to 2020. Light, thin gray lines represent all trade flows and the darker, thicker lines represent the top 10 trade flows each year. Arrows designate the direction of flow, starting in the exporting country and pointing towards the importing country and are weighted by total amount traded (mt). Shark fishing and finning regulations that were already adopted in each year are colored according to regulation type, with darker colors representing stronger regulations (Protected - sharks are fully protected from fishing; FNA - fins must be naturally attached; FCR - fins can be removed from the animal but must be accompanied by carcasses in a prescribed ratio; FRU - unspecified finning regulations). The world basemap was pulled from the `rnaturalearth` R package [46].

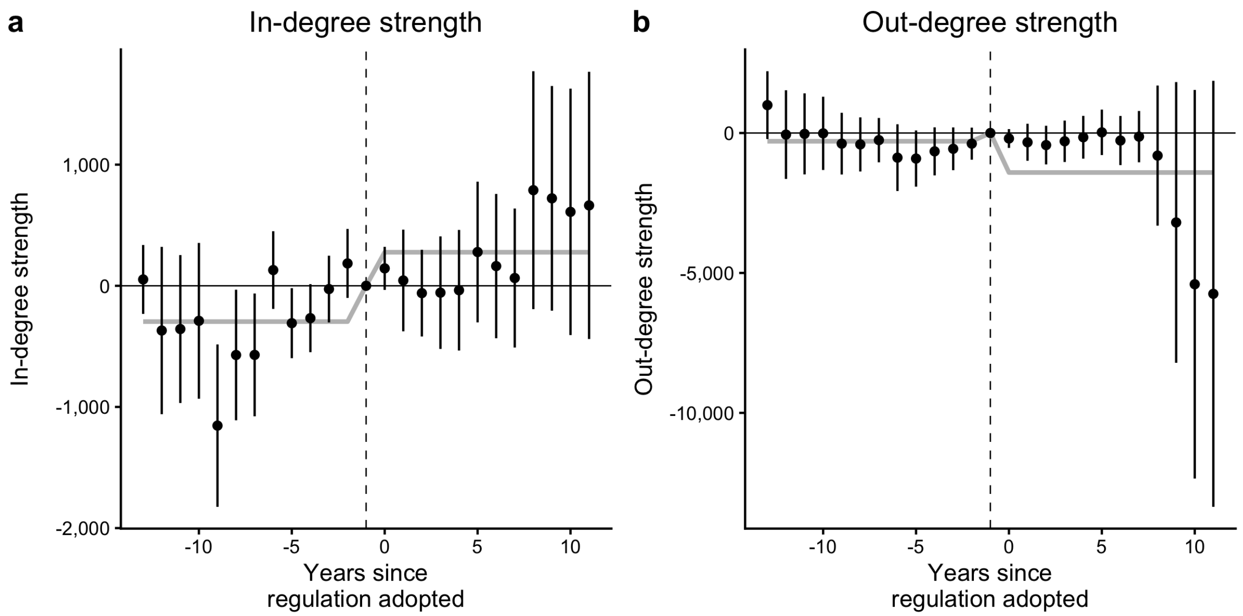

Supplementary Fig. 12: Trends in trade network strength before and after regulation. Difference-in-Difference model results showing the event study plots by year for (a) in-degree strength (n = 785 country-years) and (b) out-degree strength (n = 742 country-years). The event study plot shows the estimated trends before any shark finning regulation is adopted (left of the dashed line at the reference year, year = -1) and dynamic treatment effects in each year following regulation adoption relative to the reference period (right of the dashed line). The study unit is a country-year where the treatment group includes country-years where a shark finning regulation is in place and the control group (n=279) are country-years that have no shark finning regulations. Data are presented as the event-time coefficient (black dots) with 95% confidence intervals. The gray line is the aggregated treatment effect line, which is the mean of all difference estimates before (left of the dashed line) and after (right of the dashed line) regulation adoption. p-values for in-degree and out-degree strength are 0.623 and 0.350, respectively.

Supplementary Table 1: Definitions. Complete definitions for terms used in the manuscript.

Manuscript term	Definition
Exports	All exports (in tons, live weight equivalent) where products were sourced from the same country they were exported from, refered to as domestically-sourced exports in the ARTIS database.
Imports	All products (in tons, live weight equivalent) that were sourced in a different country than the importing country, refered to as foreign-sourced imports in the ARTIS database. Products that are exported from a source country for intermediary processing and then re-imported back to the source country are excluded.
Domestic consumption	Domestic consumption of shark products (in tons, live weight equivalent) are products sourced in the same country they are landed, refered to as domestically-sourced consumption in the ARTIS database. Domestic consumption, also called apparent domestic consumption, is an estimated value in the ARTIS database it is not directly reported.
Shark products	Used to describe all shark products collectively tradedand expressed as a live weight equivalent or the total weight of individuals that would have been caught to achieve reported product weights. To avoid double counting ARTIS only tracks one primary product, for sharks this is shark meat. The live weight equivalent would also capture other parts of the shark that might enter trade (e.g., fins, liver, oil, etc.) though these are not tracked indvidually or explicitly.
In-degree strength	In network analysis, the sum of the weights of all incoming edges to a node. In the shark trade network, this is the the total amount of shark products imported to a given county (in tons, live weight equivalent).
Out-degree strength	In network analysis, the sum of the weights of all outgoing edges from a node. In the shark trade network, this is the the total amount of shark products exported from a given county (in tons, live weight equivalent).
In-degree centrality	In network analysis, the number of incoming edges to a node. In the shark trade network, this is the number of a countries a given country imports from. The normalized in-degree centrality is the number of incoming nodes relative to all possible incoming nodes.
Out-degree centrality	In network analysis, the number of outgoing edges from a node. In the shark trade network, this is the number of countries a given country exports to. The normalized out-degree centrality is the number of outgoing nodes relative to all possible outgoing nodes.

Density	A measure of network connectivity representing the proportion of actual connections in a network relative to the total possible connections. In the shark trade network, this is the number of active country-to-country trade partnerships relative to all possible trade partnerships. Low density means the network is sparse with few active country-to-country links and high density is a very connected network where most countries trade with each.
Modularity	A measure of how well a network can be clustered into distinct clusters. In the shark trade network, high modularity represents regionally clustered trade partnerships and low modularity represents a diverse global network with few distinct groups. Modularity is calculated for an undirected version of the network that considers the total volume of shark products traded but does not distinguish between imports and exports.
Treatment group	A group that receives the intervention being studied (also called a treated group). In this analysis, these are countries that implement shark finning regulations during the study period.
Control group	A group that does not receive treatment and acts as a baseline for comparison. In this analysis, these are countries without shark finning regulations during the study period.
Not-yet-treated	Units that will receive treatment later but have not yet at a given point in time. In this analysis, for a given year, these are countries that will adopt shark finning regulations during the study period but have not yet.
Average treatment effect on the treated (ATT)	The estimated average impact of the intervention among those who were treated, calculated as the average of the difference in outcomes between the control and treatment groups before and after the policy. In this analysis, it represents the average change in shark trade or network outcomes attributable to finning regulations.
Parallel trend assumption	The assumption that without treatment, treated and control groups would have followed the similar trends over time. In this analysis, it means shark trade patterns would have evolved similarly in both groups if no regulations were introduced.
DFBETAS	A diagnostic statistic that measures how much an individual observation influences regression coefficients. In this analysis, it identifies whether a single country-year observation disporportionately affects the estimated treatment effect.

Supplementary Table 2: Product codes. Product codes in the ARTIS database that were included in Difference-in-Difference analyses from 2007-2020.

Product code	Description
030110	Live ornamental fish
030199	Live fish (excl. ornamental fish, trout [Salmo trutta, Oncorhynchus mykiss, Oncorhynchus clarki, Oncorhynchus aguabonita, Oncorhynchus gilae, Oncorhynchus apache and Oncorhynchus chrysogaster], eels [Anguilla spp.], carp, bluefin tunas [Thunnus thynnus] and southern bluefin tunas [Thunnus maccoyii])
030265	Fresh or chilled dogfish and other sharks
030269	Fresh or chilled freshwater and saltwater fish (excl. salmonidae, flat fish, tunas, skipjack or stripe-bellied bonito, herrings, cod, sardines, sardinella, brisling or sprats, haddock, coalfish, mackerel, sharks, eels [Anguilla spp.], swordfish and toothfish)
030375	Frozen dogfish and other sharks
030379	Frozen freshwater and saltwater fish (excl. salmonidae, flat fish, tunas, skipjack or stripe-bellied bonito, herrings, cod, swordfish, toothfish, sardines, sardinella, brisling or sprats, haddock, coalfish, mackerel, dogfish and other sharks, eels [Anguilla spp.], sea bass and hake)
030419	Fresh or chilled fillets and other fish meat ”whether or not minced” (excl. swordfish and toothfish)
030429	Frozen fish fillets (excl. swordfish and toothfish)
030499	Frozen fish meat ”whether or not minced” (excl. swordfish, toothfish and fillets)
030510	Flours, meals and pellets of fish, fit for human consumption
030530	Fish fillets, dried, salted or in brine, not smoked
030549	Smoked fish, incl. fillets (excl. Pacific salmon, Atlantic salmon, Danube salmon and herrings)
030559	Dried fish, salted, not smoked (excl. cod and other fillets)
030569	Fish, salted or in brine only (excl. herrings, cod, anchovies and fillets in general)
160419	Prepared or preserved fish, whole or in pieces (excl. minced and salmon, herrings, sardines, sardinella, brisling or sprats, tunas, skipjack and Atlantic bonito, bonito ”sarda spp.”, mackerel and anchovies)
160420	Prepared or preserved fish (excl. whole or in pieces)
230120	Flours, meals and pellets of fish or crustaceans, molluscs or other aquatic invertebrates, unfit for human consumption

Supplementary Table 3: Difference-in-difference trade results. Aggregated ATT estimates across trade outcomes. All models include country (iso3c) and year fixed effects and control for the World Bank Governance Index and region-specific linear trends. Reported coefficients are the average treatment effect on the treated (ATT) estimates, with the 95% confidence interval shown in brackets below. *p*-values of two-sided t tests for the exports, consumption, and imports models are 0.350, 0.092, and 0.538, respectively.

	Exports	Consumption	Imports
Model:	(1)	(2)	(3)
Variables			
ATT	-350.5	-1,671.6*	141.7
	[-1,092.9; 391.8]	[-3,622.8; 279.6]	[-314.5; 597.9]
Fixed-effects			
iso3c	Yes	Yes	Yes
year	Yes	Yes	Yes
Fit statistics			
Observations	1,022	1,106	1,064
R^2	0.95867	0.87721	0.93483
Within R^2	0.26957	0.09062	0.12423

Clustered (iso3c) co-variance matrix, 95% confidence intervals in brackets
*Signif. Codes: ***: 0.01, **: 0.05, *: 0.1*

Supplementary Table 4: Summary statistics by treatment status. The number of country-years evaluated (N), and the mean and standard deviation (SD) of the outcome (i.e., volume of exports, consumption, imports) and the Worldbank Governance Index (WB Index) for the control group and treatment group for each of the difference-in-difference (DiD) trade models.

		Control			Treated		
DiD Model	Variable	N	Mean	SD	N	Mean	SD
Exports	Outcome	743	618.96	2705.87	279	2010.78	5275.91
Exports	WB Index	743	-0.07	0.92	279	0.71	0.72
Consumption	Outcome	827	1319.14	5578.76	279	2737.83	8979.08
Consumption	WB Index	827	-0.07	0.92	279	0.71	0.72
Imports	Outcome	785	876.79	2310.83	279	1865.69	3584.24
Imports	WB Index	785	-0.05	0.89	279	0.71	0.72

Supplementary Table 5: Difference-in-difference trade results for FNA. Aggregated ATT estimates across trade outcomes for fins naturally attached (FNA) regulations only. All models include country (iso3c) and year fixed effects and control for the World Bank Governance Index and region-specific linear trends. Reported coefficients are the average treatment effect on the treated (ATT) estimates, with the 95% confidence interval shown in brackets below. *p*-values of two-sided t tests for the exports, consumption, and imports models are 0.649, 0.201, and 0.256, respectively.

	Exports	Consumption	Imports
Model:	(1)	(2)	(3)
Variables			
ATT	-123.4	-1,012.4	300.5
	[-662.1; 415.4]	[-2,574.7; 549.9]	[-223.2; 824.2]
Fixed-effects			
iso3c	Yes	Yes	Yes
year	Yes	Yes	Yes
Fit statistics			
Observations	961	1,045	1,003
R^2	0.96789	0.84922	0.94116
Within R^2	0.10021	0.04225	0.14522

Clustered (iso3c) co-variance matrix, 95% confidence intervals in brackets
*Signif. Codes: ***: 0.01, **: 0.05, *: 0.1*

Supplementary Table 6: Difference-in-difference trade results for not yet treated countries. Aggregated ATT estimates across trade outcomes after limiting the control group to include not yet treated countries only (countries that never adopt any finning regulation are excluded from the analysis). All models include country (iso3c) and year fixed effects and control for the World Bank Governance Index and region-specific linear trends. Reported coefficients are the average treatment effect on the treated (ATT) estimates, with the 95% confidence interval shown in brackets below. *p*-values of two-sided t tests for the exports, consumption, and imports models are 1.73e-6, 4.27e-13, and 0.112, respectively.

Model:	Exports (1)	Consumption (2)	Imports (3)
Variables			
ATT	-2,071.0***	6,793.1***	328.5
	[-2,823.9; -1,318.1]	[5,559.2; 8,027.1]	[-80.0; 737.1]
Fixed-effects			
iso3c	Yes	Yes	Yes
year	Yes	Yes	Yes
Fit statistics			
Observations	504	504	504
R^2	0.97022	0.90067	0.95201
Within R^2	0.39233	0.25449	0.14680

Clustered (iso3c) co-variance matrix, 95% confidence intervals in brackets
*Signif. Codes: ***: 0.01, **: 0.05, *: 0.1*

Supplementary Table 7: Difference-in-difference trade results for high catch countries. Aggregated ATT estimates across trade outcomes after limiting the control group to countries with shark catches in at least 10 years of the 14 year time series. All models include country (iso3c) and year fixed effects and control for the World Bank Governance Index and region-specific linear trends. Reported coefficients are the average treatment effect on the treated (ATT) estimates, with the 95% confidence interval shown in brackets below. p-values of two-sided t tests for the exports, consumption, and imports models are 0.649, 0.201, 0.256, respectively.

Model:	Exports<br>(1)	Consumption<br>(2)	Imports<br>(3)
Variables			
ATT	-123.4	-1,012.4	300.5
	[-662.1; 415.4]	[-2,574.7; 549.9]	[-223.2; 824.2]
Fixed-effects			
iso3c	Yes	Yes	Yes
year	Yes	Yes	Yes
Fit statistics			
Observations	961	1,045	1,003
R^2	0.96789	0.84922	0.94116
Within R^2	0.10021	0.04225	0.14522

Clustered (iso3c) co-variance matrix, 95% confidence intervals in brackets
*Signif. Codes: ***: 0.01, **: 0.05, *: 0.1*

Supplementary Table 8: Difference-in-difference trade results by quantile. Aggregated ATT estimates across outcomes, by baseline quantile.Quantiles are based on 2007 baseline trading volumes and network metrics with the first quantile containing highest baseline trading values. Quantile ranges report the minimum–maximum baseline outcome values within each quantile. The fourth quantile contains the lowest baseline trading volumes (all equal to zero in 2007), though countries in this group are active in shark product trade in other years included in the analysis. All models include country (iso3c) and year fixed effects and control for the World Bank Governance Index and region-specific linear trends. Reported coefficients are the average treatment effect on the treated (ATT) estimates, with the 95% confidence interval shown in brackets below. p-values of two-sided t tests for export models are 0.0002, 0.4959, 0.0775, and 0.5115 for quantiles 1-4, respectively. p-values of two-sided t tests for consumption models are 0.0032, 0.2248, 0.0019, and 0.9461 for quantiles 1-4, respectively. p-values of two-sided t tests for import models are 0.0299, 0.0514, 0.8810, and 0.2746 for quantiles 1-4, respectively.

	Exports	Consumption	Imports
**Quantile 1**			
ATT	-145.7***	-114.5***	-297.3**
	[-213.0; -78.4]	[-186.0; -43.1]	[-562.2; -32.5]
Quantile ranges	311-21954	578-42327	169-15084
Observations	280	294	266
**Quantile 2**			
ATT	-768.1	254.9	-128.8*
	[-3,136.2; 1,600.1]	[-169.6; 679.3]	[-258.5; 0.876]
Quantile ranges	2-286	11-547	10-167
Observations	196	294	238
**Quantile 3**			
ATT	-393.2*	-7,920.4***	70.0
	[-834.0; 47.5]	[-12,459.5; -3,381.3]	[-880.0; 1,020.0]
Quantile ranges	0-2	0-11	0-9
Observations	294	252	406
**Quantile 4**			
ATT	-73.7	10.5	7.26
	[-305.4; 158.1]	[-311.5; 332.5]	[-6.74; 21.3]
Quantile ranges	0-0	0-0	0-0
Observations	252	266	154

Clustered (iso3c) co-variance matrix, 95% confidence intervals in brackets.
*Significance codes: *** $p < 0.01$, ** $p < 0.05$, * $p < 0.1$.*

Supplementary Table 9: Difference-in-difference trade results by habitat. Aggregated ATT estimates across trade outcomes after stratifying by habitat groups. All models include country (iso3c) and year fixed effects and control for the World Bank Governance Index and region-specific linear trends. Reported coefficients are the average treatment effect on the treated (ATT) estimates, with the 95% confidence interval shown in brackets below. p-values for models of small coastal shark species are 0.4369, 0.6641, and 0.6844 for exports, consumption, and imports, respectively. p-values of two-sided t tests for models of large coastal shark species are 0.7070, 0.1692, 0.9983, for exports, consumption, and imports, respectively. p-values of two-sided t tests for models of pelagic shark species are 4.28e-15, 0.1341, and 0.3666, for exports, consumption, and imports, respectively. p-values of two-sided t tests of two-sided t tests for models of deep-water shark species are 0.2267, 0.3144, 0.9338, for exports, consumption, and imports, respectively.

	Exports	Consumption	Imports
**Small coastal shark species**			
ATT	-57.1	-43.0	-21.5
	[-180.1; 65.9]	[-243.4; 157.4]	[-126.5; 83.5]
Observations	336	434	1,008
**Large coastal shark species**			
ATT	-142.4	-1,417.6	-0.018
	[-898.0; 613.1]	[-3,455.1; 619.9]	[-17.4; 17.3]
Observations	770	868	910
**Pelagic shark species**			
ATT	-823.7***	-2,005.3	175.8
	[-1,176.8; -470.7]	[-4,660.5; 649.8]	[-209.9; 561.6]
Observations	448	490	1,036
**Deep-water shark species**			
ATT	-66.8	-127.3	-4.04
	[-176.6; 43.0]	[-379.2; 124.6]	[-100.6; 92.6]
Observations	644	658	1,064

Clustered (iso3c) co-variance matrix, 95% confidence intervals in brackets.
*Significance codes: *** $p < 0.01$, ** $p < 0.05$, * $p < 0.1$.*

Supplementary Table 10: Difference-in-difference trade results with additional controls. Aggregated ATT estimates across trade outcomes after additional controls for population and per capita gross domestic product. All models include country (iso3c) and year fixed effects and control for the World Bank Governance Index and region-specific linear trends. Reported coefficients are the average treatment effect on the treated (ATT) estimates, with the 95% confidence interval shown in brackets below. p-values of two-sided t tests for the exports, consumption, and imports models are 0.409, 0.073, and 0.491, respectively.

Model:	Exports (1)	Consumption (2)	Imports (3)
Variables			
ATT	-300.4	-1,851.4*	174.8
	[-1,022.0; 421.3]	[-3,878.3; 175.5]	[-328.1; 677.8]
Fixed-effects			
iso3c	Yes	Yes	Yes
year	Yes	Yes	Yes
Fit statistics			
Observations	1,008	1,092	1,064
R^2	0.95900	0.87945	0.94607
Within R^2	0.27599	0.10747	0.27534

Clustered (iso3c) co-variance matrix, 95% confidence intervals in brackets
*Signif. Codes: ***: 0.01, **: 0.05, *: 0.1*

Supplementary Table 11: Standardized DFBETA by model type. The top 5 countries within each model ranked by the standardized DFBETA (DFBETAS) value. The average treatment effect on the treated (ATT) estimate, ATT standard error, and p-value of the two-sided t test against the null hypothesis for the ATT estimates are provided when each model is run without the listed country.

Model	Country	ATT	ATT standard error	p-value	DFBETAS
Exports	Guinea	-554.382	275.486	0.05	0.74
Exports	Spain	-139.961	299.504	0.64	0.70
Exports	Argentina	-199.545	272.532	0.47	0.55
Exports	Portugal	-426.634	376.944	0.26	0.20
Exports	Taiwan	-279.287	387.027	0.47	0.18
Consumption	Indonesia	-937.902	749.598	0.21	0.98
Consumption	Spain	-994.703	697.514	0.16	0.97
Consumption	Malta	-1852.245	1049.304	0.08	0.17
Consumption	Malaysia	-1757.569	1025.983	0.09	0.08
Consumption	Turkey	-1753.131	1029.600	0.09	0.08
Imports	Uruguay	38.310	215.295	0.86	0.48
Imports	Portugal	43.101	205.429	0.83	0.48
Imports	China	228.524	212.890	0.29	0.41
Imports	Spain	56.561	213.044	0.79	0.40
Imports	Italy	213.891	221.743	0.34	0.33

Supplementary Table 12: Difference-in-difference network centrality results. Aggregated ATT estimates across degree centrality outcomes. All models include country (iso3c) and year fixed effects and control for the World Bank Governance Index and region-specific linear trends. Reported coefficients are the average treatment effect on the treated (ATT) estimates, with the 95% confidence interval shown in brackets below. *p*-values of two-sided t tests for the in-degree and out-degree centrality models are 0.663 and 0.837, respectively.

	In-degree centrality	Out-degree centrality
Model:	(1)	(2)
Variables		
ATT	0.001	0.001
	[-0.005; 0.007]	[-0.013; 0.015]
Fixed-effects		
iso3c	Yes	Yes
year	Yes	Yes
Fit statistics		
Observations	1,064	1,022
R^2	0.96636	0.95417
Within R^2	0.20308	0.29471

Clustered (iso3c) co-variance matrix, 95% confidence intervals in brackets
*Signif. Codes: ***: 0.01, **: 0.05, *: 0.1*

Supplementary Table 13: Network summary statistics by treatment status. The number of country-years evaluated (N), and the mean and standard deviation (SD) of the outcome (i.e., in- and out-degree centrality) and the Worldbank Governance Index (WB Index) for the control group and treatment group for each of the difference-in-difference (DiD) models.

		Control			Treated		
DiD Model	Variable	N	Mean	SD	N	Mean	SD
In-degree centrality	Outcome	785	0.05	0.06	279	0.08	0.06
In-degree centrality	WB Index	785	-0.05	0.89	279	0.71	0.72
Out-degree centrality	Outcome	743	0.06	0.09	279	0.12	0.14
Out-degree centrality	WB Index	743	-0.07	0.92	279	0.71	0.72

Supplementary Table 14: Difference-in-difference network strength results. Aggregated ATT estimates across degree strength outcomes. All models include country (iso3c) and year fixed effects and control for the World Bank Governance Index and region-specific linear trends. Reported coefficients are the average treatment effect on the treated (ATT) estimates, with the 95% confidence interval shown in brackets below. p-values of two-sided t tests for the in-degree and out-degree strength models are 0.623 and 0.350, respectively.

Model:	In-degree strength (1)	Out-degree strength (2)
Variables		
ATT	102.9	-350.3
	[-312.4; 518.2]	[-1,092.4; 391.8]
Fixed-effects		
iso3c	Yes	Yes
year	Yes	Yes
Fit statistics		
Observations	1,064	1,022
R^2	0.92583	0.95867
Within R^2	0.12221	0.26967

Clustered (iso3c) co-variance matrix, 95% confidence intervals in brackets
*Signif. Codes: ***: 0.01, **: 0.05, *: 0.1*

Supplementary Table 15: Difference-in-difference network centrality results for FNA. Aggregated ATT estimates across degree centrality outcomes for fins naturally attached (FNA) regulations only. All models include country (iso3c) and year fixed effects and control for the World Bank Governance Index and region-specific linear trends. Reported coefficients are the average treatment effect on the treated (ATT) estimates, with the 95% confidence interval shown in brackets below. *p*-values of two-sided t tests for the in-degree and out-degree centrality models are 0.198 and 0.716, respectively.

Model:	In-degree centrality<br>(1)	Out-degree centrality<br>(2)
Variables		
ATT	0.004	0.003
	[-0.002; 0.011]	[-0.014; 0.020]
Fixed-effects		
iso3c	Yes	Yes
year	Yes	Yes
Fit statistics		
Observations	1,003	961
R^2	0.96866	0.94353
Within R^2	0.20960	0.25137

Clustered (iso3c) co-variance matrix, 95% confidence intervals in brackets
*Signif. Codes: ***: 0.01, **: 0.05, *: 0.1*

Supplementary Table 16: Difference-in-difference network centrality results for not yet treated countries. Aggregated ATT estimates across degree centrality outcomes after limiting the control group to include not yet treated countries only (countries that never adopt any finning regulation are excluded from the analysis). All models include country (iso3c) and year fixed effects and control for the World Bank Governance Index and region-specific linear trends. Reported coefficients are the average treatment effect on the treated (ATT) estimates, with the 95% confidence interval shown in brackets below. *p*-values of two-sided t tests for the in-degree and out-degree centrality models are 0.058 and 0.932, respectively.

	In-degree centrality	Out-degree centrality
Model:	(1)	(2)
Variables		
ATT	0.009*	0.0006
	[-0.0003; 0.018]	[-0.014; 0.016]
Fixed-effects		
iso3c	Yes	Yes
year	Yes	Yes
Fit statistics		
Observations	504	504
R^2	0.96818	0.97156
Within R^2	0.27825	0.44892

Clustered (iso3c) co-variance matrix, 95% confidence intervals in brackets
*Signif. Codes: ***: 0.01, **: 0.05, *: 0.1*

Supplementary Table 17: Difference-in-difference network centrality results for high catch countries. Aggregated ATT estimates across degree centrality outcomes after limiting the control group to countries with shark catches in at least 10 years of the 14 year time series. All models include country (iso3c) and year fixed effects and control for the World Bank Governance Index and region-specific linear trends. Reported coefficients are the average treatment effect on the treated (ATT) estimates, with the 95% confidence interval shown in brackets below. *p*-values of two-sided t tests for the in-degree and out-degree centrality models are 0.198 and 0.716, respectively.

	In-degree centrality	Out-degree centrality
Model:	(1)	(2)
Variables		
ATT	0.004	0.003
	[-0.002; 0.011]	[-0.014; 0.020]
Fixed-effects		
iso3c	Yes	Yes
year	Yes	Yes
Fit statistics		
Observations	1,003	961
R^2	0.96866	0.94353
Within R^2	0.20960	0.25137

Clustered (iso3c) co-variance matrix, 95% confidence intervals in brackets
*Signif. Codes: ***: 0.01, **: 0.05, *: 0.1*

Supplementary Table 18: Difference-in-difference network centrality results by quantile. Aggregated ATT estimates across degree centrality outcomes, by baseline quantile. Quantiles are based on 2007 baseline trading volumes and network metrics with the first quantile containing highest baseline trading values. Quantile ranges report the minimum–maximum baseline outcome values within each quantile. All models include country (iso3c) and year fixed effects and control for the World Bank Governance Index and region-specific linear trends. Reported coefficients are the average treatment effect on the treated (ATT) estimates, with the 95% confidence interval shown in brackets below. p-values of two-sided t tests for in-degree centrality models are 0.123, 0.789, and 0.586 for quantiles 1-3, respectively. p-values of two-sided t tests for out-degree centrality models are 0.066, 0.982, and 0.216 for quantiles 1-3, respectively.

	In-degree centrality	Out-degree centrality
**Quantile 1**		
ATT	-0.010	0.014*
	[-0.023; 0.003]	[-0.0010; 0.028]
Quantile ranges	0.05-0.27	0.09-0.69
Observations	252	266
**Quantile 2**		
ATT	-0.002	-0.001
	[-0.013; 0.010]	[-0.097; 0.095]
Quantile ranges	0.02-0.05	0.02-0.09
Observations	322	210
**Quantile 3**		
ATT	0.005	-0.004
	[-0.012; 0.021]	[-0.011; 0.003]
Quantile ranges	0-0.02	0-0.02
Observations	350	308

Clustered (iso3c) co-variance matrix, 95% confidence intervals in brackets.
*Significance codes: *** $p < 0.01$, ** $p < 0.05$, * $p < 0.1$.*

Supplementary Table 19: Difference-in-difference network centrality results by habitat. Aggregated ATT estimates across degree centrality outcomes after stratifying by habitat groups. All models include country (iso3c) and year fixed effects and control for the World Bank Governance Index and region-specific linear trends.Reported coefficients are the average treatment effect on the treated (ATT) estimates, with the 95% confidence interval shown in brackets below. p-values for models of small coastal shark species are 0.676 and 0.034 for in-degree and out-degree centrality, respectively. p-values of two-sided t tests for models of large coastal shark species are 0.775 and 0.352 for in-degree and out-degree centrality, respectively. p-values of two-sided t tests for models of pelagic shark species are 0.716 and 0.915 for in-degree and out-degree centrality, respectively. p-values of two-sided t tests for models of deep-water shark species are 0.219 and 0.745 for in-degree and out-degree centrality, respectively.

	In-degree centrality	Out-degree centrality
**Small coastal shark species**		
ATT	0.001	-0.041**
	[-0.004; 0.006]	[-0.079; -0.003]
Observations	1,008	448
**Large coastal shark species**		
ATT	0.001	-0.010
	[-0.006; 0.008]	[-0.032; 0.011]
Observations	854	770
**Pelagic shark species**		
ATT	-0.001	0.003
	[-0.007; 0.005]	[-0.052; 0.058]
Observations	994	336
**Deep-water shark species**		
ATT	0.003	-0.004
	[-0.002; 0.009]	[-0.025; 0.018]
Observations	1,064	644

Clustered (iso3c) co-variance matrix, 95% confidence intervals in brackets.
*Significance codes: *** $p < 0.01$, ** $p < 0.05$, * $p < 0.1$.*

Supplementary Table 20: Difference-in-difference network centrality results with additional controls. Aggregated ATT estimates across degree centrality outcomes after additional controls for population and per capita gross domestic product. All models include country (iso3c) and year fixed effects and control for the World Bank Governance Index and region-specific linear trends. Reported coefficients are the average treatment effect on the treated (ATT) estimates, with the 95% confidence interval shown in brackets below. *p*-values of two-sided t tests for in-degree and out-degree centrality models are 0.811 and 0.827, respectively.

	In-degree centrality	Out-degree centrality
Model:	(1)	(2)
Variables		
ATT	0.0007	0.002
	[-0.005; 0.007]	[-0.013; 0.016]
Fixed-effects		
iso3c	Yes	Yes
year	Yes	Yes
Fit statistics		
Observations	1,064	1,008
R^2	0.96667	0.95436
Within R^2	0.21050	0.30123

Clustered (iso3c) co-variance matrix, 95% confidence intervals in brackets
*Signif. Codes: ***: 0.01, **: 0.05, *: 0.1*

Supplementary Table 21: Standardized DFBETA by model type. The top 5 countries within each model ranked by the standardized DFBETA (DFBETAS) value. The average treatment effect on the treated (ATT) estimate, ATT standard error, and p-value of the two-sided t test against the null hypothesis for the ATT estimates are provided when each model is run without the listed country.

Model	Country	ATT	ATT standard error	p-value	DFBETAS
In-degree centrality	Guinea	0.003	0.003	0.32	0.56
In-degree centrality	Taiwan	0.000	0.003	0.95	0.39
In-degree centrality	Russia	0.000	0.003	0.93	0.38
In-degree centrality	Indonesia	0.002	0.003	0.47	0.28
In-degree centrality	Dominican Republic	0.001	0.003	0.84	0.24
Out-degree centrality	Taiwan	0.005	0.007	0.46	0.53
Out-degree centrality	Indonesia	0.004	0.007	0.51	0.45
Out-degree centrality	United Kingdom	-0.001	0.007	0.87	0.38
Out-degree centrality	Turkey	-0.001	0.007	0.93	0.30
Out-degree centrality	Uruguay	-0.001	0.007	0.94	0.27
