# Supplementary material for: Global trade responses to shark finning regulations
Source: Nat Commun. 2026 Jul 31;17:6821. doi: 10.1038/s41467-026-75625-1 (PMC13427823; doi:10.1038/s41467-026-75625-1)
Supplement: Supplementary file 2 — Description of Additional Supplementary Files [file 41467_2026_75625_MOESM2_ESM.pdf]

Description of Additional Supplementary Files

File Name: Supplementary Data 1
Description: Shark species in the ARTIS database that were included in differencein-difference analyses from 2007-2020. For each reported species or species group (*scientific_name*), lists the habitat group classifications (*habitat_group*) based on depth ranges and habitat preferences reported in FishBase and the CITES status (*cites_listing*). CITES listing is I if the species is listed in Appendix I, II if the species is listed in Appendix II, and NA otherwise. Percentages of total exports (*percent_total_exports*), consumption (*percent_total_consumption*), and imports (*percent_total_imports*) were calculated as the percent of the total live weight equivalent for all years evaluated, “Not present” indicates the species was not reported.

File Name: Supplementary Data 2
Description: Annual country-level statistics. In each country-year, lists the shark finning regulation in place (*regulation_type*), total volume of domestic exports (metric tons, live-weight equivalent; *total_domestic_exports_mt*), total volume of domestic consumption (metric tons, live-weight equivalent; *total_domestic_consumption_mt*) , total volume of foreign imports (metric tons, live-weight equivalent; *total_imports_mt*), in- and out-degree centrality (*in_degree_centrality*, *out_degree_centrality*), and inand out-degree strength (*in*_degree_strength, *out_degree_strength*). Centrality and strength metrics are further defined in Supplementary Table 1. “Not reported” indicates country-years where trade or consumption data were missing, “Not calculated” represents country-years where network metrics could not be calculated.
