# Supplementary material for: Global trade responses to shark finning regulations
Source: Nat Commun. 2026 Jul 31;17:6821. doi: 10.1038/s41467-026-75625-1 (PMC13427823; doi:10.1038/s41467-026-75625-1)
Supplement: Supplementary file 5 — Reporting Summary [file 41467_2026_75625_MOESM5_ESM.pdf]

nature portfolio

Corresponding author(s): Sara Orofino

Last updated by author(s): Jun 10, 2026

Reporting Summary

Nature Portfolio wishes to improve the reproducibility of the work that we publish. This form provides structure for consistency and transparency in reporting. For further information on Nature Portfolio policies, see our Editorial Policies and the Editorial Policy Checklist.

Please do not complete any field with "not applicable" or n/a. Refer to the help text for what text to use if an item is not relevant to your study.
For final submission: please carefully check your responses for accuracy; you will not be able to make changes later.

Statistics

For all statistical analyses, confirm that the following items are present in the figure legend, table legend, main text, or Methods section.

n/a	Confirmed	
☐	☒	The exact sample size (*n*) for each experimental group/condition, given as a discrete number and unit of measurement
☐	☒	A statement on whether measurements were taken from distinct samples or whether the same sample was measured repeatedly
☐	☒	The statistical test(s) used AND whether they are one- or two-sided<br>*Only common tests should be described solely by name; describe more complex techniques in the Methods section.*
☐	☒	A description of all covariates tested
☐	☒	A description of any assumptions or corrections, such as tests of normality and adjustment for multiple comparisons
☐	☒	A full description of the statistical parameters including central tendency (e.g. means) or other basic estimates (e.g. regression coefficient) AND variation (e.g. standard deviation) or associated estimates of uncertainty (e.g. confidence intervals)
☐	☒	For null hypothesis testing, the test statistic (e.g. *F*, *t*, *r*) with confidence intervals, effect sizes, degrees of freedom and *P* value noted<br>*Give P values as exact values whenever suitable.*
☒	☐	For Bayesian analysis, information on the choice of priors and Markov chain Monte Carlo settings
☒	☐	For hierarchical and complex designs, identification of the appropriate level for tests and full reporting of outcomes
☒	☐	Estimates of effect sizes (e.g. Cohen's *d*, Pearson's *r*), indicating how they were calculated

Our web collection on statistics for biologists contains articles on many of the points above.

Software and code

Policy information about availability of computer code

Data collection

No software was used or created for data collection.

Data analysis

All data analysis was done using R version 4.6.0 and all code developed and used in the study is available for download and archived on Zenodo under https://doi.org/10.5281/zenodo.15801243.

We used the following R packages in our research:
arrow v24.0.0
countrycode v1.8.0
cowplot v1.2.0
eurostat v4.0
fixest v0.14.1
geosphere v1.6-8
ggfixest v0.4.0
ggpattern v1.3.1
ggpubr v0.6.3
here v1.0.2
igraph v2.3.2
janitor v2.2.1
purrr v1.2.2
quarto v1.5.1
readxl v1.4.5

rnaturalearth v1.2.0
rnaturalearthdata v1.0.0
sf v1.1-0
targets v1.12.0
tarchetypes v0.14.1
tibble v3.3.1
tidyverse v2.0.0

For manuscripts utilizing custom algorithms or software that are central to the research but not yet described in published literature, software must be made available to editors and reviewers. We strongly encourage code deposition in a community repository (e.g. GitHub). See the Nature Portfolio guidelines for submitting code & software for further information.

Data

Policy information about availability of data

All manuscripts must include a data availability statement. This statement should provide the following information, where applicable:

- Accession codes, unique identifiers, or web links for publicly available datasets
- A description of any restrictions on data availability
- For clinical datasets or third party data, please ensure that the statement adheres to our policy

The trade data used in this study are from the Aquatic Resource Trade in Species database, available at https://doi.org/10.5063/F1CZ35N7. The World Bank Governance Indicators used in this study are the 2024 update, available from www.govindicators.org. The population and income data used in the study are from the 2024 update of the World Development Indicators, available from https://data.worldbank.org/indicator/SP.POP.TOTL and https://data.worldbank.org/indicator/NY.GDP.PCAP.PP.KD, respectively. Secondary income data used in this study are from the Penn World Table Version 11, available from http://www.ggdc.net/pwt. Additional source datasets developed for this analysis are included in the Zenodo code repository available at https://doi.org/10.5281/zenodo.15801243. Additional data that were generated in this study and used to create the figures are provided in the Supplementary Data files.

Research involving human participants, their data, or biological material

Policy information about studies with human participants or human data. See also policy information about sex, gender (identity/presentation), and sexual orientation and race, ethnicity and racism.

Reporting on sex and gender	NA
Reporting on race, ethnicity, or other socially relevant groupings	NA
Population characteristics	NA
Recruitment	NA
Ethics oversight	NA

Note that full information on the approval of the study protocol must also be provided in the manuscript.

Field-specific reporting

Please select the one below that is the best fit for your research. If you are not sure, read the appropriate sections before making your selection.

☐ Life sciences ☐ Behavioural & social sciences ☒ Ecological, evolutionary & environmental sciences

Ecological, evolutionary & environmental sciences study design

All studies must disclose on these points even when the disclosure is negative.

Study description	We used an observational country-year panel design to evaluate whether national shark finning regulations were associated with changes in shark product trade. The treatment was adoption of a shark finning regulation, and outcomes were annual country-level domestically sourced exports, domestically sourced consumption, foreign-sourced imports, and trade network metrics (in-degree and out-degree centrality). We estimated staggered difference-in-differences event-study models with country and year fixed effects, the World Bank Voice and Accountability index, region-specific linear trends, and country-clustered standard errors for treatment effect inference. For the difference-in-difference for exports and out-degree centrality, we evaluate 1,022 observations across 73 unique countries (743 country-years in the treatment group, 279 country-years in the control group); for imports and in-degree centrality, we evaluate 1,064 observations across 76 countries (785 country-years in the treatment group, 279 country-years in the control group); and for consumption, we evaluate 1,106 observations across 79 countries (827 country-years in the treatment group, 279 country-years in the control group). Six sets of robustness checks were run for the difference-in-difference analysis to assess the credibility of the findings and a sensitivity analysis was completed using a leave-one-out approach and the diagnostic

statistic DFBETAS.

Research sample

The sample consisted of country-year observations where each observation included the country, year, shark finning regulation in place that year, and the relevant trade or network value (e.g., metric tons of exported shark products). Depending on the trade or network metric, sample sizes ranged from 1,022 observations (exports and out-degree centrality) to 1,106 observations (domestic consumption). Data were obtained from two existing global datasets: (1) the shark finning/fishing regulations dataset (Worm et al. 2024) which includes all countries and territories with shark finning regulations, and (2) the Aquatic Resource Trade in Species (ARTIS) database (Gephart et al. 2024), which represents all shark products that have been reported as landed by the FAO, or traded by the CEPII BACI database, by country and year. The analysis represented shark-producing and shark-trading countries active in global trade from 2007–2020. ARTIS records were restricted to true sharks and excluded rays and records labeled only as "elasmobranchii". For our time period of interest, the ARTIS database includes 123 unique shark taxonomic identifiers, reported at either the species (~83% of all taxonomic identifiers), genus (~9%), family (~6%), or order (~2%) level, traded or consumed under 17 unique product codes by 188 countries. No sex and age range data are available in the ARTIS database.

Sampling strategy

No primary field or lab data were collected for this project. No prospective sample-size calculation was performed because this was an observational study based on existing global data. We used the full set of eligible country-year observations available from the underlying datasets after applying predefined analytical restrictions including requiring at least two pre-treatment years for treated countries for difference-in-difference analysis. This sample covers all 188 countries that trade or consume shark products. For the difference-in-difference for exports and out-degree centrality, we evaluate 1,022 observations across 73 countries (743 country-years in the treatment group, 279 country-years in the control group); for imports and in-degree centrality, we evaluate 1,064 observations across 76 countries (785 country-years in the treatment group, 279 country-years in the control group); and for consumption, we evaluate 1,106 observations across 79 countries (827 country-years in the treatment group, 279 country-years in the control group).

Data collection

We did not collect primary field or laboratory data. Regulation histories were compiled from the published shark regulation dataset by Worm et al. (2024) with data verification by E.B., S.O., K.W., and G.G.M. Trade, consumption and network outcomes were derived from ARTIS records published by Gephart et al. (2024) and downloaded by E.B., S.O., and K.W. World Bank Governance indicators were downloaded by S.O., and additional World Bank income and population data and Penn World Table were downloaded by K.W. Documentation of all data sources used in this study with citations and download links were compiled as part of the project code repository by S.O. and is available at https://doi.org/10.5281/zenodo.15801243.

Timing and spatial scale

The regulation dataset includes annual country-level observations from 1980–2022, and the Aquatic Resource Trade in Species database includes annual country-level observations from 1996-2020. The quasi-experimental analyses used annual data from 2007–2020, the 2007 Harmonized System (HS) codes for shark products were the most highly resolved HS system that still provided adequate pre- and post-treatment observations for a majority of countries with shark finning regulations. We used annual country-level observations because treatment timing and ARTIS trade estimates were harmonized by year. The study was global in spatial scale and conducted at the country level.

Data exclusions

Exclusions were predefined by the research design. We excluded rays because they are not finned and trade records reported only as "elasmobranchii" because the volume of true sharks could not be determined. We excluded countries that implemented shark sanctuaries from the difference-in-differences analyses because this represents a fundamentally different type of policy invention. Finally, we excluded 3 treated countries that first adopted anti-finning regulations before 2009, to ensure each treated unit would have at least two pre-treatment years for pretrend examination. We also excluded re-imported products originally sourced in the importing country from the foreign-sourced import metric to more clearly evaluate the trends of exports from and to producing countries.

Reproducibility

This study did not involve laboratory or field experiments. Reproducibility was supported through code-based analyses, public data and code availability, and multiple robustness checks, including leave-one-out influence analyses, alternative treatment definitions, and alternative control-group specifications.

Randomization

Countries were not randomly assigned to treatments because regulation adoption occurred through real-world policy processes. We addressed this using a staggered difference-in-differences design, with country and year fixed effects, governance covariates, and region-specific linear trends controlled. Parallel trends were assessed using event-study estimates.

Blinding

Because we did not collect the data and this study used existing country-level policy and trade datasets, blinding was not relevant in this study. Treatment timing and outcomes were defined from documented databases and computational processing steps.

Did the study involve field work? ☐ Yes ☒ No

Reporting for specific materials, systems and methods

We require information from authors about some types of materials, experimental systems and methods used in many studies. Here, indicate whether each material, system or method listed is relevant to your study. If you are not sure if a list item applies to your research, read the appropriate section before selecting a response.

Materials & experimental systems

n/a	Involved in the study
☒	☐ Antibodies
☒	☐ Eukaryotic cell lines
☒	☐ Palaeontology and archaeology
☒	☐ Animals and other organisms
☒	☐ Clinical data
☒	☐ Dual use research of concern
☒	☐ Plants

Methods

n/a	Involved in the study
☒	☐ ChIP-seq
☒	☐ Flow cytometry
☒	☐ MRI-based neuroimaging

Plants

Seed stocks	NA
Novel plant genotypes	NA
Authentication	NA

This checklist template is licensed under a Creative Commons Attribution 4.0 International License, which permits use, sharing, adaptation, distribution and reproduction in any medium or format, as long as you give appropriate credit to the original author(s) and the source, provide a link to the Creative Commons license, and indicate if changes were made. The images or other third party material in this article are included in the article's Creative Commons license, unless indicated otherwise in a credit line to the material. If material is not included in the article's Creative Commons license and your intended use is not permitted by statutory regulation or exceeds the permitted use, you will need to obtain permission directly from the copyright holder. To view a copy of this license, visit http://creativecommons.org/licenses/by/4.0/
