# Supplementary material for: Global trade responses to shark finning regulations
Source: Nat Commun. 2026 Jul 31;17:6821. doi: 10.1038/s41467-026-75625-1 (PMC13427823; doi:10.1038/s41467-026-75625-1)
Supplement: Supplementary file 6 — Transparent Peer Review file [file 41467_2026_75625_MOESM6_ESM.pdf]

nature portfolio

Peer Review File

Global trade responses to shark finning regulations

Corresponding Author: Ms Sara Orofino

This file contains all reviewer reports in order by version, followed by all author rebuttals in order by version.

Version 0:

Reviewer comments:

Reviewer #1

(Remarks to the Author)
This was a very well written, well organized, and well executed study that attempted to answer the extremely challenging question of what effect shark finning regulations have had on shark landings, trade (import and export) and consumption. The fundamental challenge to this analysis is that there have not been global efforts to curb shark mortality as stated in L78, there have been global efforts to curb shark finning. Those are not synonymous.

Although shark fins have historically been the most valuable due to high demand from Asian markets, there were robust shark markets in the top shark consuming and trade countries (quartile 1 in this analysis) for shark products before the shark fin ban and those markets have grown. That growth could be related to shark fin regulations but it is just as likely that these changes are due to increased demand for seafood, growth of shark markets, or population growth (similar to patterns found in Brazil, Pincinato et al). While I appreciate the global and comprehensive nature of the network analysis of trade and the DiD , the most compelling element of this analysis is the innovative use of ARTIS as a means of tracking shark production, which ultimately is an indicator of shark landings and catch, which I think is what you mean when you say "providing a direct link between trade flows and ecological research" [L136]

This analytical framework is powerful and has much to offer but in it's current form, the manuscript feels somewhat of a missed opportunity. The findings confirm what Worm et al 2024 found - global shark mortality continues to rise. However, what's exciting is that your global trade flows and trends of production (domestic consumption, domestic export, international imports) may be critical to identifying the drivers of the global shark markets and identify conditions of more sustainable shark consumption.

Specific line comments
Line 81: Although you indicate what artificially attached fins are in the methods, the reader won’t see that until the end of the paper. I suggest just adding a short description of what this means.
Line 139 - 165: This is a rather long paragraph. I suggest splitting it into two or modifying some parts to make it more streamlined. For example I think the description of node strength “156 - 160” can be moved to the results section, where you get into the network analysis and analyze changes to the network. As this format for this journal has the results coming before the methods I think this should be appropriate.

Line 173 - 174 & throughout: Make sure all tables and figures (in the main doc and supplement) are ordered by appearance. I found that they are currently ordered as if this paper was structured in the more conventional format where the methods come after the introduction.

Line 203: Was this analysis conducted on an annual basis? Figure 4 presents results for three different years, and I think it would be helpful if this were stated more explicitly in the text.

Line 290 - 292: I find it difficult to interpret the result that, based on the leave-one-out approach, Argentina was identified as the largest contributor to the DiD model results for node strength. It would be helpful if the authors could provide additional text to contextualize what this finding means, as I did not see such explanation in the results or discussion sections. Without this context, it is unclear whether Argentina represents a meaningful driver of the observed patterns or simply functions as an outlier that disproportionately influences the results.

Line 388: True. I think adding in CITES analysis could help support this more but I understand that could be future work.

Line 411: I suggest adding FAA for consistency

Lines 428 - 433: While these sentences are well written I found myself having to reread it a couple of times to grasp what it conveys. I think a very simple equation may help readers clearly and intuitively understand what is the main metric ARTIS calculates. For example something like:
Exports=Domestic Production+Imports−Domestic Consumption−Processing Losses

Lines 465 - 468: Again not a poorly written sentence but rather long and windy due to the nature of what's being explained. I think breaking it up into two sentences would help make it more interpretable.

Lines 512: Any reasoning for 2 years? Does is have to do with the lag seen with CITES

Table and Figures

Table S8: The supplement has several very large tables that span several pages. This is one that I suggest can be converted into a figure to make it more easily interpreted than spanning 3 pages.

Figure S1: I am finding it hard to interpret what each quantile exactly means. Is quantile 1 the countries with the largest baselines of trade volumes or is it quantile 4? I suggest providing the ranges of these for each quantile bin in the figure caption or in the figure itself.

Figures S3 - 13 & S19 - 24: I understand why the authors constructed the figures this way to show each country's time series. However, it gets overwhelming for the reader and hard for them to visually see the changes. I suggest the authors try and collapse them into just a couple of plots which could be binned by countries that adopted regulation vs ones that don't or by continent, so that readers can see the overall trend across the countries and are better able to compare across countries.

Figure 4: This is a great figure, however, it's rather small and hard to see. I suggest making it vertically oriented so that each map can be larger.

(Remarks on code availability)
I clicked on the link to review the code on Zenodo and it is restricted so I was unable to see the posted code.

Reviewer #2

(Remarks to the Author)
I co-reviewed this manuscript with one of the reviewers who provided the listed reports. This is part of the Nature Communications initiative to facilitate training in peer review and to provide appropriate recognition for Early Career Researchers who co-review manuscripts.

(Remarks on code availability)

Reviewer #3

(Remarks to the Author)
The authors have performed an interesting and timely analysis of emerging shark conservation issues. The ARTIS database is an interesting dataset that seems appropriate for challenges like this. This work is potentially important and impactful, but I have some concerns about framing, analysis, and presentation that I'd like to see addressed.

Sharks are some of the most threatened animals on Earth, there has been intense public interest surrounding their conservation, and some conservation measures may have had previously-unexamined unintended consequences. I agree with all of this as a basis for the analysis. I also agree that the shark meat trade has increased as the shark fin trade and shark finning has declined, and that shark finning has become more regulated.

However, some of the specific framing used to justify this analysis- that the shark meat trade has increased potentially BECAUSE shark finning has become more strictly regulated- is questionable.
Indeed, one of the papers cited in the introduction to justify this claim (reference 12) explicitly tested this hypothesis and found that it wasn't really the case: "This increase may be a consequence of stricter finning regulations that has created incentives for full utilization of sharks and exposed the resource to a new source of demand… Results indicate that shark meat is not a new market in Brazil, but an old one." The other reference (reference 8) acknowledges that this may be part of the issue, but isn't the only issue: "Meanwhile, a combination of demand growth and antifinning regulations intended to encourage the full utilization of carcasses has seen the market for shark meat expand considerably."

I am also confused by some potentially needlessly complex terminology. Are not all exports domestically sourced by definition? Are not all imports foreign sourced by definition? Why not just say imports, exports, and local consumption?

The analyses used in this study also cannot really address causality, they can analyze what's happening when, but not necessarily why or in response to what. That relies on input and analysis from the researchers. The approach of treating countries with anti-finning regulations as the experimental variable and those without anti-finning regulations as control makes a lot of assumptions that I am not sure are justifiable, including the key assumption that demand for shark meat or shark fins is the same that it was 25 years ago and at-sea regulations governing supply are the biggest thing that has changed. Additionally, it assumes that any given country is approximately as important for the global meat trade as any other, which we know is not really the case as a handful of countries dominate the trade.

In the discussion: The authors write "Our results better support an alternative hypothesis that shark finning regulations disincentivize some countries from retaining sharks altogether and instead turn to foreign-sourced shark products to meet local demand" I believe you should stress this in the abstract, because it's not clear as written, and the abstract is the part that most people will read.

In the discussion, you note the likely possibility that other global regulations like CITES-which affect countries with anti-finning regulations and those without anti-finning regulations equally- may have had a big impact here on the shark product trade. I agree that this is plausible, but believe it should be stressed in the abstract as a likely alternative explanation. Most people will only read the abstract, and as written it creates a false impression about what happened here.

I would like to see some discussion of why the authors are confident in their results and interpretation despite a lack of statistical significance. They're making a potentially quite impactful claim, that a suite of conservation policy tools that the environmental community lobbied hard for caused some unexpected environmental harms. Strong claims require strong evidence and a lack of significance, along with the plausible alternative explanations proposed here, do not necessarily constitute strong evidence.

Finally, I would really encourage the authors to have a detailed, thorough, and actionable so what now what section. If these long lobbied for policies cause harm, what should policymakers do instead?

Overall, the authors have done some important work here, and I believe it has the potential to strengthen global conservation regulations. But there is serious potential for misunderstanding as written, and given the importance of this topic, it is vitally important to be clear about what you found, did not find, and might have found but we can't tell because of data limitations.

(Remarks on code availability)

Reviewer #4

(Remarks to the Author)
This paper employs network analysis and difference in differences to analyze the influence of shark finning regulations on the live-weight equivalent of trade and consumption of shark products. The paper utilized the Aquatic Resource Trade in Species (ARTIS) database and a mass-balance approach to estimate the live-weight trade of shark meat (excluding all shark co-products) in all shark species (excluding rays and any products reported as "elasmobranchii"). The authors indicate that their findings provide the first global shark trade and consumption analysis that isolates the effects that shark finning regulations on both the live-weight equivalent of shark products that are traded and consumed, and the prevailing trade patterns for shark products. The authors acknowledge that while their results are not statistically significant, their study underscores the need for more careful investigations of impacts from regulation on trade flows for vulnerable species. I found the approach adopted by the authors interesting, particularly when considering that most studies on this subject tend to focus on national/regional contexts with too few looking at the macro level.

I found the use of network analysis in this context to be interesting and appropriate to understand the global shark trade and consumption network. However, the written text regarding how network analysis is used left me with a few questions about the motivation and use of network analysis here. Network analysis was employed primarily to identify the "node strength", with it being the key metric of interest. Node strength is calculated here as the sum of edge weights. However trade data is directional, so the authors might want to consider using in and out degree strength or justify why the size of the trade is sufficient. Network analysis also provides other variables that might be of interest in this study such as different measures: density, modularity and centrality (degree, betweenness, closeness and eigenvector). Have the authors considered including other network analysis metrics which could help provide a more detailed picture of global shark trade and consumption dynamics?

On Line 349 – 352, the authors recognize that the implementation of regulation is unlikely to change fisher behavior and impact shark trade flows or consumption if country already lands whole sharks or regularly participates in shark fisheries. Would the results be different if the analysis were limited to the countries that have consistent catches or significant players in the trade of shark products? Network metrics like modularity and centrality might also contribute to the explanation of this.

Some other minor language style and errors that I would like to point out. Firstly, there were a few instances where citations were written in this format: "was conducted by [8]" (line 97); "recent research by [13] and [14]" (line 101). This reads

awkwardly with the Nature referencing style. Secondly, lines 417, 418, and 420, mention “FAA” regulations. FAA is not defined. Only FNA and FCR acronyms are defined in the manuscript.

(Remarks on code availability)

Version 1:

Reviewer comments:

Reviewer #1

(Remarks to the Author)
We appreciate the substantial revisions and re-analyses that the authors undertook in response to the reviewer comments. Although we did note their efforts to simply terminology and streamline the paper, it is still extremely convoluted and difficult to follow. Ultimately, our earlier concerns are unresolved despite these efforts. Their reported results do not align with the conclusions. Specifically, the authors report that in countries with anti-finning regulations, exports decreased (after 7 years of no change), imports changed a negligible amount, and domestic consumption increased (after 7 years of a decline). Despite their own reported DID results, they use the node strength/network analyses to come to divergent conclusions: that countries with shark finning regulations contributed less to exports and became more reliant on imports. They conclude that L513 " reported domestic shark landings likely decreased over time after finning regulations were adopted and countries with regulations instead turned to imported shark". This is contradicted by their own analyses of ARTIS data/DID analyses. They further speculate on changes in shark discard rates which while extremely important are not captured the ARTIS database and thus should not be part of their conclusions. Domestic consumptions has increased which could mean that shark discard rates have declined but that could be a false assumption as well. This method of analysis does not consider or use discard data (see James et al 2015, doi:10.1017/S0376892915000168)

We and the other reviewers were unanimous that while this paper can help understand how shark trade (imports, exports, domestic consumption) has changed over time, the framing of the paper as
"The impact of shark finning regulations on the trade of shark products" is not appropriate or robust.

(Remarks on code availability)
The code is accessible

Reviewer #2

(Remarks to the Author)
I co-reviewed this manuscript with one of the reviewers who provided the listed reports. This is part of the Nature Communications initiative to facilitate training in peer review and to provide appropriate recognition for Early Career Researchers who co-review manuscripts.

(Remarks on code availability)

Reviewer #3

(Remarks to the Author)
The authors have thoroughly and thoughtfully responded to my suggestions from the last version of the manuscript, and from what I can see they also appear to have done the same for other comments from other referees. I am now comfortable endorsing publication, and I congratulate the authors on a timely, significant, and well-done analysis.

(Remarks on code availability)
N/A

Reviewer #4

(Remarks to the Author)
The authors have addressed most of the comments raised in the previous version, and the manuscript has improved substantially in clarity and structure. The revisions have strengthened both the methodological reasoning and the presentation of results.

Some minor comments are listed below:
Line 193: although not grammatically incorrect, please consider removing the "a" in "We used a network analysis ...".

Lines 292-297: This sentence is long and may take a few reads to understand it. The authors should consider expressing it more concisely and clearly, or alternatively dividing it into shorter sentences.

Regarding the earlier commented part on in/out-degree, this was addressed and results were presented accordingly. However, some parts remain a little confusing when reading through and they may require a few reads to understand, especially for those less familiar with network analysis. For example, in the introduction, the authors referred to the trade activities as import and exports. But later, in sections 2.2-2.3, when reporting the results for the network, the authors repeatedly used “in-degree” and “out-degree”. It could be expressed more clearly that the in-degree network and out-degree network correspond to imports and exports, respectively. Something more alike Section 3 line 367-379 might be easier for readers to understand.

(Remarks on code availability)

Version 2:

Reviewer comments:

Reviewer #4

(Remarks to the Author)
Thank you for your careful revision of the manuscript. I have no further comments.

(Remarks on code availability)

Open Access This Peer Review File is licensed under a Creative Commons Attribution 4.0 International License, which permits use, sharing, adaptation, distribution and reproduction in any medium or format, as long as you give appropriate credit to the original author(s) and the source, provide a link to the Creative Commons license, and indicate if changes were made.
In cases where reviewers are anonymous, credit should be given to 'Anonymous Referee' and the source.
The images or other third party material in this Peer Review File are included in the article’s Creative Commons license, unless indicated otherwise in a credit line to the material. If material is not included in the article’s Creative Commons license and your intended use is not permitted by statutory regulation or exceeds the permitted use, you will need to obtain permission directly from the copyright holder.
To view a copy of this license, visit https://creativecommons.org/licenses/by/4.0/

RESPONSE TO REVIEWERS
"The impact of shark finning regulations on the trade of shark products"
Manuscript number: NCOMMS-25-60414
January 14, 2025

Note to all Reviewers
Thank you for your thoughtful review of our manuscript. Before we address your specific concerns point-by-point below, we would like to flag that we have updated the ARTIS database to the new version 1.2 which includes relevant updates to the consumption estimates. The updated consumption estimate in the database passes a much higher quality assurance test to ensure that all production is essentially exactly consumed by someone. These changes have had a minor impact on our analysis, the overall results and conclusions have not changed through some of the specific results of the summary of global shark product trade and the treatment effect estimates have changed.

Reviewer 1
The main concerns from reviewer #1 were that the manuscript (1) currently conflates efforts to address shark finning with efforts to curb overall shark mortality, (2) doesn't adequately discuss the other plausible drivers of increased shark landings and trade, and (3) underemphasizes the utility of the ARTIS data and how it can advance understanding of global shark trade dynamics rather than just confirming rising mortality. We have substantially revised the manuscript to address these concerns. We first provide a summary of major changes and then provide a detailed point-by-point response to each comment. A marked version of the manuscript is included in the uploaded documents which shows all additions in red text and all deletions as strikethroughs.

Summary of major changes:

1. We have substantially revised the framing of the manuscript to focus on the dynamics between regulatory measures and trade, using shark finning regulations as an example for how we can understand these linkages and the policy implications. The Abstract, Introduction and Discussion have been revised to reflect this reframe, as well as to explicitly acknowledge the suite of regulatory approaches that are in use to address shark mortality. These changes have helped to set clearer motivation for the analysis, which has greatly improved the readability and potential impact of our manuscript.
2. We have added text to further clarify the difference-in-difference methodology and how we isolate the effect of shark finning regulations alone. We also included an additional robustness check controlling explicitly for per-capita income and population changes which we reference in the results and include in the Supplementary Information.
3. In reframing the manuscript we have emphasized how ARTIS has linked shark landings and shark trade and elevated the research opportunities that emerge because of this,

including identifying drivers of shark mortality. This has greatly improved the strength of our manuscript.

Point-by-point response

This was a very well written, well organized, and well executed study that attempted to answer the extremely challenging question of what effect shark finning regulations have had on shark landings, trade (import and export) and consumption.

Thank you for your positive feedback on the manuscript.

The fundamental challenge to this analysis is that there have not been global efforts to curb shark mortality as stated in L78, there have been global efforts to curb shark finning. Those are not synonymous. Although shark fins have historically been the most valuable due to high demand from Asian markets, there were robust shark markets in the top shark consuming and trade countries (quartile 1 in this analysis) for shark products before the shark fin ban and those markets have grown. That growth could be related to shark fin regulations but it is just as likely that these changes are due to increased demand for seafood, growth of shark markets, or population growth (similar to patterns found in Brazil, Pincinato et al).

We appreciate the importance of not conflating efforts to eliminate shark finning with those aimed at reducing mortality. We have addressed this in the introduction and framing of the manuscript. Additionally, we have clarified within the description of the methodology how we isolate the effect of shark finning regulations alone and control for other driving forces such as increased seafood demand or population growth. The specific changes are described in more detail below.

Introduction

We have significantly revised the introduction, reproduced below, to reframe the purpose of the manuscript. We now focus on how regulations around fishing activity (like a finning regulation) could influence trade and that understanding these dynamics is critical to identifying drivers of shark mortality. Shark finning regulations are widespread, cover all shark species, and have the potential to influence trade dynamics. As such, we use finning regulations as an example to demonstrate how the ARTIS database can enable new research into linkages between regulations implemented on the water and trade that previously has not been possible.

Methodology

We agree that there are multiple plausible reasons for the growth of shark meat markets. One of the key objectives of this study is understanding if part of that growth was driven by shark finning regulations, as opposed to demand for shark meat, population growth, or other means. Our empirical strategy is designed to address exactly the potential omitted variable bias concern the reviewer raises - *"how does the approach separate the impact of finning regulations from*

other market dynamics?". A central advantage of the staggered Difference-in-Differences framework is that it isolates the *relative* change in outcomes for countries that adopt finning regulations compared with those that do not adopt them at that time, after absorbing all global movements in shark markets and shared economic drivers. Specifically:

1. **Our DID specification absorbs global shocks and shared market trends.** Year fixed effects remove all worldwide annual fluctuations—such as global seafood demand growth, international meat-price cycles, increases in shark meat acceptance, or global conservation campaigns—that may simultaneously affect all countries' shark meat markets. Additionally, we include region-specific linear trends, which further absorb systematic differences in long-run growth trajectories of shark meat markets across major world regions. Together, these two components control for the kinds of widespread economic, demographic, or market-expansion forces highlighted by the reviewer and enable us to isolate the impacts of finning regulations alone.
2. **Consistent estimation does not require controlling for every potentially relevant variable.** Under standard DID identification, unbiased treatment effects require only that unobserved factors influencing shark-product trade and consumption do **not** drive *the timing* of a country's decision to adopt a finning regulation. Put differently, as long as anticipated increases in shark meat demand, seafood consumption, or population growth did not themselves *cause* countries to adopt finning regulations earlier or later, the parallel-trends assumption holds and the policy effect is consistently identified.

We now clarify these points explicitly at the beginning of the results and in the methods:

Lines 254-257: By including global and regional fixed effects, which absorb time-varying global or regional market forces such as demand growth or demographic change, the DiD models are able to isolate the change in trade and domestic consumption attributable to finning regulations from broader market forces.

Lines 654-657: DiD is common in policy evaluation because it helps to isolate the effect of a specific regulation from other global or country-level changes. By including global and regional fixed effects, the DiD models isolate the change in trade and domestic consumption of shark products attributable to finning regulations from broader market forces.

In addition, we have expanded our robustness analysis by adding specifications to the DiD models that explicitly control for population size and per-capita income, both of which proxy for long-run demand shifts in seafood and animal protein. These additional controls do not materially change the estimated treatment effects, providing further evidence that our results are not driven by omitted demand trends. We include these results in Table S10, reproduced below.

Table S10: Aggregated ATT estimates across trade outcomes after additional controls for population and per capita gross domestic product. All models also include country (iso3c) and year fixed effects and control for the World Bank Governance Index and region-specific linear trends. Standard errors are clustered at the country level.

Model:	Exports (1)	Consumption (2)	Imports (3)	In-degree strength (4)	Out-degree strength (5)	In-degree centrality (6)	Out-degree centrality (7)
Variables							
ATT	-300.4	-1,851.4*	174.8	127.7	-300.2	0.0007	0.002
	(361.9)	(1,017.9)	(252.5)	(235.2)	(361.8)	(0.003)	(0.007)
Fixed-effects							
iso3c	Yes	Yes	Yes	Yes	Yes	Yes	Yes
year	Yes	Yes	Yes	Yes	Yes	Yes	Yes
Fit statistics							
Observations	1,008	1,092	1,064	1,064	1,008	1,064	1,008
R^2	0.95900	0.87945	0.94607	0.93489	0.95899	0.96667	0.95436
Within R^2	0.27599	0.10747	0.27534	0.22940	0.27608	0.21050	0.30123

Clustered (iso3c) standard-errors in parentheses
*Signif. Codes: ***: 0.01, **: 0.05, *: 0.1*

While I appreciate the global and comprehensive nature of the network analysis of trade and the DiD , the most compelling element of this analysis is the innovative use of ARTIS as a means of tracking shark production, which ultimately is an indicator of shark landings and catch, which I think is what you mean when you say "providing a direct link between trade flows and ecological research" [L136]
We agree that the ARTIS database is compelling and have substantially revised the introduction to elevate the importance of this dataset and the research opportunities that it enables (also see comment below). The ARTIS database links production to trade, which can be either from wild capture or aquaculture. In the case of sharks, all the production data are from wild capture fisheries so we have also clarified in the text that ARTIS links shark landings to trade flows.

Lines 139-143: By estimating the live-weight equivalent of relevant primary products (i.e., the total weight of the species that needed to be caught to produce the reported weight of the traded product), ARTIS bridges the gap between production and trade data, enabling estimates of domestic consumption and providing a direct link between shark landings and trade flows.

This analytical framework is powerful and has much to offer but in it's current form, the manuscript feels somewhat of a missed opportunity. The findings confirm what Worm et al 2024 found - global shark mortality continues to rise. However, what's exciting is that your global trade flows and trends of production (domestic consumption, domestic export, international imports) may be critical to identifying the drivers of the global shark markets and identify conditions of more sustainable shark consumption.
We appreciate this thoughtful comment to identify the manuscript's real strengths and ensure those are accurately communicated. We have substantially revised the framing of the

manuscript to focus more broadly on the connections between regulatory interventions and trade, highlighting that we have little understanding of these dynamics which are critical to understanding drivers of global shark mortality and designing effective policies. We elevate the contribution of ARTIS in linking shark landings and shark trade, which provides opportunities for the first time to test these relationships. We use shark finning regulations as an illustration of the power of the ARTIS database and provide a framework that can be used to assess the impacts of policies on global markets for shark meat but also extended to other traded aquatic species. Additionally, we have revised the discussion to highlight that this work is a stepping stone toward a better understanding of the drivers of shark mortality as well its broader applicability to other vulnerable traded marine species. Lastly, we have significantly revised the Abstract to elevate the main findings and contributions of our work.

Specific line comments

Line 81: Although you indicate what artificially attached fins are in the methods, the reader won't see that until the end of the paper. I suggest just adding a short description of what this means.

We have added a brief explanation of fins artificially attached to the text in the introduction, it now reads:

Lines 90-93 : Shark finning regulations were widely implemented to eliminate this wasteful fishing practice, requiring fins to be naturally attached (FNA) or artificially attached (FAA, e.g., with wires or ropes) to carcasses onboard; or landed in a prescribed fin-to-carcass ratio (FCR).

Line 139 - 165: This is a rather long paragraph. I suggest splitting it into two or modifying some parts to make it more streamlined. For example I think the description of node strength "156 - 160" can be moved to the results section, where you get into the network analysis and analyze changes to the network. As this format for this journal has the results coming before the methods I think this should be appropriate.

We have removed the description of node strength as suggested, which is now included in the text for the network analysis results and has been updated to in-degree and out-degree strength. Additionally, we have simplified the language around imports, exports, and consumption to streamline this paragraph and include full definitions in Table S1. The updated paragraph now reads:

Lines 144-157: Here, we leverage the ARTIS database, alongside causal inference techniques, to evaluate the effect of shark finning regulations on global markets for shark products from 2007-2020. We first provide a brief overview of the global trade of shark products. We then narrow our analysis to evaluate all imported, exported, and domestically consumed shark products (metric tons, live-weight equivalent; see Table S1 for full definitions). Next, we use a network analysis to understand the connectivity of global shark trade and understand the

relative importance of participating countries. We finally analyze whether finning regulations in participating countries have a causal effect on their: 1) volumes of traded shark products (imported and exported); 2) apparent domestic consumption of shark products; and 3) overall importance in the global trade network. Our results may serve as a foundation to better understand the impact of regulatory interventions on global shark trade. Furthermore, the methodological approach developed in this paper and the underlying ARTIS database have broad relevance to analyses of global wildlife trade, a major threat to biodiversity worldwide.

Line 173 - 174 & throughout: Make sure all tables and figures (in the main doc and supplement) are ordered by appearance. I found that they are currently ordered as if this paper was structured in the more conventional format where the methods come after the introduction.
We have restructured the Supplementary Information and ensured that the order in which figures and tables are referenced in text is the order they appear in the supplement. We have also confirmed that the numbering and order matches for the main text figures and tables.

Line 203: Was this analysis conducted on an annual basis? Figure 4 presents results for three different years, and I think it would be helpful if this were stated more explicitly in the text.
We have clarified that the network analysis was conducted annually and network metrics (in- and out-degree strength and in- and out-degree centrality) are calculated for each country in each year. We have also made changes to the Supplementary Information and now replace some of the larger tables (also see comments below) with Supplementary Data. The annual country-level results for volumes of shark products imported, exported, domestically consumed, and the in- and out-degree strength and centrality are now included in Supplementary Data 2 and described in the accompanying Description of Additional Supplementary Files. The results section now reads:

Lines 194-199: We used a network analysis to explore patterns of connectivity between shark-trading nations. In each year, we evaluated the in- and out-degree strength and normalized in- and out-degree centrality of each country (see Table S1 for full definitions). Annual network analyses metrics for countries under different finning regulations are shown in Figures S4-S7 and annual statistics by country can be found in Supplementary Data 2.

Additionally, we clarified that the three years of data shown in Figure 4 (now Figure 3), are samples of the full time series of data, both in the Results text and in the figure caption.

Lines 209-211: Finally, we assess a visual representation of the largest annual trade flows at three check-points within the dataset: 2007, 2014, and 2020 (Figure 3).

Figure 3. Trade flows of shark products in selected years (2007, 2014, and 2020). Light, thin gray lines represent all trade flows and the darker, thicker lines represent the top 10 trade flows each year. Arrows designate the direction of flow, starting in the exporting country and pointing towards the importing country and are weighted by total amount traded (mt). Shark fishing and finning regulations that were already adopted in each year are colored according to regulation type, with darker colors representing stronger regulations (Protected - sharks are fully protected from fishing; FNA - fins naturally attached; FCR - fin to carcass ratio; FRU - unspecified finning regulations). Trade flows for all years can be found in Figure S10.

Lastly, we have revised the methods to ensure that it is clear the network analysis and network parameters were calculated annually, which now reads:

Lines 570-580: To understand whether differences in trade flows occurred after shark finning regulations were adopted, we ran a directed, weighted network analysis for each year of data. Network analyses have frequently been used in previous research analyzing global trade flows [27–29]. In our network analysis, each country was modeled as a node, and the trade transaction (the live-weight equivalent of shark products traded from one country to another) was modeled as an edge. Since the trade between countries is not always reciprocal, the network was modeled as a directed network, meaning the direction of trade flows (e.g., from A to B) is accounted for. Edges were weighted by the volume of shark products traded from one country to the other. For each node, in each year, we calculated in-degree and out-degree strength and in-degree and out-degree centrality (see Table S1).

Line 290 - 292: I find it difficult to interpret the result that, based on the leave-one-out approach, Argentina was identified as the largest contributor to the DiD model results for node strength. It would be helpful if the authors could provide additional text to contextualize what this finding means, as I did not see such explanation in the results or discussion sections. Without this context, it is unclear whether Argentina represents a meaningful driver of the observed patterns or simply functions as an outlier that disproportionately influences the results.

The leave-one-out analysis is a sensitivity analysis, to assess whether our results are driven by a small number of countries. How influential a country is to the model results is calculated using DFBETAs, a value that measures how much the treatment effect changes when one country is removed. Updating the ARTIS database did impact the reported results for the leave-one-out analysis. Guinea had the highest DFBETAS score for exports, out-degree strength, and in-degree centrality, Indonesia for consumption, Uruguay for imports and in-degree strength, and Taiwan for out-degree centrality. These results indicate that these countries influence the magnitude of the estimated treatment effect. However, excluding these countries from the analysis does not alter the sign or statistical significance of the estimated treatment effect, and all leave-one-out estimates remain qualitatively consistent with the full sample results. This

indicates that these countries affect the coefficient scale but do not change the overall conclusions. Taiwan, Indonesia, and Uruguay all play central roles in global shark product trade and are among the top contributing countries to domestic consumption and imports. Their influence reflects economic relevance rather than outliers driving the qualitative interpretation. Guinea on the other hand had reported exports decline to zero after implementation of regulations, which makes Guinea an important country for interpreting the DiD analysis results even though the volume of exports is not a statistical outlier.

We have updated the results section to include a brief explanation of the leave-one-out analysis proceeding the DiD results and an interpretation of the results. Additionally, we have updated the description of the DFBETAS calculation and the leave-one-out analysis in the methods. We also include the top five countries for each model along with the ATT estimates of the leave-one-out analysis and the DFBETAS score in Table S11 (reproduced below).

Lines 282-286: Finally, we conducted a sensitivity analysis using a "leave-one-out" approach to determine the effect that individual countries had on the model results. Each country's influence over the results was calculated using DFBETAS (see Table S1); countries were then iteratively removed from the analysis and the treatment effect was recalculated.

Lines 317-329: The "leave-one-out" analysis revealed Guinea exerted the strongest influence on exports (DFBETAS = 0.74), Indonesia exerted the strongest influence on domestic consumption (DFBETAS = 0.98) results and Uruguay was the most influential country for import results (DFBETAS = 0.48). Exports in Guinea declined to zero reported exports after the implementation of finning regulations, making it a high influence country for the DiD trends even though the volume of exports overall is not a statistical outlier. While Indonesia and Uruguay are both top contributing countries to domestic consumption and imports, respectively. All leave-one-out estimates had the same sign and significance as the full-sample results indicating that influential countries affect the size of the coefficients but do not change overall trends, reinforcing confidence in the robustness of our findings (Table S11).

Lines 698-711: We assessed the influence of individual countries on the estimated treatment effect using DFBETAS, which measures how much the ATT changes when one country is removed, expressed in standard deviation units:

$$DFBETAS_i = \frac{DFBETA_i}{SE\ (\widehat{Att}_{-i})} = \frac{\widehat{Att} - \widehat{Att}_{-i}}{SE\ (\widehat{Att}_{-i})}$$

A higher absolute DFBETAS indicates greater influence; generally values above 0.2 have the potential to meaningfully alter the results if excluded [38]. DFBETAS was used to identify countries that had the greatest influence over the model results. To evaluate sensitivity, we performed a "leave-one-out" analysis, which iteratively removed one country and recalculated

the ATT. We then compare the sign and significance of these estimates to the full model to determine whether influential countries affected the overall conclusions.

Line 388: True. I think adding in CITES analysis could help support this more but I understand that could be future work.

We appreciate this suggestion that incorporating CITES listings could enrich the analysis. However, as a policy CITES is not well suited to the difference-in-difference approach. Our study examines national finning regulations and their effects on country-level aggregate trade and consumption. The difference-in-difference framework developed here requires staggered adoption of a policy across countries to create treatment and control groups. By contrast, CITES listings operate at the global scale and all countries are affected at the same time when a species is listed. As such, CITES is not suitable for the DID approach used in this study. Evaluating the before–after effects of CITES listings will require a different methodological approach and constitutes a promising direction for future research. We have added the following text to the discussion to make this explicit:

Lines 427-432: The DiD approach used in this study requires the staggered adoption of a policy across countries over time. Since CITES listings operate at the global scale and all countries are affected at the same time when a species is listed, the DiD approach used here is not suitable. Evaluating the impact of CITES listings would require a different methodological approach and constitutes a promising direction for future research.

Line 411: I suggest adding FAA for consistency

We have added FAA, the relevant text now reads:

Lines 504-511: Regulations are categorized by strongest to weakest regulation as: 1) shark sanctuaries where all shark fishing is prohibited; 2) fins naturally attached (FNA), where all landed sharks must be whole with their fins attached; 3) fins artificially attached (FAA), where caught sharks may have their fins removed for storage, but re-attached via ropes or wires prior to landing; 4) fin-to-carcass ratio (FCR), where shark fins must be landed alongside shark carcasses in a defined ratio; or 5) unspecified finning regulations where there is some form of prohibition but its definition is not clearly documented.

Lines 428 - 433: While these sentences are well written I found myself having to reread it a couple of times to grasp what it conveys. I think a very simple equation may help readers clearly and intuitively understand what is the main metric ARTIS calculates. For example something like:

$$\text{Exports} = \text{Domestic Production} + \text{Imports} - \text{Domestic Consumption} - \text{Processing Losses}$$

The full ARTIS model involves more steps than just the mass balance problem, and the mass-balance equation is slightly more complicated as it involves matrices representing the proportions of production entering each product and the transformation among imported products, with the matrices accounting for processing loss constants. The elements of the matrices are estimated using optimization and the solutions are used to disaggregate the trade data. Given that it would require a fair amount of methods text to fully repeat the methods from the original paper, we revised the Methods section to include a more detailed description of the modeling approach underlying the ARTIS database and refer the reader to the main manuscript for the specific details of the equations. The revised method now reads:

Lines 523-532: For each country, ARTIS modeled (1) the conversion of wild and farmed production into traded commodities, (2) proportion of imported commodities that were processed and exported, and (3) the apparent domestic consumption of each commodity. These estimates were then used to estimate each country's exports sourced from domestic production versus imports and link re-exported products to their original product form and source country based on the mass-balance solutions and bilateral trade data. Once producing countries were estimated for all trade flows, the producing country's species mix within each originally exported code was applied to disaggregate trade into species/species groups. Additional details of the methodology and mass balance calculations are provided in Gephart et al. [18].

Lines 465 - 468: Again not a poorly written sentence but rather long and windy due to the nature of what's being explained. I think breaking it up into two sentences would help make it more interpretable.
We have broken this sentence up to improve readability. In addition, we have simplified the language throughout the manuscript to just domestic consumption, imports, and exports and include the full definitions in Table S1.

Lines 561-565: Imports included shark products that were directly exported from a producing country and products that were re-exported via intermediary processing countries (see Table S1 for full definitions). Shark products originally sourced in the importing country that were exported for processing and then re-imported were excluded.

Lines 512: Any reasoning for 2 years? Does is have to do with the lag seen with CITES
Since the DiD analysis conducts before-after comparisons for countries in the treated group, sufficient data are required both before and after a regulation is adopted. Those countries with regulations in place before 2007 are excluded because they don’t have any data pre-regulation.

Requiring at least two years of data pre-adoption is standard practice in DiD analysis and is considered the minimum amount of data required to meaningfully assess the parallel trends

assumption, which is central to DiD credibility. With only one pre-treatment observation, that year is absorbed by the country fixed effect and provides no information about pre-trends, making it impossible to test whether treated and control countries were evolving similarly before regulation. As emphasized in Angrist & Pischke (2008), inspecting pre-treatment trajectories is an important diagnostic for DiD validity, and such diagnostics require multiple pre-event periods (preferably over 2) for transparent DiD pre-trend testing**.** Additionally, having more than one pre-period to average over allows smoothing the influence of year-specific shocks in the individual pre-event period, providing a more stable baseline for before–after comparisons for the adopter and improving the precision of event-study estimation. While having additional pre-period observations is always beneficial, the two-year minimum drops only three adopting countries from the estimation sample while ensuring that early adopters contribute at least one estimable pre-trend data point.

For clarification, we have added the following text to the methods:
Lines 661-664: The parallel trend assumption was visually assessed using an event study plot which shows how the trends in the treatment and control groups evolve over time. Small absolute values indicate small differences between the two groups satisfying the parallel trend assumption, while larger absolute values indicate large differences that do not satisfy the assumption.

Lines 669-672: At least two years of data before the adoption of a regulation is required to ensure credible estimate of the pre-treatment trend [37]. Therefore, we restricted the sample of the treated group to countries that adopted their first regulation in 2009 or later.

Reference 37: Angrist, J. D. & Pischke, J.-S. *Mostly Harmless Econometrics: An Empiricist's Companion*. (Princeton University Press, 2009). 10.2307/j.ctvcm4j72.

Table and Figures
Table S8: The supplement has several very large tables that span several pages. This is one that I suggest can be converted into a figure to make it more easily interpreted than spanning 3 pages.
Thank you for this suggestion. We have done a major revision to the Supplementary Information to reduce the number of large tables and based on additional publication guidance have opted to include this information along with the information in the previous Table S7 as a supplemental dataset (Supplementary Data S2). This dataset includes the volume of exports, imports, and domestic consumption of shark products, the in- and out-degree strength and in- and out-degree centrality values for each country in each year. We have also removed Table S5 (list of shark species included in the analysis) and include it as Supplementary Data S1. A description of these data files is included in the "Description of Additional Supplementary Files".

Figure S1: I am finding it hard to interpret what each quantile exactly means. Is quantile 1 the countries with the largest baselines of trade volumes or is it quantile 4? I suggest providing the ranges of these for each quantile bin in the figure caption or in the figure itself.

We thank the reviewer for pointing this out — we agree that the interpretation of the quantile groups should be clearer. We have replaced the figure with a supplementary table (Table S9) to match the results format of the other robustness checks. We now included the quantile ranges for each model within the table directly to facilitate comparison and make it easier to interpret. Additionally, we have expanded the table captions to clarify that the stratification is based on the volumes traded and network metrics calculated in 2007 and that the first quantile reflects the highest baseline values with progressively smaller values shown in the second through fourth quantiles.

Table S8: Aggregated ATT estimates across outcomes, by baseline quantile. Quantiles are based on 2007 baseline trading volumes and network metrics with the first quantile containing highest baseline trading values. Quantile ranges report the minimum–maximum baseline outcome values within each quantile. The fourth quantile contains the lowest baseline trading volumes (all equal to zero in 2007), though countries in this group are active in shark product trade in other years included in the analysis. For network outcomes (in-/out-degree strength and centrality), there are no treated units in Quantile 4 and corresponding DiD estimates are therefore not available. All models include country (iso3c) and year fixed effects and control for the World Bank Governance Index and region-specific linear trends. Standard errors are clustered at the country level.

	Exports	Consumption	Imports	In-degree strength	Out-degree strength	In-degree centrality	Out-degree centrality
**Quantile 1**							
ATT	-145.7***	-114.5***	-297.3**	-117.6*	-145.7***	-0.010	0.014*
	(32.2)	(34.3)	(126.1)	(57.1)	(32.2)	(0.006)	(0.007)
Quantile ranges	311-21954	578-42327	169-15084	117-12373	311-21951	0.05-0.27	0.09-0.69
Observations	280	294	266	252	280	252	266
**Quantile 2**							
ATT	-768.1	254.9	-128.8*	-159.8*	-767.2	-0.002	-0.001
	(1,096.2)	(203.5)	(61.2)	(81.5)	(1,095.4)	(0.006)	(0.045)
Quantile ranges	2-286	11-547	10-167	9-113	2-286	0.02-0.05	0.02-0.09
Observations	196	294	238	294	196	322	210
**Quantile 3**							
ATT	-393.2*	-7,920.4***	70.0	-62.9	-153.8	0.005	-0.004
	(211.3)	(2,151.4)	(463.8)	(453.1)	(197.6)	(0.008)	(0.003)
Quantile ranges	0-2	0-11	0-9	0-8	0-2	0-0.02	0-0.02
Observations	294	252	406	378	308	350	308
**Quantile 4**							
ATT	-73.7	10.5	7.26				
	(109.8)	(153.3)	(6.28)				
Quantile ranges	0-0	0-0	0-0				
Observations	252	266	154				

Clustered (iso3c) standard errors are in parentheses.
Significance codes: *** $p < 0.01$, ** $p < 0.05$, * $p < 0.1$.

Figures S3 - 13 & S19 - 24: I understand why the authors constructed the figures this way to show each country's time series. However, it gets overwhelming for the reader and hard for them to visually see the changes. I suggest the authors try and collapse them into just a couple of plots which could be binned by countries that adopted regulation vs ones that don't or by continent, so that readers can see the overall trend across the countries and are better able to compare across countries.

We appreciate this suggestion and have condensed the time series figures down breaking it out by type of shark finning regulations. The plot for each metric includes four panels: Fins-naturally-attached, Fin-to-carcass ratio, Finning regulations unspecified, and No regulations. In each panel all countries with that regulation are shown as individual lines. The top 5 countries under each type of regulation are colored and labeled and all other countries are shown as unlabeled gray lines. Figure S1 is replicated below, all other replacement figures follow the same format.

Previous Figures S3-S5 (exports) are now Figure S1, Figures S6-S8 are now Figure S3 (consumption), Figures S9-13 (imports) are now Figure S2. Node strength has been replaced with in- and out-degree strength and Figure S19-23 are now Figure S4 (in-degree strength) and Figure S6 (out-degree strength). We have also added a new network metric for in- and out-degree centrality which is included as Figures S5 and S7, respectively.

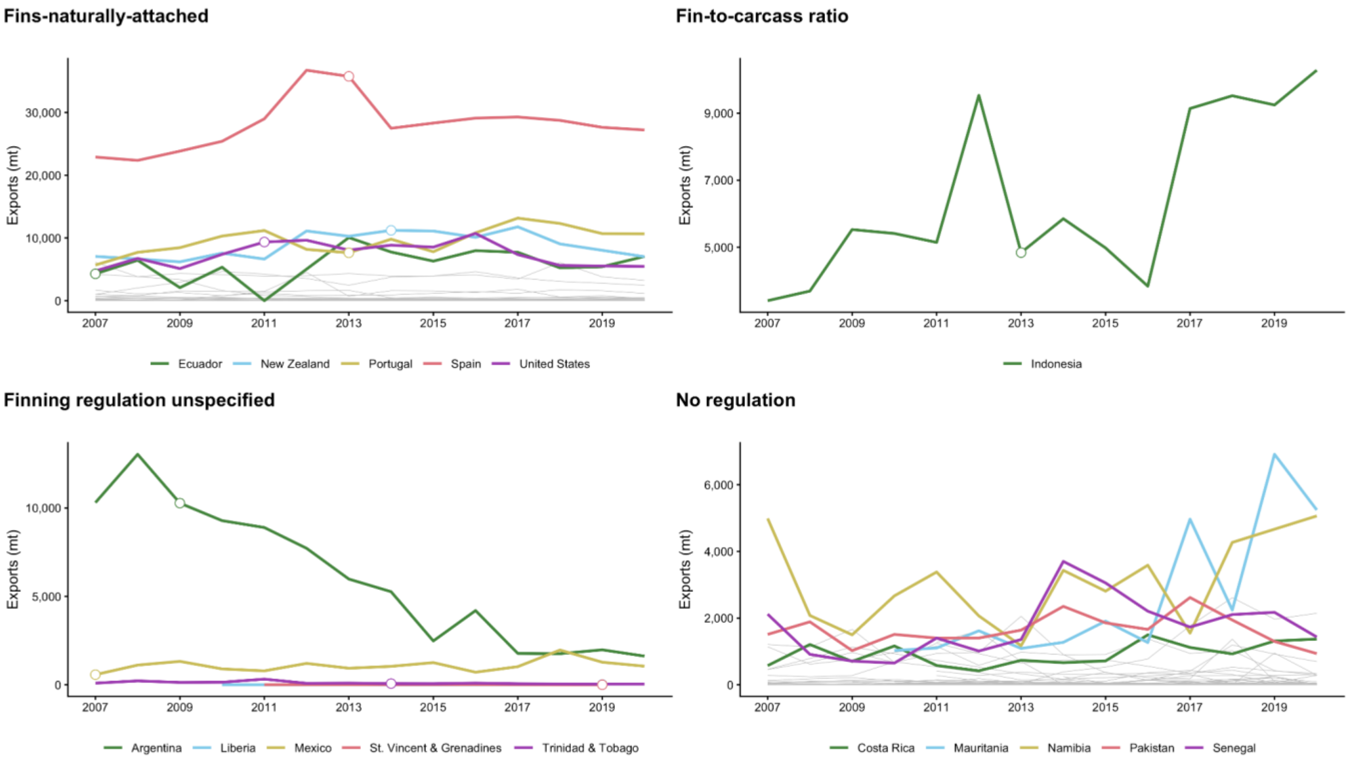

Figure S1. Time series of country-level exports of shark products (metric tons, live-weight equivalent) under fins-naturally attached (top left), fin-to-carcass ratio (top right), unspecified finning regulations (bottom left) and no regulations (bottom right). The top 5 contributing countries for each type of regulation are colored, and other countries are depicted as gray lines. For subplots in which regulations are implemented, open circles for colored lines indicate the year in which the regulation was implemented.

Figure 4: This is a great figure, however, it's rather small and hard to see. I suggest making it vertically oriented so that each map can be larger.

Thank you for this suggestion, we have updated this figure to be vertically oriented. This is now Figure 3 in the revised manuscript and is reproduced below.

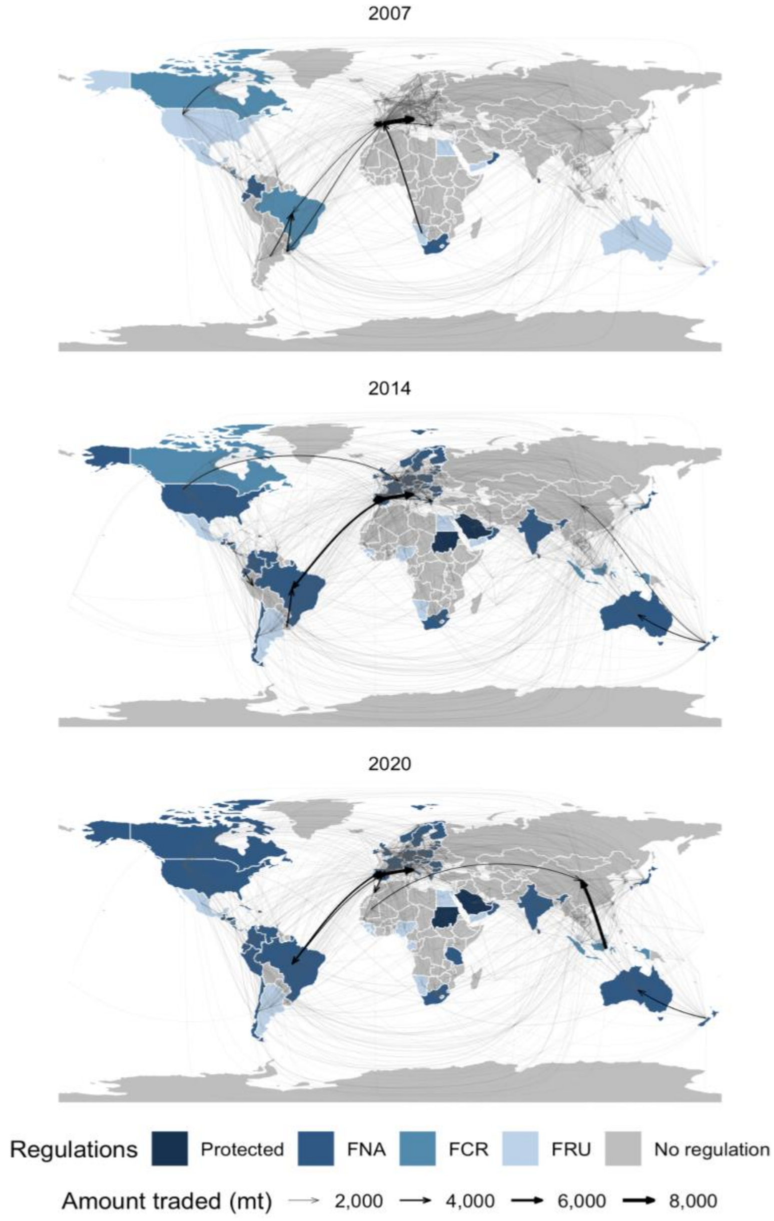

Remarks on code availability
I clicked on the link to review the code on Zenodo and it is restricted so I was unable to see the posted code.
Thank you for pointing this out. Upon acceptance we will make the link publicly available. For the revision we have included a copy of the zip file as it appears on Zenodo as an additional file in the submission.

Reviewer 2

Point-by-point response
I co-reviewed this manuscript with one of the reviewers who provided the listed reports. This is part of the Nature Communications initiative to facilitate training in peer review and to provide appropriate recognition for Early Career Researchers who co-review manuscripts.
We appreciate your time in reviewing our manuscript and your input for how to improve it. Please find our responses to points raised in the listed comments for each reviewer.

Reviewer 3
The main concerns from reviewer #3 were that (1) the manuscript frames the increase in shark meat trade as a result of shark finning regulations when there isn't a robust evidence base to support this, (2) the approach claims causality but rests on a series of assumptions that are not adequately justified or explained, and (3) the manuscript lacks a discussion of policy implications or actionable steps for policy makers. We have substantially revised the manuscript to address these concerns. We first provide a summary of major changes and then provide a detailed point-by-point response to each comment. A marked version of the manuscript is included in the uploaded documents which shows all additions in red text and all deletions as strikethroughs.

Summary of major changes:

1. We have substantially revised the framing of the manuscript to focus on the dynamics between regulatory measures and trade, using shark finning regulations as an example for how we can understand these linkages and the implications for policy. The Abstract, Introduction and Discussion have been revised to reflect this reframe, as well as to remove any text that states the growth in shark meat markets is caused by shark finning regulations. These changes have helped to set clearer motivation for the analysis, which has greatly improved the readability and potential impact of our manuscript.
2. We have revised the Results and Methods sections to expand the explanation of the causal inference techniques used and to explicitly state the assumptions of the difference-in-difference analysis.

3. We have expanded the Introduction to highlight how understanding the relationship between fisheries management policies and trade is critical to informing policy and restructured the Discussion to acknowledge the limitations of our findings and present opportunities for future research. These changes have helped to clarify the scope of the manuscript and its limitations.

Point-by-point response
The authors have performed an interesting and timely analysis of emerging shark conservation issues. The ARTIS database is an interesting dataset that seems appropriate for challenges like this. This work is potentially important and impactful, but I have some concerns about framing, analysis, and presentation that I'd like to see addressed.
Thank you for your positive and constructive feedback on the manuscript.

Sharks are some of the most threatened animals on Earth, there has been intense public interest surrounding their conservation, and some conservation measures may have had previously-unexamined unintended consequences. I agree with all of this as a basis for the analysis. I also agree that the shark meat trade has increased as the shark fin trade and shark finning has declined, and that shark finning has become more regulated.

However, some of the specific framing used to justify this analysis- that the shark meat trade has increased potentially BECAUSE shark finning has become more strictly regulated- is questionable. Indeed, one of the papers cited in the introduction to justify this claim (reference 12) explicitly tested this hypothesis and found that it wasn't really the case: "This increase may be a consequence of stricter finning regulations that has created incentives for full utilization of sharks and exposed the resource to a new source of demand… Results indicate that shark meat is not a new market in Brazil, but an old one." The other reference (reference 8) acknowledges that this may be part of the issue, but isn't the only issue: "Meanwhile, a combination of demand growth and antifinning regulations intended to encourage the full utilization of carcasses has seen the market for shark meat expand considerably."
We have significantly revised the introduction, reproduced below, to reframe the purpose of the manuscript. We now overview how regulations around fishing activity (like a finning regulation) could influence trade and highlight that understanding these dynamics is critical to identifying drivers of shark mortality. Shark finning regulations are widespread, cover all shark species, and have the potential to influence trade dynamics. As such, we use finning regulations as an example to demonstrate how the ARTIS database can enable new research into linkages between policies implemented on the water and trade that previously has not been possible.

We agree that there are multiple plausible reasons for the growth of shark meat markets and understanding if part of that growth was driven by shark finning regulations, as opposed to demand for shark meat, population growth, or other means, is one of the key objectives of this study. In the introduction we now clarify that the influence of shark finning regulations on markets could be growth of existing markets (as indicated in Pincinato et al. 2022) or expansion to new markets. We also explicitly acknowledge that economic incentives or changes in demand can influence market dynamics and have made it challenging to isolate the impact of finning regulations alone.

Additionally, our difference-in-difference specification absorbs global shocks and shared market trends. Including year fixed effects removes all worldwide annual fluctuations—such as global seafood demand growth, international meat-price cycles, increases in shark meat acceptance, or global conservation campaigns—that may simultaneously affect all countries' shark meat markets. We also include region-specific linear trends, which further absorb systematic differences in long-run growth trajectories of shark meat markets across major world regions. Together, these two components control for widespread economic, demographic, or market-expansion forces and enable us to isolate the impacts of finning regulations alone.

Lines 58-157: The rapid decline of sharks and their relatives (Class Chondrichthyes) is one of the world's most pressing biodiversity issues and can result in severe, negative impacts on ocean ecosystems [1–3]. Shark species have inherent life history characteristics that make them susceptible to abrupt population declines, including late maturation, longevity, and low fecundity. Recent studies indicate that more than one third of chondrichthyan species face possible extinction [4], driven by their over-exploitation in global fisheries [5, 6], where they are caught as unintended 'bycatch', or directly targeted to supply a growing demand for shark products such as fins, meat, and oil.

Global efforts to curb shark mortality include national, regional, and international regulations aimed at protecting sharks from fishing (e.g., through species specific retention bans or spatial shark sanctuaries), curbing unethical and wasteful fishing practices (e.g., prohibiting shark finning where the valuable fins are cut off and the body discarded), and/or regulating international trade in shark products (e.g., restrictions under the Convention on International Trade in Endangered Species of Wild Fauna and Flora [CITES] or national trade bans). Despite a rapid increase in these efforts over past decades, shark mortality remains high in most regions, and globally [7]. Addressing the deeper economic drivers of observed shark mortality trends is challenging because regulations on fishing practices can influence trade dynamics and demand for traded shark products can in turn affect fishing practices (e.g., [8, 9]). Here, quantifying the relationship between finning regulations and trade responses may offer new insight into how management decisions shape global trade and consumption patterns and, ultimately, conservation outcomes.

Country-specific shark finning regulations provide a useful case study to illustrate such linkages between management and trade flows, as they apply broadly to all shark species, are widely adopted, and have a direct connection to traded shark products associated with each country. Shark fins have historically been the most valuable shark product due to high demand from East and Southeast Asian markets [10]. Fishers who did not traditionally target sharks for meat often engaged in shark "finning", the practice of removing and retaining shark fins and discarding the carcasses (or live animal) at sea. This method allowed fishers to maintain vessel storage for primary target species, such as tuna, while also participating in the lucrative shark fin trade [11]. Shark finning regulations were widely implemented to eliminate this wasteful fishing practice, requiring fins to be naturally attached (FNA) or artificially attached (FAA, e.g., with wires or ropes) to carcasses onboard; or landed in a prescribed fin-to-carcass ratio (FCR) [7]. Since these regulations require fishers to either discard or land whole sharks, they may incur one of three possible trade-related outcomes (1) international trade of shark products increases (whole sharks are now retained, leading to development or expansion of global markets for shark products not previously retained and landed); (2) domestic consumption of shark products increases (whole sharks are retained and meat is consumed locally), or (3) there is no effect on the global volume of shark meat traded (either because fisheries originally finning sharks now discard them whole at-sea, because regulations are not properly enforced, or because their effects are masked by other market trends). Indeed, the relationship between shark finning regulations and shark trade is not entirely straightforward. The direct results of finning regulations may depend on other fishing regulations, economic incentives, or changes in market demand, making it difficult to isolate the role that shark finning [10, 12]. For example, countries that participate little in global trade may not experience any changes after regulations are implemented, whereas countries with traditional markets for shark meat, such as Brazil, may experience shifts in trade patterns [12]. Given the variety of potential outcomes, understanding the impact of finning regulations on shark trade has important implications for domestic and international policy. Higher discard rates would necessitate additional management measures that target fishing behavior in order to effectively reduce catch and mortality of discarded sharks, while growth or expansion of meat markets would require additional regulations related to the trade of whole sharks, with particular importance for vulnerable shark species.

The ability to evaluate the impact of regulatory interventions on the trade of shark products has historically been difficult due to complex global supply chains and shortcomings in global trade data. Trade data are aggregated to generic product codes that often lack meaningful taxonomic information (e.g., Harmonized System code 030420 corresponded to "frozen fish fillets" up to 2002). Trade data are also reported as product-level transactions between importing and exporting countries, without distinguishing trade through intermediate countries, while production data (i.e., fisheries landings) are reported at the species level by the producing country, generally representing the fishing vessel flag. In addition, several species can often be used to produce the same product, and one species could be used to create multiple different products. These issues have prevented accurate tracing of species-specific

shark products through the supply chain. Previous efforts to characterize global shark markets have emphasized that these reporting standards make it challenging to trace species-specific shark products through the supply chain [10, 13, 14].

To overcome the paucity of taxonomically resolved global trade data, prior research on shark markets has focused on local scales, working with government agencies to characterize the import and export of particular products to individual countries [12, 15], or by using DNA fingerprinting to properly identify species labeled as "sharks" [16, 17]. However, this approach is time-consuming and infeasible to reproduce at global scales, thereby preventing effective hypothesis testing of the effects of shark regulations on global shark trade. The newly available Aquatic Resource Trade in Species (ARTIS) database offers a promising solution to overcome critical data gaps by using a mass balance approach to reconcile production and trade data at the country-level to estimate the first species/species group disaggregated global database of aquatic product trade from 1996-2020 [18]. By estimating the live-weight equivalent of relevant primary products (i.e., the total weight of the species that needed to be caught to produce the reported weight of the traded product), ARTIS bridges the gap between production and trade data, enabling estimates of domestic consumption and providing a direct link between shark landings and trade flows [18].

Here, we leverage the ARTIS database, alongside causal inference techniques, to evaluate the effect of shark finning regulations on global markets for shark products from 2007-2020. We first provide a brief overview of the global trade of shark products. We then narrow our analysis to evaluate all imported, exported, and domestically consumed shark products (metric tons, live-weight equivalent; see Table S1 for full definitions). Next, we use a network analysis to understand the connectivity of global shark trade and understand the relative importance of participating countries. We finally analyze whether finning regulations in participating countries have a causal effect on their: 1) volumes of traded shark products (imported and exported); 2) apparent domestic consumption of shark products; and 3) overall importance in the global trade network. Our results may serve as a foundation to better understand the impact of regulatory interventions on global shark trade. Furthermore, the methodological approach developed in this paper and the underlying ARTIS database have broad relevance to analyses of global wildlife trade, a major threat to biodiversity worldwide.

I am also confused by some potentially needlessly complex terminology. Are not all exports domestically sourced by definition? Are not all imports foreign sourced by definition? Why not just say imports, exports, and local consumption?

We appreciate this comment that the terminology used takes away from the interpretability of the paper and have revised the manuscript to simplify the language. Since the ARTIS database tracks intermediary processing, not all exports are domestically sourced and not all imports are foreign sourced. Exports can be sourced from intermediary countries that did not originally catch sharks (foreign-sourced export) and countries that originally landed sharks sometimes export it for processing to another country and then re-import it as a different product (a domestically-

sourced import). Both products that are imported and domestically-sourced can be consumed. Given that we expect regulations to impact a country's domestic shark landings we focus on domestically-sourced exports and domestic consumption. However, we agree that this is unnecessarily complicated and we now refer to the terms as exports, imports, and domestic consumption throughout. We have also included a table in the supplement (Table S1, copied below) with the full and complete definitions that match those used in ARTIS to ensure a clear link between the database and the data used in this analysis.

Table S1: Complete definitions for terms used in the manuscript.

Manuscript term	Definition
Exports	All exports (in tons, live weight equivalent) where products were sourced from the same country they were exported from, refered to as domestically-sourced exports in the ARTIS database.
Imports	All products (in tons, live weight equivalent) that were sourced in a different country than the importing country, refered to as foreign-sourced imports in the ARTIS database. Products that are exported from a source country for intermediary processing and then re-imported back to the source country are excluded.
Domestic consumption	Domestic consumption of shark products (in tons, live weight equivalent) are products sourced in the same country they are landed, refered to as domestically-sourced consumption in the ARTIS database. Domestic consumption, also called apparent domestic consumption, is an estimated value in the ARTIS database it is not directly reported.
Shark products	Used to describe all shark products collectively tradedand expressed as a live weight equivalent or the total weight of individuals that would have been caught to achieve reported product weights. To avoid double counting ARTIS only tracks one primary product, for sharks this is shark meat. The live weight equivalent would also capture other parts of the shark that might enter trade (e.g., fins, liver, oil, etc.) though these are not tracked indvidually or explicitly.
In-degree strength	In network analysis, the sum of the weights of all incoming edges to a node. In the shark trade network, this is the the total amount of shark products imported to a given county (in tons, live weight equivalent).
Out-degree strength	In network analysis, the sum of the weights of all outgoing edges from a node. In the shark trade network, this is the the total amount of shark products exported from a given county (in tons, live weight equivalent).
In-degree centrality	In network analysis, the number of incoming edges to a node. In the shark trade network, this is the number of a countries a given country imports from. The normalized in-degree centrality is the number of incoming nodes relative to all possible incoming nodes.
Out-degree centrality	In network analysis, the number of outgoing edges from a node. In the shark trade network, this is the number of countries a given country exports to. The normalized out-degree centrality is the number of outgoing nodes relative to all possible outgoing nodes.

Density	A measure of network connectivity representing the proportion of actual connections in a network relative to the total possible connections. In the shark trade network, this is the number of active country-to-country trade partnerships relative to all possible trade partnerships. Low density means the network is sparse with few active country-to-country links and high density is a very connected network where most countries trade with each.
Modularity	A measure of how well a network can be clustered into distinct clusters. In the shark trade network, high modularity represents regionally clustered trade partnerships and low modularity represents a diverse global network with few distinct groups. Modularity is calculated for an undirected version of the network that considers the total volume of shark products traded but does not distinguish between imports and exports.
Treatment group	A group that receives the intervention being studied (also called a treated group). In this analysis, these are countries that implement shark finning regulations during the study period.
Control group	A group that does not receive treatment and acts as a baseline for comparison. In this analysis, these are countries without shark finning regulations during the study period.
Not-yet-treated	Units that will receive treatment later but have not yet at a given point in time. In this analysis, for a given year, these are countries that will adopt shark finning regulations during the study period but have not yet.
Treatment effect	The estimated impact of the intervention, calculated as the difference in outcomes between the treated and control groups before and after the policy. In this analysis, it represents the change in shark trade outcomes attributable to finning regulations.
Parallel trend assumption	The assumption that without treatment, treated and control groups would have followed the similar trends over time. In this analysis, it means shark trade patterns would have evolved similarly in both groups if no regulations were introduced.
DFBETAS	A diagnostic statistic that measures how much an individual observation influences regression coefficients. In this analysis, it identifies whether a single country-year observation disporportionately affects the estimated treatment effect.

The analyses used in this study also cannot really address causality, they can analyze what's happening when, but not necessarily why or in response to what. That relies on input and analysis from the researchers. The approach of treating countries with anti-finning regulations as the experimental variable and those without anti-finning regulations as control makes a lot of assumptions that I am not sure are justifiable, including the key assumption that demand for shark meat or shark fins is the same that it was 25 years ago and at-sea regulations governing supply are the biggest thing that has changed.
We thank the reviewer for raising this important point. We recognize that the causal logic behind Difference-in-Differences (DiD) may be unclear and we have revised the manuscript to explain the design more clearly.

We agree that the casual impact interpretation depends on the framework and assumptions of analysis. DiD functions like a quasi-experiment. It compares how outcomes change over time in countries that adopt finning regulations versus countries that do not, while controlling for the background changes that affect everyone (e.g., global seafood demand growth, changing consumer preferences, or global economic trends). This allows us to isolate whether regulated countries changed exporting/importing/consuming behaviors more or less than expected, given the exporting/importing/consuming behaviors of both groups before the regulation.

The simple thought experiment we are taking in the analysis is:

- If two countries showed similar trends in trade outcomes before one country adopted a finning regulation (this assumption is examined by pre-trend testing)
- and after adoption of a finning regulation one country starts to diverge from the other,
- we then infer that finning regulation—rather than global market conditions—likely played a role.

The DiD approach relies on a key assumption:

- **Parallel trends**: absent the regulation, treated and control countries would have followed similar trends in exporting/importing/consuming behaviors once we absorb common shocks and region-level trajectories.
    - The parallel trend assumption is tested as part of the analysis through visual assessment of the event study plots (Figure 4).

In addition, interpretation of the policy effects assumes that countries do not select the timing of the timing of national finning regulations by anticipated short-run changes in shark meat demand or country-specific shocks to trade volumes. This assumption is plausible in our context because national finning regulations are typically motivated by conservation and compliance

considerations rather than short-run fluctuations in shark meat demand, and because our event-study analysis shows no systematic pre-treatment responses.

Importantly, because the DiD is controlling for background changes that affect the market as a whole, it does not assume that shark-meat demand has been constant for 25 years or that all countries are equally important in the global market. The global demand growth and regional differences are absorbed by year fixed effects and region-specific trends, respectively. We have also added further controls like population and income into the robustness checks.

We also agree that the DiD approach cannot claim to identify the primary drivers for shark meat market evolution. Instead, our purpose was to tease out the impact of finning regulation changes from all background changes. In doing this, we seek to identify whether there is a detectable shift in trade or consumption associated with adopting a finning regulation, after accounting for global and regional trends. The absence of statistically significant effects suggests that finning regulations have not been a major driver of the observed growth in shark meat markets—aligning with ecological observations such as those documented for Brazil.

We hope this clearer explanation helps communicate the intent and limitations of our empirical design. We have included excerpts of the relevant changes to the manuscript's Results and Methods that clarify the approach and its limitations below.

Lines 248-268: We used a Difference-in-Difference (DiD) approach to evaluate whether shark finning regulations implemented over time have caused concomitant changes in five market outcomes for shark products: 1) exports, 2) domestic consumption, 3) imports, 4) in- or out-degree strength, and 5) in- or out-degree centrality (Tables S3-S4). DiD models compare trends before and after the adoption of finning regulations between regulated (treated) and unregulated (control) countries to determine the causal impact of regulation on each outcome. By including global and regional fixed effects, which absorb time-varying global or regional market forces such as demand growth or demographic change, the DiD models are able to isolate the change in trade and domestic consumption attributable to finning regulations from broader market forces. These models rely on the parallel trends assumption that without a regulation, treated and control countries would have followed the same trends in exporting, importing, and consuming shark products. As such, a DiD model does not directly evaluate trends over time (e.g., countries had higher exports before imposing a regulation), but rather evaluates the relative difference between control and treated countries at each time step (e.g., countries with regulations had lower exports compared to control countries in year 10). In our context, "treated" countries are those that adopted any finning regulation and "control" countries are those that have not yet adopted such regulation in a given year or never adopt such regulations (Figure S11). Treatment effects are defined as the causal impact of adopting a shark finning regulation on the outcome of interest (see Table S1 for complete definitions).

Lines 646-665: We evaluated the causal effects of shark finning regulations on various trade outcomes using a staggered difference in differences (DiD) design [36]. This design exploits the fact that countries adopted finning regulations (treatment) at different points in time, thereby creating "natural experiments". The change in shark product trade and domestic consumption in each regulating country (treated countries) before and after implementation is compared to the change in a control group of countries (controls) that had not yet or never adopted the regulation.

DiD is common in policy evaluation because it helps to isolate the effect of a specific regulation from other global or country-level changes. By including global and regional fixed effects, the DiD models isolate the change in trade and domestic consumption of shark products attributable to finning regulations from broader market forces. Under the assumptions that 1) treated and control countries would have followed parallel trends absent regulation and 2) regulation adoption only impacts the adopting country, any systematic deviation in the outcome after adoption can be interpreted as the causal effect of the policy. The parallel trend assumption was visually assessed using an event study plot which shows how the trends in the treatment and control groups evolve over time. Small absolute values indicate small differences between the two groups satisfying the parallel trend assumption, while larger absolute values indicate large differences that do not satisfy the assumption.

Additionally, it assumes that any given country is approximately as important for the global meat trade as any other, which we know is not really the case as a handful of countries dominate the trade.
We agree that the global shark meat trade is dominated by a handful of countries. The ARTIS trade data confirms this as well, showing exports as highly concentrated with just seven countries accounting for 75% of all exported shark products each year (Table 1 in main text).

As mentioned in the comment above, the difference-in-difference approach does have important assumptions, but it does not require an assumption that all countries are equally important in the global shark meat trade. This is because the method relies on within-country changes over time, compared to changes in a control group and country-specific fixed effects account for any time-invariant differences such as market size or trade share. However, countries with large trade volumes or unusual patterns can still influence the treatment effect estimates or skew the results. To address this, we conducted a sensitivity analysis using a leave-one-out approach which iteratively removed each country and recalculated the treatment effect. Importantly, the sign and significance of the results were the same whether or not the most influential countries were included, refuting the idea that our findings are driven solely by these countries and confirming their general applicability. Additionally, we completed a series of robustness checks with varying constructions of the treated and control groups including stratifying countries by baseline trading volumes and limiting countries to those that consistently land shark products.

Similar to the leave-one-out analysis, all of these varying DiD constructions yielded results that were consistent in sign and significance with our primary model results, providing further confidence that the results are not driven by one country or by trade volumes. The robustness check results are presented in Tables S5-S10 and we now include a summary of all the robustness checks in the Results, which now reads:

Lines 268-282: We also assessed the credibility of our findings through six sets of robustness checks, by 1) limiting the treatment group to only countries that adopted the strongest finning regulation (fins naturally attached), 2) limiting the control group to countries not yet regulated at the time, but which eventually adopt similar legislation, 3) limiting the treatment group to only countries that consistently land shark products, 4) stratifying the countries by baseline trade volumes of shark products, 5) differentiating species groups occupying different habitats (i.e., coastal, pelagic, deep-sea sharks), and 6) including additional controls for a countries' population size and per-capita income. Consistency between the results of the primary model and robustness checks ensure that the reported outcomes are not driven by a specific finning regulation, by countries that never implement a regulation, by countries that do not consistently trade shark products, by countries that contribute the most to the shark trade, by species groups from a particular habitat, or by a country's population and income. Detailed regression results are presented in the Supplementary Information (Tables S5-S10) and are consistent with our main results.

In the discussion: The authors write "Our results better support an alternative hypothesis that shark finning regulations disincentivize some countries from retaining sharks altogether and instead turn to foreign-sourced shark products to meet local demand" I believe you should stress this in the abstract, because it's not clear as written, and the abstract is the part that most people will read.

We appreciate the suggestion. We have revised the abstract to ensure that it clearly communicates the main result of the paper. In line with other suggestions, we have carried the reframing through to the abstract. It now focuses on the relationship between regulatory measures (like a finning regulation) and trade and highlights how data gaps have prevented any understanding of this dynamic. We focus on how the use of the ARTIS database alongside causal inference provides a first step toward understanding this relationship and we are more explicit that we find decreases in exports and increases in imports over time. The full revised abstract is copied below.

Lines 39-53: Global shark mortality continues to rise despite increasing regulation aimed at limiting shark fishing and international trade. Regulatory measures on shark fishing can interact with and influence trade, but this relationship has been difficult to quantify due to fragmented and poorly resolved data. To address this gap, we present a global network analysis evaluating the impact of domestic finning regulations on trade in shark products across 188 countries. Causal inference analysis of the Aquatic Resource Trade in Species database (2007-2020)

reveals weak and insignificant effects of finning regulations on trade patterns overall. Trends over time suggest that countries adopting finning regulations tend to lower exports and domestic consumption over time and eventually rely more strongly on imports to satisfy local demand. These results suggest that finning regulations may have changed trade flows in shark products between individual countries but had little effect on global trade volumes. Our approach may provide a more general framework for uncovering underlying economic pathways and drivers of mortality for sharks and other traded wildlife.

In the discussion, you note the likely possibility that other global regulations like CITES- which affect countries with anti-finning regulations and those without anti-finning regulations equally- may have had a big impact here on the shark product trade. I agree that this is plausible, but believe it should be stressed in the abstract as a likely alternative explanation. Most people will only read the abstract, and as written it creates a false impression about what happened here.
As the abstract is limited to 150 words, we did not include specific alternative explanations for our findings. However, in line with the comment above we have revised the abstract's framing and have more explicitly stated our findings to avoid any false impressions about the study.

I would like to see some discussion of why the authors are confident in their results and interpretation despite a lack of statistical significance. They're making a potentially quite impactful claim, that a suite of conservation policy tools that the environmental community lobbied hard for caused some unexpected environmental harms. Strong claims require strong evidence and a lack of significance, along with the plausible alternative explanations proposed here, do not necessarily constitute strong evidence.
We appreciate this suggestion as it makes it clear our discussion around the results and their interpretation was not clear. As you've pointed out, our evidence does not constitute strong evidence nor does it support the hypothesis that shark finning regulations have expanded markets for shark meat. We have made substantial revisions throughout including to the abstract, introduction, and discussion to be clearer about our results and the interpretation.

The abstract no longer poses the hypothesis that finning regulations have led to increases in shark trade. We instead focus on data gaps that have made it difficult to relate regulatory measures to trade, which is critical for understanding drivers of shark mortality. We position our study as taking a step toward understanding this relationship (or the lack thereof) and providing a framework that can be used to evaluate the impact of other regulations on shark trade as well as other vulnerable marine species.

In the introduction we revised the text to avoid making any claims about how shark finning regulations have influenced trade of shark products. Rather, we now present the three possible trade-related outcomes that could result from shark finning regulations: (1) international trade of

shark products increases (whole sharks are now retained, leading to development or expansion of global markets for shark products not previously retained and landed); (2) domestic consumption of shark meat increases (whole sharks are retained and meat is consumed locally), or (3) there is no effect on the global volume of shark meat traded (either because fisheries originally finning sharks now discard them whole at-sea, because regulations are not properly enforced, or because their effects are masked by other market trends). We further acknowledge that data limitations have precluded any global evaluations of this relationship, though existing country-specific evaluations suggest it is complicated and may be influenced by other factors such as growing seafood demand or economic incentives. The ARTIS database allows us to conduct the first global evaluation to understand how shark finning regulations may have affected global trade patterns of shark products. A copy of the revised introduction text is copied above in response to the second comment.

In the discussion, we now explicitly state that our findings do not lend strong evidence to the hypothesis that shark finning regulations have grown or expanded markets for shark meat. As the reviewer notes, our results do not find statistically significant increases in shark meat exports, domestic consumption, or imports following the adoption of finning regulations. The absence of a detectable regulatory impact supports that factors other than regulation are the dominant driver of shark meat trade. Our results imply decreases over time in shark landings, with countries instead turning to imported shark products to meet local demand. These results are consistent with those from Pincinato et al. (2022), the only other study that has tested this explicitly. We have restructured the rest of the discussion to provide alternative explanations for our results. First, we discuss important data limitations that could influence our findings (e.g., under-reporting/misreporting of landings, lags between adoption and implementation, other regulations). We then discuss how our results provide limited support for the other prevailing hypothesis that finning regulations have led to higher rates of discarding in favor of retaining target species. However, we note that we have not explicitly tested this hypothesis and therefore caution against over-interpretation of these findings, particularly because there could be alternative explanations. Lastly, we discuss the broader applicability of our methodology for uncovering underlying economic drivers of mortality, both for sharks and possibly other vulnerable marine species that are internationally traded. The revised discussion reads:

Lines 354-488: This study is the first to isolate the effects of shark finning regulations on prevailing trade patterns for shark products at a global scale. We found that in the years immediately following the adoption of finning regulations, there were relatively minimal changes in exporting and importing trends in countries with regulations and overall little impact on the prevailing trade network. Nine years or more after regulations were adopted, trends in trade and shifts in the network became more noticeable. Over time, countries with regulations exhibited declines in exports and domestic consumption and increases in imports compared to countries without regulations. Similarly, we found that countries with regulations contributed less to global

trade (decreasing out-degree strength) and became more reliant on imports (increasing in-degree strength) over time. Overall, shark finning regulations had little effect in the short-term on the proportion of trade partners in countries with regulations. While results indicate a negligible difference, on average, between countries with and without regulations, there may be evidence for fewer exporting (decreasing out-degree centrality) and more importing trade partnerships (increasing in-degree centrality) in the long-term. These patterns, while not statistically significant, are not consistent with the hypothesis that finning regulations have led to the establishment or expansion of markets for shark meat. Rather, they imply that reported domestic shark landings likely decreased over time after finning regulations were adopted and countries with regulations instead turned to imported shark products to meet local demand. This result is consistent with local observations in the shark meat market of Brazil, which exhibited substantial growth in imported shark meat driven by high domestic demand and declining local landings of shark meat [12].

The lack of statistical significance in our results suggests weak effects of shark finning regulations on global trade flows in shark meat, notwithstanding the trends discussed above. It may also point towards some data limitations in our analysis. First, while the ARTIS database represents important progress toward higher resolution trade data we could not isolate the effects of finning regulations on the trade in shark fins, which shows some signs of decline ([8]). Shark fins were not reported under separate commodity codes until the 2012 harmonized system (HS) for dried shark fins and 2017 HS version for fresh and frozen shark fins. Given most shark finning regulations went into effect by 2013, we lacked adequate time series data to evaluate trade under these HS versions. In addition, poor taxonomic reporting of shark landings remains a critical challenge. Since finning regulations apply to only true sharks (Selachii), saw- and wedgefishes (Rhinopristiformes), our analysis excluded any data reported as Elasmobranchii, where the volume of sharks versus skates and rays could not be distinguished. This precluded some countries that have been reported as key shark exporters (Japan and India) or consumers (India and Nigeria) [10] from being analyzed. Additionally, some countries that were included, such as Taiwan and Brazil, had only part of their trade data included as significant proportions of exports and domestic consumption, respectively, were reported as Elasmobranchii. Further, domestic consumption is not formally reported and ARTIS relies on production data reported to the FAO to estimate apparent domestic consumption, assuming that whatever is domestically-sourced but not exported must be consumed locally [18]. Misreporting and under-reporting of sharks often occurs in national datasets and in local markets [16, 17], which could result in biased estimates of apparent domestic consumption.

Our results could also be influenced by the inherent qualities of countries that choose to implement shark finning regulations. For example, countries that choose to implement strong shark finning regulations may already manage their fisheries with sustainability or conservation objectives in mind (e.g., the Maldives have adopted several regulations to protect sharks and support dive tourism [20]) or may already play an insignificant role in shark fishing globally. The implementation of a shark finning regulation is unlikely to change fishing behavior or impact

trade if, for example, a country already lands whole sharks (e.g., retained targeted shark fisheries in Indonesia [21]) or does not regularly participate in shark fisheries (e.g., Lithuania). In addition, the lag between regulation adoption and a notable change in the prevailing trade network could also be a function of the products that are traded; if traded products are shelf-stable (e.g., dried shark products) and stored until there is a demand, it may take several years to see a clear signal in international trade metrics.

Finally, we assessed the influence of shark finning regulations given their prevalence globally and broad applicability to all shark species; however, other regulations adopted during the assessed time period (2007-2020) may have more significant impacts on trade flows or domestic consumption than shark finning regulations alone. Regulations that control global trade of endangered species and their byproducts (i.e., CITES), or trade regulations that prohibit imports or exports of shark products from specific countries, may serve as primary driving factors in temporal trends of shark trade and domestic consumption. Throughout the time series, 42 shark species in the ARTIS database were listed under CITES and 29 countries that adopted shark finning regulations also adopted export bans on all shark products. While recent work finds that CITES species are still regularly traded several years after being listed for global protection [8, 13, 16], the impacts of these regulations on global trade flows has not been assessed. The DiD approach used in this study requires the staggered adoption of a policy across countries over time. Since CITES listings operate at the global scale and all countries are affected at the same time when a species is listed, the DiD approach used here is not suitable. Evaluating the impact of CITES listings would require a different methodological approach and constitutes a promising direction for future research.

Even after they are adopted, most regulations take several years to be implemented and enforced, which may result in a temporal lag between when finning regulations are adopted domestically, when fishing behavior actually changes, and, subsequently when shifts in trade patterns are detectable. We found stronger changes in imports and exports for countries that adopted shark finning regulations early in the time series, which allowed for more years of data to be evaluated after regulations were adopted. A re-evaluation of updated metrics in the coming years could still yield significant results.

Our modeling efforts provide some support for the hypothesis that shark finning regulations have disincentivized some countries from retaining sharks altogether, potentially resulting in higher rates of shark discards (sharks that are caught and released, either alive or dead). Increasing rates of shark discards could play a role in the continued rise in shark mortality despite the apparent decreases in global shark landings indicated in the trade data. Discarded catch is not always reported or included in national statistics but can be associated with high at-vessel and post-release mortality for many species (e.g., [22–24]). Evaluating the shark mortality data provided by Worm et al. [7], we calculated a slight global decrease in retained (landed) shark mortality from 53.2 million sharks in 2012 to 52.2 million in 2019, which is more than offset by an increase in discarded shark mortality from 23.1 million in 2012 to 26.2 million in 2019. Importantly, we did not directly test whether finning regulations have caused

higher discard rates for incidentally captured sharks. Future research could further investigate the landscape of discarded shark catch and potential regulatory actions to reduce associated mortality.

This study serves as a stepping stone towards a better understanding of how regulatory measures, like finning regulations, interact with and influence global trade networks and flows. Our analysis supports previous work showing that the shark trade is dominated by a few key nations such as Spain, Italy, China, Indonesia, Brazil, and Argentina [10, 13, 14]. We also show that exports, imports and domestic consumption of shark meat did not change significantly with the imposition of shark finning regulations, on average. Assuming that these regulations have been properly enforced, this suggests that sharks that were previously finned are now more likely to be discarded whole rather than retained. This trend may be influenced further by retention bans for threatened shark species that have been increasingly implemented in pelagic fisheries regulated by Regional Fisheries Management Organizations (RFMOs) and an observed reduction in pelagic shark mortality in these fisheries [7]. Interestingly, pelagic sharks were the only group in our habitat-specific analyses that showed a significant decline in exports (Table S9), which again is consistent with increased discarding of these species. However, many shark species show low survival to discarding [22], necessitating additional management measures limiting the incidence of capture for these species. An increasing proportion of the global demand for shark products may now come from coastal species, many of which are threatened, and which urgently require improved domestic regulations such as catch limits and spatial protections [4].

The methodologies used here might also be more broadly applicable. ARTIS is the first publicly accessible database that tracks the global flow of species/species group capture and aquaculture products, starting from the point of production, through global trade networks, and ending in consumption. These data enable an understanding of how management has affected trade and yield insights that are critical to uncovering underlying economic drivers of mortality, both for sharks and possibly other vulnerable marine species that are internationally traded. By uncovering the linkages in capture and aquaculture supply chains, the ARTIS database provides an opportunity to understand what incentivizes the targeting of vulnerable marine species in the first place. The analytical framework presented here can be applied to understand the influence of other regulations on shark trade as well as the trade of any wildlife or plant-based commodity that is subject to changing regulatory regimes, providing potentially new insights into a major driver of global biodiversity loss [25].

Finally, I would really encourage the authors to have a detailed, thorough, and actionable so what now what section. If these long lobbied for policies cause harm, what should policymakers do instead?

Thank you for this suggestion. In line with this and the above comment, we have revised and restructured the Discussion to provide additional clarity around our findings, the limitations of our analysis, and policy implications. We now explicitly acknowledge that our findings do not

support the hypothesis that shark finning regulations have caused growth in shark meat markets. While our results suggest weak effects of shark finning regulations on global trade flows in shark meat, we discuss areas where further research may yield important insights into drivers of shark mortality and potential policies to address them. A copy of the revised Discussion section is reproduced above.

Overall, the authors have done some important work here, and I believe it has the potential to strengthen global conservation regulations. But there is serious potential for misunderstanding as written, and given the importance of this topic, it is vitally important to be clear about what you found, did not find, and might have found but we can't tell because of data limitations.
Thank you for your thoughtful review of our manuscript. In line with previous comments, we have made substantial revisions throughout the manuscript Abstract, Introduction, and Discussion to ensure that our results and interpretations of those results are clear and we feel the manuscript is much stronger as a result.

Reviewer 4
The main concerns from reviewer #4 were that the manuscript (1) did not adequately outline the motivation for using network analysis to evaluate trade flows, and (2) lacked adequate justification for the use of node strength as opposed to other potential network analysis metrics. We have substantially revised the manuscript to address these concerns. We first provide a summary of major changes and then provide a detailed point-by-point response to each comment. A marked version of the manuscript is included in the uploaded documents which shows all additions in red text and all deletions as strikethroughs.

Summary of major changes:

1. We have revised the Introduction and Results to motivate the use of network analysis to understand global shark trade as a whole and explore the connectivity between participating countries.
2. We have expanded the suite of network metrics evaluated and revised the Results and Methods sections to include both strength and centrality measures as well as density and modularity. This has greatly improved the strength of the network analysis and the potential impact of the manuscript.

Point-by-point response
This paper employs network analysis and difference in differences to analyze the influence of shark finning regulations on the live-weight equivalent of trade and consumption of shark products. The paper utilized the Aquatic Resource Trade in Species (ARTIS) database and a mass-balance approach to estimate the live-weight trade of shark meat (excluding all shark co-products) in all shark species (excluding rays and

any products reported as "elasmobranchii"). The authors indicate that their findings provide the first global shark trade and consumption analysis that isolates the effects that shark finning regulations on both the live-weight equivalent of shark products that are traded and consumed, and the prevailing trade patterns for shark products. The authors acknowledge that while their results are not statistically significant, their study underscores the need for more careful investigations of impacts from regulation on trade flows for vulnerable species. I found the approach adopted by the authors interesting, particularly when considering that most studies on this subject tend to focus on national/regional contexts with too few looking at the macro level.
Thank you for your positive and constructive feedback on the manuscript.

I found the use of network analysis in this context to be interesting and appropriate to understand the global shark trade and consumption network. However, the written text regarding how network analysis is used left me with a few questions about the motivation and use of network analysis here. Network analysis was employed primarily to identify the "node strength", with it being the key metric of interest. Node strength is calculated here as the sum of edge weights. However trade data is directional, so the authors might want to consider using in and out degree strength or justify why the size of the trade is sufficient.
Thank you for this suggestion. We have revised the network metrics to use in-degree and out-degree strength instead of node strength, as well as to be more explicit about using a directed and weighted network. This is now clarified in the Methods (reproduced below), as well as throughout the manuscript Results and Discussion as appropriate.

Lines 570-591: To understand whether differences in trade flows occurred after shark finning regulations were adopted, we ran a directed, weighted network analysis for each year of data. Network analyses have frequently been used in previous research analyzing global trade flows [27–29]. In our network analysis, each country was modeled as a node, and the trade transaction (the live-weight equivalent of shark products traded from one country to another) was modeled as an edge. Since the trade between countries is not always reciprocal, the network was modeled as a directed network, meaning the direction of trade flows (e.g., from A to B) is accounted for. Edges were weighted by the volume of shark products traded from one country to the other. For each node, in each year, we calculated in-degree and out-degree strength and in-degree and out-degree centrality (see Table S1). In-degree strength quantifies the total weight of all incoming edges to a node and out-degree strength quantifies the total weight of all outgoing edges from a node [19]. Formally, the in-degree (s_i^{in}) and out-degree (s_i^{out}) strength of country i are defined as [30]:

$$s_i^{in} = \sum_{j \in N} w_{ji} \qquad s_i^{out} = \sum_{j \in N} w_{ij}$$

where w_{ji} denotes the weight of the incoming edge representing the trade flow from country j to country i, w_{ij} denotes the weight of the outgoing edge representing the trade flow from country i to country j, and N is the set of all countries in the network.

[30] Newman, M.E.J.: Analysis of weighted networks. Physical Review E 70(5), 056131 (2004) https://doi.org/10.1103/PhysRevE.70.05613. Publisher: American Physical Society.

Network analysis also provides other variables that might be of interest in this study such as different measures: density, modularity and centrality (degree, betweenness, closeness and eigenvector). Have the authors considered including other network analysis metrics which could help provide a more detailed picture of global shark trade and consumption dynamics?

We appreciate the suggestions. In addition to updating to in- and out-degree strength our analysis now includes in-degree and out-degree centrality as well as density and modularity. We run an additional DiD analysis on in- and out-degree centrality and have included these in the Results and Methods, as well as all relevant Supplementary Information (tables and figures). As density and modularity are network-level parameters we did not run a DiD analysis for these, but we include them in the Results and Methods for the network analysis as well as new supplementary figures of the changes in these network metrics over time (Figure S8-S9). Relevant Results and Methods text is reproduced below along with Figures S8-S9.

Lines 194-237: We used a network analysis to explore patterns of connectivity between shark-trading nations. In each year, we evaluated the in- and out-degree strength and normalized in- and out-degree centrality of each country [19] (see Table S1 for full definitions). Annual network analyses metrics for countries under different finning regulations are shown in Figures S4-S7 and annual statistics by country can be found in Supplementary Data 2. In-degree strength measures total imports to a country and reflects how dependent a country is on foreign goods. Normalized in-degree centrality is a relative measure of how well connected and central a country is within the network. It is measured as the number of trade partners a country imports from relative to all possible countries it could import from. Conversely, out-degree strength measures total exports from a country and reflects how much a country contributes to global trade. The normalized out-degree centrality is the relative importance of a country as an exporter within the network. It is measured as the number of trading partners a country exports to relative to all possible countries it could export to. In each year, we also calculated density and modularity for the global network as a whole to assess the density of trade connections and community structure (Table S1). Finally, we assess a visual representation of the largest annual trade flows at three check-points within the dataset: 2007, 2014, and 2020 (Figure 3).

Italy and Spain were consistently among the countries with highest in-degree strength

from 2007-2020, indicating sustained reliance on imported shark products over time. Brazil, China, and Portugal were also among the countries with highest in-degree strength in most years. Spain, France, Italy, Japan, and China were among the countries with highest in-degree centrality each year, suggesting they are the most well connected importing countries. For example, Spain's average in-degree centrality value was 0.22 meaning that, on average, they imported from 22% of all countries exporting shark products. Countries with both high in-degree strength and centrality, like Spain and China, are critical hubs in the trade network, importing high volumes of shark products from a large proportion of exporting countries (22% on average in both countries).

Spain, New Zealand, and Portugal consistently showed among the highest out-degree strength from 2007-2020, indicating they contribute the largest volumes of shark products to the global market. Other important exporting countries included the United States, Indonesia, and Argentina. Argentina, Spain, and New Zealand were among the countries with highest out-degree centrality in each year, suggesting they are the most well connected exporting countries. Spain had the highest out-degree strength every year and among the highest out-degree centrality, indicating high importance within the global network both in terms of volume and connectivity. On average, Spain exported to nearly half of all countries importing shark products.

As a whole, between 2007 and 2020 the density of the global shark trade network decreased slightly from 0.05 to 0.04 (Figure S8) indicating persistently sparse connectivity with fewer active country-to-country trade partnerships over time. At the same time, network modularity increased slightly from 0.38 in 2007 to 0.46 in 2020 (Figure S9) signifying a shift toward more regionally distinct trading clusters.

Lines 592-641: In-degree and out-degree centrality captures the number of trade partners a country imports from or exports to, respectively. The in- out-degree centrality is normalized to enable comparison between the measures. The normalized values reflect the proportion of trading partners a country has relative to the total amount of partners they could have [19]. Formally, the in-degree (C_i^{in}) and out-degree (C_i^{out}) centrality of country i are defined as [31]:

$$C_i^{in} = \frac{k_i^{in}}{n-1} \qquad C_i^{out} = \frac{k_i^{out}}{n-1}$$

where k_i^{in} and k_i^{out} are the number of incoming edges to country i and outgoing edges from country i, respectively, and n is the total number of countries in the network. k_i^{in} and k_i^{out} are further defined as [31]:

$$k_i^{in} = \sum_{j \in N} A_{ji} \qquad k_i^{out} = \sum_{j \in N} A_{ij}$$

where A is an adjacency indicator, A_{ji} is one if an incoming edge from country j to country i exists and zero otherwise, while A_{ij} is one if an outgoing edge from country i to country j exists

and is zero otherwise, and N is the set of all countries in the network. Normalized values of C_i^{in} and C_i^{out} range from 0-1, where zero means country i does not import from or export to any other countries, respectively, and one means country i imports from or exports to all possible countries, respectively.

Density and modularity were calculated each year for the network as a whole. Density measures the overall connectivity of a network by calculating how many trade partnerships between countries exist compared to how many could possibly exist. Density was calculated as [32]:

$$D = \frac{m}{n(n-1)}$$

where m is the total number of edges in the graph and n is the number of countries, n−1 represents the maximum number of countries that can be connected to any single country.

Modularity measures how well a network can be divided into clusters by quantifying the density of connections within a cluster and between clusters, relative to a random network [19]. Modularity values range from -1 to 1, with higher scores indicating modular networks with regionally driven trade patterns and lower scores representing less modular networks that are highly globally dispersed. Since modularity is concerned with understand connections for all trade (both imported and exported), an undirected version of the trade network was used. The modularity score Q was calculated as [30]:

$$Q = \frac{1}{2m}\sum_{i,j}[w_{ij} - \frac{s_i s_j}{2m}]\delta(c_i, c_j)$$

where w_{ij} is the weight of the edge between countries i and j, s_i and s_j is the sum of all weights of the edges connected to countries i and j, c_i and c_j is the community to which countries i and j are assigned, the δ(u, u) function is one when countries i and j are in the same community (c_i=c_j) and zero otherwise and m is the total number of edges in the network. Community structure was optimized using the Louvain algorithm [33] and all network metrics were calculated using the igraph package [34, 35].

Added references:

[31] Opsahl, T., Agneessens, F., Skvoretz, J.: Node centrality in weighted networks: Generalizing degree and shortest paths. Social Networks 32(3), 245–251 (2010) https://doi.org/10.1016/j.socnet.2010.03.006.

[32] Stanley Wasserman, Katherine Faust: Social Network Analysis: Methods and Applications. Structural Analysis in the Social Sciences. Cambridge University Press. 1994.

[33] Blondel, V.D., Guillaume, J.-L., Lambiotte, R., Lefebvre, E.: Fast unfolding of communities in large networks. Journal of Statistical Mechanics: Theory and Experiment 2008(10), 10008 (2008) https://doi.org/10.1088/1742-5468/2008/10/P10008.
[34] Antonov, M., Cs´ardi, G., Horv´at, S., M¨uller, K., Nepusz, T., Noom, D., Salmon, M., Traag, V., Welles, B.F., Zanini, F.: igraph enables fast and robust network analysis across programming languages. arXiv. arXiv:2311.10260 [cs] (2023). https://doi.org/10.48550/arXiv.2311.10260. http://arxiv.org/abs/2311.

[35] Cs´ardi, G., Nepusz, T., M¨uller, K., Horv´at, S., Traag, V., Zanini, F., Noom, D.: igraph for R: R interface of the igraph library for graph theory and network analysis (v2.1.4). Zenodo (2025). https://doi.org/10.5281/zenodo.14736815.

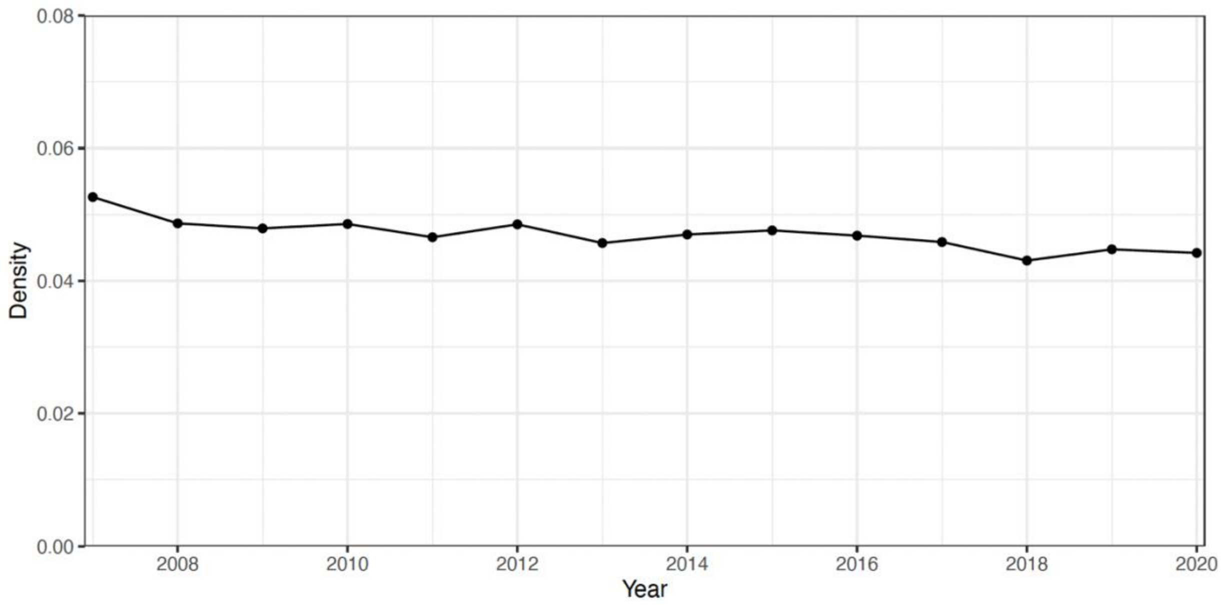

Figure S8: Time series of network density. Density is calculated for the network as a whole and reflects the number of active country-to-country trade partnerships relative to all possible trade partnerships. See Table S1 for full definitions.

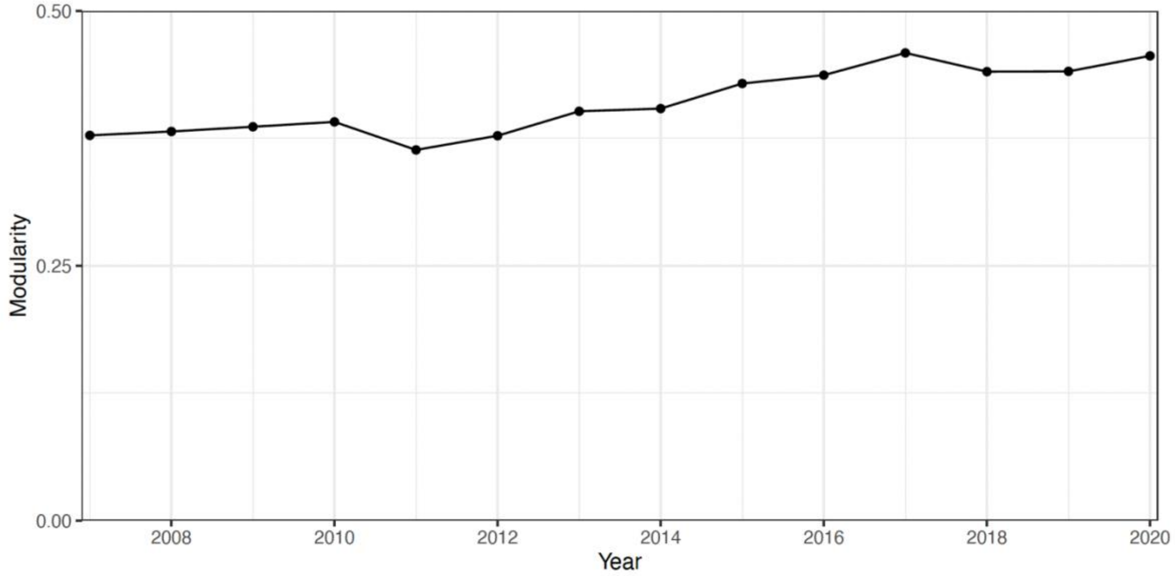

Figure S9: Time series of network modularity. Modularity is calculated for an undirected version of the network that considers total volume of shark products traded between countries but does not distinguish between imports and exports. Modularity values can range from -1 to 1, with high modularity signifying regionally clustered trade partnerships and low modularity representing a diverse global network with few distinct groups. See Table S1 for full definitions.

On Line 349 – 352, the authors recognize that the implementation of regulation is unlikely to change fisher behavior and impact shark trade flows or consumption if country already lands whole sharks or regularly participates in shark fisheries. Would the results be different if the analysis were limited to the countries that have consistent catches or significant players in the trade of shark products? Network metrics like modularity and centrality might also contribute to the explanation of this.

Thank you for this suggestion. We conducted an additional robustness check where we limited the group of treatment countries to only those countries that landed sharks in 10 years of the 14 year study period and then completed the same set of difference-in-difference analyses (on export, import, and consumption volumes, in- and out-degree strength, and in- and out-degree centrality). The results did not change the sign or significance and were qualitatively consistent with the full model results, suggesting that while this impacts the size of the treatment effect it does not change the conclusions. We include these results in Supplementary Information as Table S7, reproduced below, and reference them alongside the other robustness checks in the Results.

Lines 268-282: We also assessed the credibility of our findings through six sets of robustness checks, by 1) limiting the treatment group to only countries that adopted the strongest finning regulation (fins naturally attached), 2) limiting the control group to countries not yet regulated at

the time, but which eventually adopt similar legislation, 3) limiting the treatment group to only countries that consistently land shark products, 4) stratifying the countries by baseline trade volumes of shark products, 5) differentiating species groups occupying different habitats (i.e., coastal, pelagic, deep-sea sharks), and 6) including additional controls for a countries' population size and per-capita income. Consistency between the results of the primary model and robustness checks ensure that the reported outcomes are not driven by a specific finning regulation, by countries that never implement a regulation, by countries that do not consistently trade shark products, by countries that contribute the most to the shark trade, by species groups from a particular habitat, or by a country's population and income. Detailed regression results are presented in the Supplementary Information (Tables S5-S10) and are consistent with our main results.

Table S7: Aggregated ATT estimates across trade outcomes after limiting the control group to countries with shark catches in at least 10 years of the 14 year time series. All models include country (iso3c) and year fixed effects and control for the World Bank Governance Index and region-specific linear trends. Standard errors are clustered at the country level.

Model:	Exports (1)	Consumption (2)	Imports (3)	In-degree strength (4)	Out-degree strength (5)	In-degree centrality (6)	Out-degree centrality (7)
Variables							
ATT	-123.4	-1,012.4	300.5	231.7	-123.1	0.004	0.003
	(270.1)	(784.4)	(262.8)	(242.8)	(270.0)	(0.003)	(0.008)
Fixed-effects							
iso3c	Yes	Yes	Yes	Yes	Yes	Yes	Yes
year	Yes	Yes	Yes	Yes	Yes	Yes	Yes
Fit statistics							
Observations	961	1,045	1,003	1,003	961	1,003	961
R^2	0.96789	0.84922	0.94116	0.93063	0.96790	0.96866	0.94353
Within R^2	0.10021	0.04225	0.14522	0.14082	0.10027	0.20960	0.25137

Clustered (iso3c) standard-errors in parentheses
*Signif. Codes: ***: 0.01, **: 0.05, *: 0.1*

Some other minor language style and errors that I would like to point out. Firstly, there were a few instances where citations were written in this format: "was conducted by [8]" (line 97); "recent research by [13] and [14]" (line 101). This reads awkwardly with the Nature referencing style.

We have revised the Introduction substantially and these specific sentences are no longer included. We have also gone through and ensured that in any similar cases, the author name appears before the reference. For example:

Lines 448-452: Evaluating the shark mortality data provided by Worm et al. [7], we calculated a slight global decrease in retained (landed) shark mortality from 53.2 million sharks in 2012 to 52.2 million in 2019, which is more than offset by an increase in discarded shark mortality from 23.1 million in 2012 to 26.2 million in 2019.

Secondly, lines 417, 418, and 420, mention "FAA" regulations. FAA is not defined. Only FNA and FCR acronyms are defined in the manuscript.

We have updated the methods to ensure FAA is defined, the relevant text now reads:

Lines 504-511: Regulations are categorized by strongest to weakest regulation as: 1) shark sanctuaries where all shark fishing is prohibited; 2) fins naturally attached (FNA), where all landed sharks must be whole with their fins attached; 3) fins artificially attached (FAA), where caught sharks may have their fins removed for storage, but re-attached via ropes or wires prior to landing; 4) fin-to-carcass ratio (FCR), where shark fins must be landed alongside shark carcasses in a defined ratio; or 5) unspecified finning regulations where there is some form of prohibition but its definition is not clearly documented.

RESPONSE TO REVIEWERS
Manuscript number: NCOMMS-25-60414A
"Investigating the relationship between shark finning regulations and the trade of shark products"
Formerly, *"The impact of shark finning regulations on the trade of shark products"*
April 20, 2026

Reviewer 1
We appreciate the substantial revisions and re-analyses that the authors undertook in response to the reviewer comments.
Thank you for taking the time to review both our initial and revised manuscript, we appreciate your thoughtful comments and suggestions.

Although we did note their efforts to simply terminology and streamline the paper, it is still extremely convoluted and difficult to follow. Ultimately, our earlier concerns are unresolved despite these efforts. Their reported results do not align with the conclusions. Specifically, the authors report that in countries with anti-finning regulations, exports decreased (after 7 years of no change), imports changed a negligible amount, and domestic consumption increased (after 7 years of a decline). Despite their own reported DID results, they use the node strength/network analyses to come to divergent conclusions: that countries with shark finning regulations contributed less to exports and became more reliant on imports. They conclude that L513 " reported domestic shark landings likely decreased over time after finning regulations were adopted and countries with regulations instead turned to imported shark". This is contradicted by their own analyses of ARTIS data/DID analyses.
Thank you for this feedback. In the following paragraphs, we will summarize how we have incorporated your feedback by restructuring the analysis and clarifying our results to avoid the points of confusion highlighted in your review. In summary: 1) in order to further streamline and clarify our analyses and results, we have restructured the results to explicitly separate the analysis on trade outcomes from the analysis on network outcomes; and 2) we then clearly articulate how and why the trade outcome and network outcome results are in fact consistent with each other, not contradictory.

We have rewritten our results section to first provide an overview of shark product trade ("Global shark product trade by volume"), then the difference-in-difference (DiD) results for the three trade outcomes: exports, imports, and domestic consumption in "Impact of finning regulations on the volume of traded shark products". Additionally, we agree that the presentation of all trade-relevant statistical results within the same paragraph introduced points of confusion. To address this, we now present the time trends and relevant statistics (treatment effect, 95% confidence interval, and p-value) for each of the trade outcomes individually to improve readability and clarity. The main figure for this section (Figure 3) now contains the event study plots for the three trade outcomes only (the event study plots related to the network analyses have been removed); the statistical results for these three trade analyses are presented in

Supplementary Tables 3-4, the robustness checks are included as Supplementary Tables 5-10, and the sensitivity analysis (leave-one-out) results are in Supplementary Table 11.

Results from the shark trade network analysis are now reported separately in “Key actors and relationships in the global shark trade network”, with the DiD results for normalized in-degree and out-degree centrality now contained within “Impact of finning regulations on shark trade network relationships”. The normalized centrality represents the proportion of trading partners a country has relative to the total amount of partners they could have. In-degree centrality looks at importing partnerships and out-degree centrality looks at exporting partnerships. We have included the event study plots in their own unique figure (Figure 5); statistical results are now presented in Supplementary Tables 12-13, the robustness checks are included as Supplementary Tables 15-20, and the sensitivity analysis (leave-one-out) results are presented in Supplementary Table 21. In-degree strength is a network measure of total imports and out-degree strength is a network measure of total exports. As both metrics are already described by the sum of imports and exports described in the previous section (“Impact of finning regulations on the volume of traded shark products”), we have removed these results from the main text and added them to the Supplementary Information (Supplementary Fig. 12, Supplementary Table 14). We thank the reviewer for noting this point of confusion in the text.

By rewriting the results section in this way, we are also able to clarify that our analyses for trade outcomes and the global shark trade network are not contradictory but are in fact qualitatively consistent. In the shark trade network, each country is represented as a node and the trade transactions between countries (live-weight equivalent of shark products traded) are represented as edges. In network analysis, the in-degree strength is the sum of the weights of all incoming edges to a node; this is another way to represent the total imports to a given country. We would expect that the DiD results for in-degree strength closely match those reported for imports. Indeed, the patterns observed in the in-degree strength DiD analysis (now Supplementary Fig. 12a, reproduced below on the left) do follow the same temporal trends as those observed in the analysis of imports (Figure 3b, reproduced below on the right). Both measures show limited change in the first several years after regulation (right of the dashed line) with a slight positive trend 8-11 years post-regulation. Both analyses have comparable estimated average treatment effects (In-degree strength: 103 mt per year, Supplementary Table 14; Imports: 142 mt per year, Supplementary Table 3) showing that countries with regulations, on average, import more shark products than they would have without regulations. In both cases, we find no evidence that this change is significant (In-degree strength $p = 0.623$; Imports $p = 0.538$).

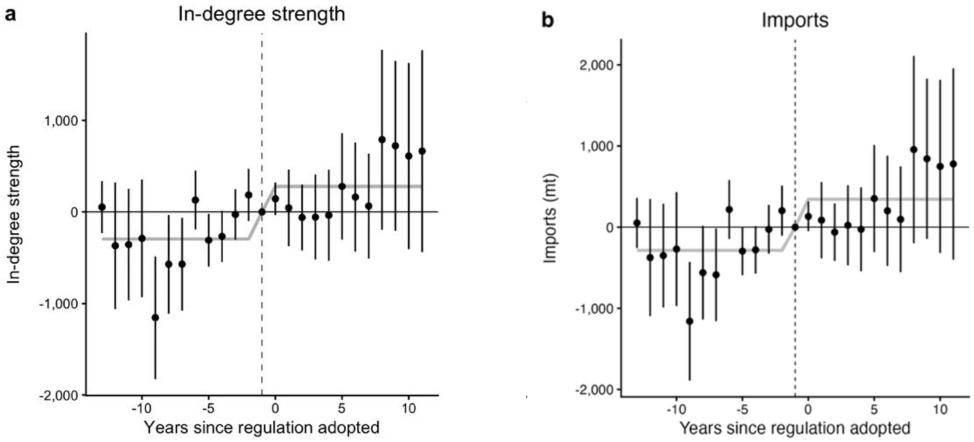

We find a similar level of consistency for exports. Out-degree strength is the sum of the weights of all outgoing edges to a node, which is another way to represent the total exports from a given country. We would expect the DiD results for out-degree strength (Supplementary Fig. 12b, reproduced below on the left) to mirror those reported for exports (Figure 3a, reproduced below on the right). In both cases, we observe relatively little change in the first several years, with larger declines in later years. Both analyses have comparable estimated average treatment effects (Out-degree strength: -350 mt per year, Supplementary Table 14; Exports: -351 mt per year, Supplementary Table 3) showing that countries with regulations, on average, export less shark products than they would have without regulations. In both cases, we find no evidence that this change is significant (Out-degree strength p =0.350; Exports p = 0.350). We have updated the text to explicitly highlight how the trade outcomes and network outcomes results are consistent. The relevant text is provided below.

Line 320-323: In-degree and out-degree strength are alternative ways to represent total imports and exports, respectively, and temporal trends and treatment effects mirror the trade outcome analysis of imports and exports (Supplementary Fig. 12, Supplementary Table 14).

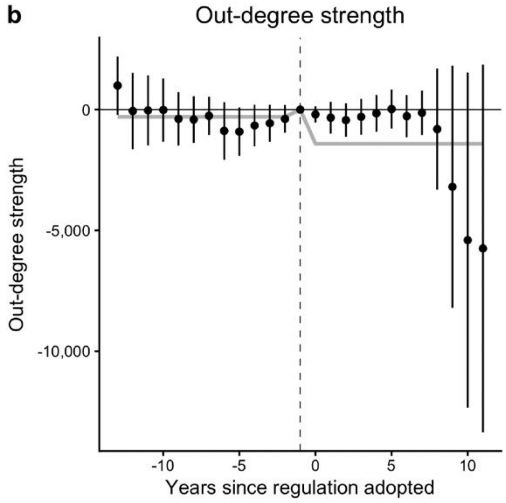

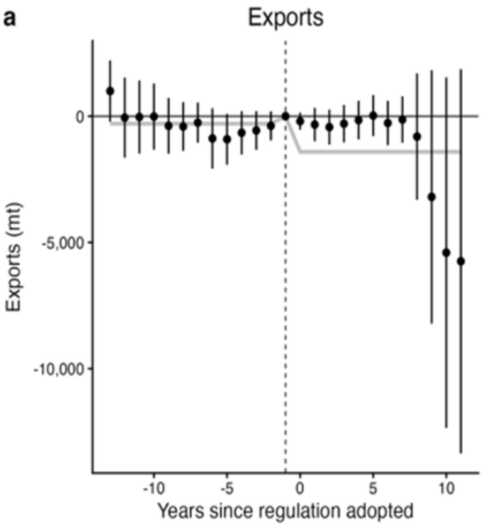

In line with the reorganization of results, we have updated the last paragraph of the introduction, reproduced below, to clarify the new structure.

Lines 135-150: Here, we leverage the ARTIS database, alongside causal inference techniques, to evaluate the effect of shark finning regulations on the 1) global trade of shark products by volume, and 2) global shark trade network relationships from 2007-2020. We first provide a brief overview of the global trade of shark products during the study period. We then analyze whether finning regulations in participating countries have a causal effect on reported volumes of: 1) traded shark products (exports and imports); and 2) apparent domestic consumption of shark products (all in metric tons, live weight equivalent; see Supplementary Table 1 for full definitions). To complement our analysis on trade outcomes, we use network analysis to understand the connectivity of countries participating in the global shark trade. We first characterize the shark trade network during the study period and then apply the same causal inference techniques to analyze whether finning regulations in participating countries have a causal impact on the number of trade partnerships. Our results may serve as a foundation to better understand the impact of regulatory interventions on global shark trade. Furthermore, the methodological approach developed in this paper and the underlying ARTIS database have broad relevance to analyses of global wildlife trade, a major threat to biodiversity worldwide.

Finally, we have updated the beginning of the discussion where we summarize the results from both the DiD analysis on trade outcomes and network outcomes. We have removed the interpretation of the in-degree and out-degree strength results since those are no longer presented in the main text and we focus on just the interpretation of the five DiD analyses presented: exports, imports, domestic consumption, in-degree centrality (proportion of countries a given country imports from), and out-degree centrality (proportion of countries a given country exports to). We present a summary sentence interpreting the trade results and the network results separately and then summarize what these results imply when taken together. The relevant updated text is reproduced below.

Lines 348-364: This study is the first to isolate the effects of shark finning regulations on prevailing trade patterns for shark products at a global scale. From a visual assessment of the data, we note that in the years immediately following the adoption of finning regulations, there were declines in the volume of shark products consumed domestically but relatively minimal changes in exporting and importing trends in countries with regulations. Nine years or more after regulations were adopted, no sustained impacts were observed for domestic consumption, but countries with regulations exhibited greater declines in exports and increases in imports compared to countries without regulations. Similarly, we found that shark finning regulations had no immediate impact on trade network relations but, over the long-term (nine years or more post-adoption), countries with regulations exported to a smaller proportion of countries (decreasing out-degree centrality) and imported from a larger proportion of countries (increasing in-degree centrality). Taken together, in the long-term, we find countries with regulations are exporting a lower volume of shark products to a smaller proportion of countries and importing a higher volume from a larger proportion of countries, while domestic consumption remains relatively unchanged. These general trends are not consistent with the hypothesis that finning regulations could have a significant positive effect on trade or consumption.

They further speculate on changes in shark discard rates which while extremely important are not captured the ARTIS database and thus should not be part of their conclusions. Domestic consumptions has increased which could mean that shark discard rates have declined but that could be a false assumption as well. This method of analysis does not consider or use discard data (see James et al 2015, doi:10.1017/S0376892915000168). We and the other reviewers were unanimous that while this paper can help understand how shark trade (imports, exports, domestic consumption) has changed over time, the framing of the paper as "The impact of shark finning regulations on the trade of shark products" is not appropriate or robust.
We agree that while discards are very important, they are not something we directly analyzed as a part of this study and that given our analysis, we cannot make any conclusions about how finning regulations affect discards. This is due to data limitations as you point out: the ARTIS database simply does not track discards. However, given that the lack of significance in our results does not provide strong evidence that finning regulations have led to the establishment or expansion of markets for shark meat, we believe it is important to discuss possible alternative hypotheses that would still be qualitatively consistent with our findings. We have revised the discussion on discards to focus only on what we have observed in our results. Our results show long-term declines in exports paired with relatively unchanged (slightly decreasing on average) domestic consumption. If domestic consumption is relatively stable and exports decline over time, it suggests that domestic production is declining. In our robustness checks, we also observe significant declines in exports of pelagic sharks, the only habitat group with statistically significant changes in any trade outcome after the adoption of finning regulations. This decrease in exports could be explained by increasing discard rates or fewer shark interactions resulting in lower shark catch. We now mention both of these as possibilities in the discussion. As pointed out, we do not use discard data and therefore are careful to explicitly acknowledge that we did not directly test whether finning regulations have caused higher discard rates for incidentally captured sharks. We have also updated this statement to clarify that ARTIS does not include

discard data and we do not quantify changes in discard rates as a part of this study. The relevant updated text is reproduced below.

Lines 420-438: The trend towards long-term declines in exports observed in our model, paired with relatively unchanged domestic consumption, indicates that fewer whole sharks are being landed and supplied to markets from countries with finning regulations. This could be due to fewer shark interactions resulting in lower overall shark catch, though this is less consistent with evidence pointing to the continued rise in global shark mortality [7]. Alternatively, shark finning regulations may have disincentivized some countries from retaining sharks altogether, and catch that was previously finned is now being discarded. This trend may be influenced further by retention bans for threatened shark species that have been increasingly implemented in pelagic fisheries regulated by Regional Fisheries Management Organizations (RFMOs) and an observed reduction in pelagic shark mortality in these fisheries [7]. Interestingly, pelagic sharks were the only group in our habitat-specific analyses that showed a significant decline in exports (Supplementary Table 9), which is consistent with decreased retention and increased discarding of incidental catch of these species. Discarded catch is not always reported or included in national statistics but can be associated with high at-vessel and post-release mortality for many species (e.g., [26-28]). Importantly, data on discarded shark catch are not included in the ARTIS database and we did not quantify discard rates or directly test whether finning regulations have caused higher discard rates for incidentally captured sharks. Future research could further investigate the landscape of discarded shark catch and potential regulatory actions to reduce associated mortality.

Further, we have removed the sentences referring to discards from the final paragraph of the discussion which made it feel more like a conclusion of work when it is in fact not, as pointed out. Instead, we focus our final paragraph of the discussion only on our explicit analysis of trade outcomes and network outcomes, summarizing that we find no evidence that trade flows and trade networks changed significantly after the adoption of finning regulations. We now conclude the manuscript by elevating the utility of the ARTIS database for evaluating the economic drivers of mortality for both sharks and other vulnerable marine species. The relevant updated discussion text is reproduced below. Finally, we have changed the manuscript title to “Investigating the relationship between shark finning regulations and the trade of shark products”.

Lines 452-470: This study serves as a stepping stone towards a better understanding of how regulatory measures, like finning regulations, interact with and influence global trade flows and networks. Our analysis supports previous work showing that the shark trade is dominated by a few key nations such as Spain, Italy, China, Indonesia, Brazil, and Argentina [10, 14, 17]. We also show that both global trade flows of shark meat (i.e., exports, imports, and domestic consumption) and trade networks (i.e., the proportion of trade partnerships) did not change significantly with the imposition of shark finning regulations, on average. The methodologies used here might also be more broadly applicable. ARTIS is the first publicly accessible database that tracks the global flow of species/species group capture and aquaculture products, starting from the point of production, through global trade networks, and ending in consumption.

These data enable an understanding of how management has affected trade and yield insights that are critical to uncovering underlying economic drivers of mortality, both for sharks and possibly other vulnerable marine species that are internationally traded. By uncovering the linkages in capture and aquaculture supply chains, the ARTIS database provides an opportunity to understand what incentivizes the targeting of vulnerable marine species in the first place. The analytical framework presented here can be applied to understand the influence of other regulations on shark trade as well as the trade of any wildlife or plant-based commodity that is subject to changing regulatory regimes, providing potentially new insights into a major driver of global biodiversity loss [33].

Reviewer #1 (Remarks on code availability):
The code is accessible
We have made a few additional revisions to the code to match the re-organization of the results. We have included a copy of the updated zip file as it appears on Zenodo as an additional file in the submission and will make the link publicly available upon acceptance.

Reviewer 2
I co-reviewed this manuscript with one of the reviewers who provided the listed reports. This is part of the Nature Communications initiative to facilitate training in peer review and to provide appropriate recognition for Early Career Researchers who co-review manuscripts.
Thank you for taking the time to review both our initial and revised manuscript, we deeply appreciate your thoughtful comments and suggestions.

Reviewer 3
The authors have thoroughly and thoughtfully responded to my suggestions from the last version of the manuscript, and from what I can see they also appear to have done the same for other comments from other referees. I am now comfortable endorsing publication, and I congratulate the authors on a timely, significant, and well-done analysis.
We appreciate your feedback and the time you've taken to review both our initial and the revised manuscript. Thank you again for your thoughtful comments which provided clear guidance that helped us strengthen the paper.

Reviewer 4
The authors have addressed most of the comments raised in the previous version, and the manuscript has improved substantially in clarity and structure. The revisions have strengthened both the methodological reasoning and the presentation of results.
Thank you for taking the time to review both our initial and revised manuscript, we deeply appreciate your thoughtful comments and suggestions.

Some minor comments are listed below:
Line 193: although not grammatically incorrect, please consider removing the "a" in "We used a network analysis ...".
We have made this change both here and in the Results section. The relevant text now reads:

Line 142-143: To complement our analysis on trade outcomes, we use network analysis to understand the connectivity of countries participating in global shark trade.

Line 263: We used network analysis to explore patterns of connectivity between shark-trading nations.

Lines 292-297: This sentence is long and may take a few reads to understand it. The authors should consider expressing it more concisely and clearly, or alternatively dividing it into shorter sentences.
We have broken this sentence apart. We now present the temporal trends followed by the relevant statistical findings (estimated treatment effect, confidence interval, p-value) for each trade outcome in turn. The relevant updated text is reproduced below.

Line 211-229: Exports of shark products in countries with regulations appear relatively stable for the first seven years after adoption. After year seven, exports tend to decline in countries with regulations compared to those without, with steeper declines observed the longer regulations have been in place (Figure 3a). On average, countries with regulations export 351 mt per year less than they would have in the absence of regulation, with a maximum difference of 5,746 mt at 11 years post-regulation. However, we find no strong statistical evidence that this trend in the volume of exported shark products by regulated countries is significant (ATT= -351 mt per year; 95% CI: -1093 to 392, p=0.350). After the adoption of finning regulations, imports of shark products continue to fluctuate around pre-adoption levels, with a slight positive trend in years 8–11 (Figure 3b). On average, countries with regulations import 142 mt more per year than they would have if they did not have regulations in place. Similarly, we find no strong evidence that this change in imports to regulated countries is significant (ATT=142 mt per year; 95 % CI: -314 to 190; p= 0.538). Unlike exports and imports which remained relatively unchanged immediately following regulation, domestic consumption of shark products appears to decline over the first seven years after the adoption of a finning regulation, though this reduction is not sustained over longer time scales (Figure 3c). On average, countries with regulations consumed 1,672 mt less shark product per year than they would have without a regulation in place but, again, we find no evidence that this change is significant (ATT=1672 mt per year; 95 % CI: -3623 to 280; p= 0.092).

We have also restructured the results to improve clarity and we present first the analysis on the volume of traded shark products and then the analysis of the shark trade network. The DiD results for the volume of traded shark products is included in “Impact of finning regulations on the volume of traded shark products” and the network analysis DiD results are now presented separately in “Impact of finning regulations on shark trade network relationships”.

Regarding the earlier commented part on in/out-degree, this was addressed and results were presented accordingly. However, some parts remain a little confusing when reading through and they may require a few reads to understand, especially for those less familiar with network analysis. For example, in the introduction, the authors referred to the trade activities as import and exports. But later, in sections 2.2-2.3, when reporting the results for the network, the authors repeatedly used “in-degree” and “out-degree”. It could be expressed more clearly that the in-degree network and out-degree network correspond to imports and exports, respectively. Something more alike Section 3 line 367-379 might be easier for readers to understand.
We have separated the analyses on shark trade and the shark trade network. For the network analysis summary (now “Key actors and relationships in the global shark trade network”), we still calculate and present results summarizing the in-degree and out-degree strength and centrality for each country and year. However, as mentioned, since in-degree strength and out-degree strength correspond to imports and exports we have removed this from the network outcome DiD results (now “Impact of finning regulations on shark trade network relationships”) to reduce confusion. We now explicitly state that in-degree and out-degree strength are different ways to represent imports and exports and, as expected, these analyses mirror the results presented for imports and exports (relevant text reproduced below). We do include the event study plot and statistical results of the in-degree and out-degree strength DiD analysis in the Supplementary Information (Supplementary Fig. 12, Supplementary Table 14) for the sake of transparency. The main network results presented in “Impact of finning regulations on shark trade network relationships” now only includes the DiD analysis results for in-degree and out-degree centrality as this is a distinct metric that is complementary to the trade outcomes.

Lines 317-325: We applied the same DiD approach to the annual country-level network metrics to assess whether shark finning regulations implemented over time have caused concomitant changes in a country’s: 1) importing trade partnerships (in-degree centrality); and 2) exporting trade partnerships (out-degree centrality; Supplementary Tables 12-13). In-degree and out-degree strength are alternative ways to represent total imports and exports, respectively, and temporal trends and treatment effects mirror the trade outcome analysis of imports and exports (Supplementary Fig. 12, Supplementary Table 14). We performed the same set of six robustness checks for in-degree and out-degree centrality and found the results were consistent with those in the primary models (Supplementary Tables 15-20).

In line with the reorganization of the results and limiting the network DiD results to centrality measures only, we have updated the final paragraph of the introduction and first paragraph of the discussion accordingly. The relevant introduction and discussion texts are reproduced below.

Line 135-150: Here, we leverage the ARTIS database, alongside causal inference techniques, to evaluate the effect of shark finning regulations on the 1) global trade of shark products by volume, and 2) global shark trade network relationships from 2007-2020. We first provide a brief overview of the global trade of shark products during the study period. We then analyze whether finning regulations in participating countries have a causal effect on reported volumes of: 1)

traded shark products (exports and imports); and 2) apparent domestic consumption of shark products (all in metric tons, live weight equivalent; see Supplementary Table 1 for full definitions). To complement our analysis on trade outcomes, we use network analysis to understand the connectivity of countries participating in the global shark trade. We first characterize the shark trade network during the study period and then apply the same causal inference techniques to analyze whether finning regulations in participating countries have a causal impact on the number of trade partnerships. Our results may serve as a foundation to better understand the impact of regulatory interventions on global shark trade. Furthermore, the methodological approach developed in this paper and the underlying ARTIS database have broad relevance to analyses of global wildlife trade, a major threat to biodiversity worldwide.

Lines 348-364: This study is the first to isolate the effects of shark finning regulations on prevailing trade patterns for shark products at a global scale. From a visual assessment of the data, we note that in the years immediately following the adoption of finning regulations, there were declines in the volume of shark products consumed domestically but relatively minimal changes in exporting and importing trends in countries with regulations. Nine years or more after regulations were adopted, no sustained impacts were observed for domestic consumption, but countries with regulations exhibited greater declines in exports and increases in imports compared to countries without regulations. Similarly, we found that shark finning regulations had no immediate impact on trade network relations but, over the long-term (nine years or more post-adoption), countries with regulations exported to a smaller proportion of countries (decreasing out-degree centrality) and imported from a larger proportion of countries (increasing in-degree centrality). Taken together, in the long-term, we find countries with regulations are exporting a lower volume of shark products to a smaller proportion of countries and importing a higher volume from a larger proportion of countries, while domestic consumption remains relatively unchanged. These general trends are not consistent with the hypothesis that finning regulations could have a significant positive effect on trade or consumption.
